# Oxidative Stress: Signaling Pathways, Biological Functions, and Disease

**DOI:** 10.1002/mco2.70268

**Published:** 2025-07-01

**Authors:** Sixuan Liu, Jiachen Liu, Yinhuai Wang, Fei Deng, Zebin Deng

**Affiliations:** ^1^ Department of Urology The Second Xiangya Hospital of Central South University Changsha Hunan China; ^2^ Xiangya School of Medicine Central South University Changsha Hunan China; ^3^ Xiangya Hospital Central South University Changsha Hunan China; ^4^ Department of Nephrology The Second Xiangya Hospital at Central South University Changsha Hunan China; ^5^ National Clinical Research Center for Metabolic Disease Key Laboratory of Diabetes Immunology (Central South University) Ministry of Education Changsha China

**Keywords:** inflammation, mitochondrial dysfunction, oxidative stress, reactive oxygen species, redox homeostasis

## Abstract

The dysregulated accumulation of reactive oxygen species (ROS) and reactive nitrogen species disrupts redox homeostasis, triggering oxidative stress (OS) and driving pathophysiological changes across multiple organ systems. OS modulates critical signaling pathways, induces inflammation, impairs mitochondrial function, alters metabolic homeostasis, and dysregulates autophagy, contributing to disease progression. While prior research has largely focused on OS within single‐organ diseases (e.g., neurodegenerative, cardiovascular, and oncological disorders), the systemic role of OS in pan‐organ diseases and interorgan communication remains insufficiently explored. This review integrates multidisciplinary evidence to elucidate the biological functions of OS in cellular signaling, homeostasis, and cross‐organ crosstalk. It systematically dissects OS‐driven molecular mechanisms and pathophysiological networks across 10 major organ systems, including the nervous, cardiovascular, oncological, hepatic, and renal systems. Furthermore, it critically examines OS‐related therapeutic targets, including antioxidant and ROS‐generating enzymes, and explores synergistic redox‐based therapeutic strategies. By moving beyond traditional single‐organ paradigms, this review constructs a holistic framework to decode the systemic impact of OS, offering novel insights into disease mechanisms and therapeutic innovations. Ultimately, it lays the foundation for precision medicine approaches aimed at mitigating OS‐driven diseases and improving multiorgan health outcomes.

## Graphical abstract

Oxidative stress causes cellular damage across multiple systems, contributing to neurodegeneration (Alzheimer's, Parkinson's, Huntington's), cancer progression and resistance, cardiovascular diseases (atherosclerosis, heart failure), liver and kidney injury, metabolic disorders (diabetes, obesity), autoimmune diseases, musculoskeletal decline, retinal disorders, and reproductive infertility.

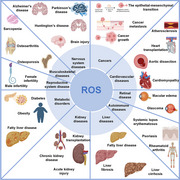

## 1 Introduction

Oxidative stress (OS) arises from an imbalance between reactive oxygen species (ROS) production and the antioxidant defense system, leading to the accumulation of ROS and resultant oxidative damage to cellular components, such as lipids, proteins, and DNA. ROS are produced during mitochondrial oxidative metabolism and in cellular responses to foreign organisms, cytokines, and bacterial invasions [1].

At physiological levels, ROS act as key signaling molecules that regulates basic biological processes such as cell proliferation, differentiation, and immune response [2, 3]. However, when ROS is produced too much or when antioxidant defenses are compromised, redox homeostasis is disrupted, triggering oxidative damage and cell dysfunction. These changes are closely related to a variety of pathological processes, including inflammation [4, 5], mitochondrial dysfunction [6, 7, 8], and autophagy [9, 10, 11], which ultimately lead to the occurrence of human diseases. The mechanisms underlying OS involve complex interactions among ROS, antioxidant systems, cellular signaling pathways, and pathological processes.

Key antioxidant molecules, such as glutathione (GSH) [12], superoxide dismutase (SOD) [13, 14], and catalase (CAT) [15, 16, 17], serve to mitigate ROS accumulation and maintain redox balance. ROS interact with specific cellular signaling cascades, such as mitogen‐activated protein kinase (MAPK) [18, 19, 20] and nuclear factor erythroid 2‐related factor 2 (Nrf2) [21, 22], influencing gene expression and cellular responses. However, excessive production of ROS or impaired antioxidant defense mechanisms can disrupt redox homeostasis, leading to oxidative damage and cellular dysfunction, which are intricately linked to various pathological processes, such as inflammation. For example, OS accelerates the progression of neurodegenerative diseases by hastening neuronal apoptosis and highlighting functional impairments [23]; it promotes the progression of cardiovascular diseases through endothelial dysfunction [24]; and it regulates cancer progression by affecting multiple signaling pathways, including Nrf2 [25, 26]. In recent years, advances in redox biology have revealed potential therapeutic targets and strategies for modulating OS, such as the use of antioxidants, ROS scavengers, and signaling pathway inhibitors, some of which are undergoing clinical evaluation.

Despite the growing body of research on OS, a comprehensive and systematic understanding of its biological functions across organ systems and its intricate interactions within physiological and pathological networks remains lacking.

This review comprehensively describes the biological functions of OS in the whole body, and discusses its broad effects at multiple levels, including signal transduction, emphasizing the central role of OS in various organ systems. On this basis, this paper systematically summarizes the specific mechanisms of OS in diseases affecting different organ systems and reveals its complex and multifaceted pathophysiological effects. Given the importance of OS, this paper further delves into its potential as a therapeutic target for the treatment of human disease, with the aim of deepening our understanding of OS and inspiring future research to translate these insights into clinical applications.

## 2 Physiological Roles of ROS

### 2.1 ROS Regulates Immune System Function

**FIGURE 1:**
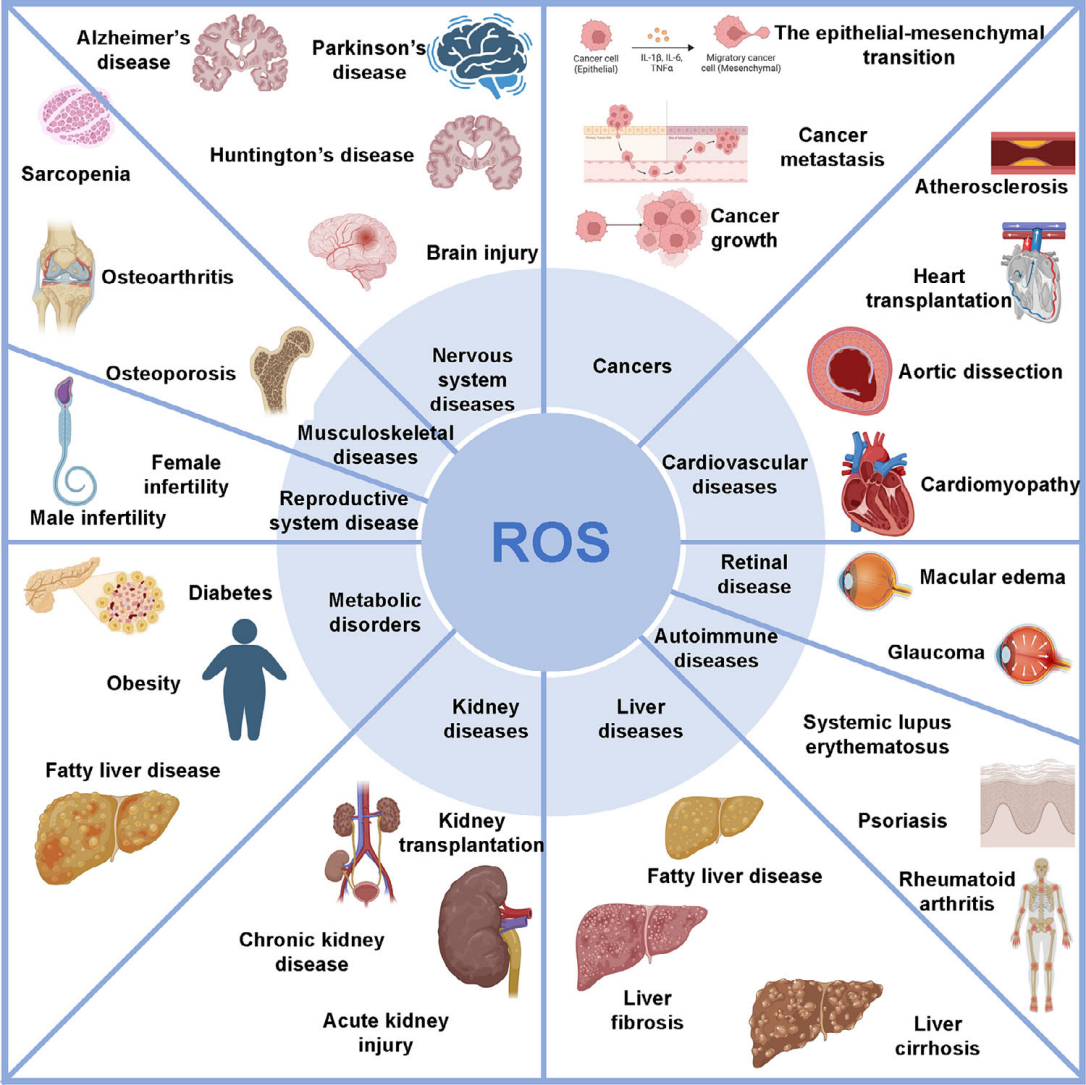
The role of ROS in immune system. In macrophages, ROS drive M1 polarization via ataxia–telangiectasia mutated (ATM)‐cell cycle checkpoint kinase 2 (Chk2)‐mediated pyruvate kinase M2 (PKM2) phosphorylation/glycolysis, MAPK‐nuclear factor kappa‐B p65 (NF‐κB p65)‐dependent cytokine secretion, and hypoxia‐inducible factor 1‐alpha (HIF‐1α)/NLR family pyrin domain‐containing 3 (NLRP3) inflammasome activation, amplified by Nrf2/NLRP3 pyroptosis and migration inhibitory factor (MIF) release. For M2 polarization, ROS suppress via p38–MAPK–mitogen‐activated protein kinase phosphatase‐1 (MKP‐1)/diacylglycerol acyltransferase 1 (DGAT1)‐lipid peroxidation but promote via extracellular regulated protein kinases (ERK)/mammalian target of rapamycin (mTOR), phosphatidylinositol‐3‐kinase (PI3K)/protein kinase B (Akt), and Nrf2/heme oxygenase‐1 (HO‐1)/mitophagy. Neutrophils employ ROS for pathogen clearance and NETosis: NOX‐dependent pathways involve phorbol myristate acetate (PMA)‐activated protein kinase C (PKC)/NADPH oxidase (NOX)/myeloperoxidase (MPO)‐generated ROS activating ERK/p38/c‐Jun N‐terminal kinase (JNK) to release neutrophil extracellular traps (NETs), while NOX‐independent pathways rely on mitochondrial permeability transition pore (mPTP)‐derived mtROS/p38‐calcium synergy. In dendritic cells, ROS regulate maturation through ERK/NF‐κB and Kelch‐like ECH‐associated protein 1 (Keap1)/Nrf2 signaling, balancing p38–MAPK activation and endoplasmic reticulum (ER) stress. ROS also enhance cross‐presentation via lysosomal ROS/calcium and mitochondrial mtROS‐dependent major histocompatibility complex I (MHC I) antigen processing. T cells utilize ROS as second messengers post‐TCR activation, driving nuclear factor of activated T cells (NFAT)/activator protein 1 (AP‐1)/NF‐κB‐mediated proliferation/cytokine production (interleukin‐2/4 [IL‐2/4]) and metabolic regulation via mtROS. B cells depend on ROS to amplify B cell receptor (BCR) signaling through protein tyrosine phosphatases (PTPs) inhibition/spleen tyrosine kinase (Syk) activation, while excess ROS induce apoptosis via mitochondrial disruption. This figure was created with BioRender (https://biorender.com/).

ROS regulate cellular redox homeostasis within the organism, and cellular redox reactions play a distinctive role in modulating immune responses (Figure 1).

#### 2.1.1 Innate Immune System

The state of OS exerts a direct influence on the functionality of immune cells through the regulation of various cellular signaling pathways. In the context of the innate immune system, the polarization and activity of macrophages are significantly modulated by the concentrations of ROS. Moreover, the formation of neutrophil extracellular traps (NETs) is similarly impacted by ROS levels. Additionally, in dendritic cells (DCs), ROS plays a pivotal role in the regulation of cross‐presentation capacity [27, 28, 29]. Despite these insights, a comprehensive understanding of the multifaceted roles of OS in immune responses remains to be fully elucidated, highlighting the need for further exploration into the interconnected mechanisms driving these processes.

##### 2.1.1.1 Macrophage

Macrophages can be broadly categorized into two main types: M1 macrophages, which are proinflammatory, and M2 macrophages, which are associated with anti‐inflammatory responses. ROS play a significant role in the polarization of macrophages, activating various regulatory pathways that influence the generation of proinflammatory factors and other immune responses.

For instance, during M1 macrophage polarization, the ROS–ataxia–telangiectasia mutated (ATM)‐cell cycle checkpoint kinase 2 (Chk2) pathway enhances the phosphorylation of pyruvate kinase M2 (PKM2), a process that subsequently drives glycolysis and metabolic reprogramming [30]. In addition, ROS are crucial for the secretion of migration inhibitory factor (MIF), which supports M1 polarization and the production of proinflammatory factors [31]. Moreover, ROS can activate the MAPK signaling pathway, leading to the release of nuclear factor kappa‐B p65 (NF‐κB p65), thereby influencing M1 macrophage polarization through the ROS–MAPK–NF‐κB signaling axis [32]. ROS also upregulate the expression of hypoxia‐inducible factor 1‐alpha (HIF‐1α) [33] and induce the activation of NLR family pyrin domain‐containing 3 (NLRP3) inflammasome [34], promoting the M1 polarization of macrophages [35]. Furthermore, the activation of the ROS/Nrf2/NLRP3 signaling pathway can trigger pyroptosis, thereby enhancing M1 macrophage polarization [36].

In a similarly vein, ROS regulates the process of M2 polarization through different signaling pathways. During M2 macrophage polarization, elevated levels of ROS can activate p38–MAPK, which promotes lipid peroxidation (LPO) through the upregulation of diacylglycerol acyltransferase 1 (DGAT1) and inhibits M2 polarization [37]. The p38–MAPK pathway mediates the inhibition of M2 polarization through suppressors of cytokine signaling (SOCS3) targeted actions on p38 and MAPK phosphatase‐1 (MKP‐1) [37, 38]. Conversely, the activation of the ROS/extracellular regulated protein kinases (ERK) and mammalian target of rapamycin (mTOR) signaling pathways facilitates M2 macrophage polarization. And studies showed that the Th2‐like cytokine IL‐25 can induce ROS production, increase mitochondrial respiratory chain complex activity, subsequently activate AMP‐activated protein kinase (AMPK), and induce mitophagy to stimulate M2 macrophage polarization in monocytes [39]. Additionally, ROS can stimulate the phosphatidylinositol‐3‐kinase (PI3K)/protein kinase B (Akt) pathway to steer M2 macrophage polarization [40]. The enhancement of the Nrf2/heme oxygenase‐1 (HO‐1) signaling pathway also exhibits anti‐inflammatory properties and modulates M2 macrophage polarization [41].

##### 2.1.1.2 Neutrophil

Neutrophils, as key effector cells of the innate immune system, are rapidly recruited to sites of infection and inflammation to mount an early defense against invading microorganisms. The functions of neutrophils are significantly influenced by their cellular redox state and the production of ROS.

ROS are essential for the clearance of pathogens following phagocytosis. Upon migrating to inflammatory sites, neutrophils activate the superoxide‐generating enzyme, nicotinamide adenine dinucleotide phosphate (NADPH) oxidase, resulting in the generation of substantial amounts of ROS [42]. During this process, electrons are transferred from NADPH in the cytoplasm to molecular oxygen at the phagosomal membrane, leading to the initial formation of superoxide, which subsequently triggers the production of various other ROS. These reactive species are then converted into hydrogen peroxide (H_2_O_2_) through dismutation, effectively killing the engulfed pathogens [42, 43, 44].

ROS also play a critical role in the formation of NETs, which are structures secreted by activated neutrophils and consist of DNA fibers, histones, and antimicrobial proteins. NETosis is a dynamic cell death process in neutrophils that facilitates the formation of NETs. Within neutrophils, ROS are primarily produced through the NADPH oxidase (NOX) pathway or mitochondrial pathways, resulting in either NOX‐dependent or NOX‐independent NETosis.

In NOX‐dependent NETosis, various stimuli, including bacterial components, activate neutrophils. Research on neutrophils from patients with chronic granulomatous disease has shown that phorbol myristate acetate (PMA) activates protein kinase C (PKC), subsequently leading to NOX activation [45]. Once neutrophils are activated, the NOX complex produces ROS from molecular oxygen or generates derivative ROS in conjunction with myeloperoxidase (MPO) [46]. Following this activation, MAPKs such as extracellular ERK [47, 48], p38–MAPK [49], and c‐Jun N‐terminal kinase (JNK) [50] become activated. Ultimately, through transcriptional activation and chromatin decondensation, NETs are released into the extracellular space.

In cases of NOX‐independent NETosis, the primary driver for NET formation is the increase in mitochondrial ROS (mtROS) production. This mtROS generation is crucial for the subsequent steps in NET formation. The following step involves the activation of p38–MAPK [49], which plays a vital role in mediating cellular responses to stress and inflammation. Activated p38–MAPK subsequently influences the transcriptional mechanisms within neutrophils, enhancing transcriptional activation. An important aspect of calcium ion carrier‐triggered NOX‐independent NETosis is the elevation of intracellular calcium levels. The increase in calcium levels is associated with mtROS production, which depends on the opening of the mitochondrial permeability transition pore (mPTP), facilitating NETosis [48]. Furthermore, the activation of small conductance potassium channel member SK3 by calcium and mtROS can mediate the activation of NOX‐dependent NETosis [51]. In conclusion, similar to NOX‐dependent NETosis, NETs are released into the extracellular space.

##### 2.1.1.3 Dendritic Cells

DCs possess a distinctive capability to present exogenous antigens through a process known as cross‐presentation, which is crucial for eliciting immune responses against microbial infections and tumors. In cross‐presentation, antigens obtained from pathogen infections or tumor cells are internalized and processed by DCs, which then present these antigens to cytotoxic T lymphocytes during their maturation, thereby initiating a specific immune response. Both DC maturation and the cross‐presentation process are influenced by ROS.

ROS have a regulatory role in the maturation of DCs. For example, research has demonstrated that matrine impacts the maturity of DCs via the ROS/ERK/NF‐κB signaling pathway [52]. Additionally, ROS directly contribute to DC maturation by activating p38–MAPK and ERK1/2 [53], or they may impede maturation through the induction of endoplasmic reticulum (ER) stress [53]. Moreover, Nrf2 is identified as a critical regulator of DC maturation; specifically, the Kelch‐like ECH‐associated protein 1 (Keap1)/Nrf2 pathway may be involved in the maturation process induced by growth hormone [54]. Increased levels of ROS have been correlated with the enhanced maturation phenotype observed in *Nrf2−/−* immature DCs [55], while it has been reported that ROS levels bidirectionally regulate NF‐κB signaling based on cell type, with NF‐κB playing a significant role in the development, survival, and maturation of DCs [55].

Furthermore, ROS influence the capacity of DCs to cross‐present antigens to CD8+ T cells. When tumor cell‐derived microparticles (T‐MP) is treated as a pathogenic entity, DCs internalize the T‐MP into lysosomes, during which NOX2 catalyzes the production of ROS, elevating the lysosomal pH from 5.0 to a peak value of 8.5 [55]. This increase in ROS simultaneously activates the lysosomal calcium channel Mucolipin‐2, facilitating calcium release, which subsequently optimizes the cross‐presentation process [56]. ROS levels also play a role in the major histocompatibility complex I (MHC I)‐mediated presentation of processed antigens to CD8+ T cells; during this process, mitochondria regulate the cross‐presentation capabilities of plasmacytoid DCs (pDCs) in a ROS‐dependent manner. A notable reduction in mtROS production significantly diminishes the cross‐presentation ability of pDCs, consequently impairing their capacity to activate CD8+ T cell responses [57].

#### 2.1.2 Adaptive Immune System

In the context of adaptive immunity, the levels of ROS significantly influence the activation and metabolic processes of T cells and B cells, thereby impacting the progression of immune responses. This modulation underscores the critical role of ROS in shaping the functional dynamics of adaptive immune cells [58, 59]. However, despite these findings, a comprehensive understanding of the intricate mechanisms by which ROS affect T cell and B cell function remains to be fully elucidated, calling for further investigation into the interconnected pathways that govern immune response modulation.

##### 2.1.2.1 T Cell

T lymphocytes are the primary effector cells in cellular immunity, producing cytokines during immune responses to mediate inflammation and regulate other types of immune cells. In the immune system, the activation and metabolic processes of T cells are influenced by ROS.

During T cell activation, ROS act as second messengers that participate in regulating T cell signal transduction. Following the binding of the T cell receptor (TCR) to an antigen, a series of signaling events are triggered, including the production of ROS. These ROS can further activate downstream signaling pathways involving transcription factors (TFs) such as nuclear factor of activated T cells (NFAT) [60], activator protein 1 (AP‐1) [61, 62, 63], and NF‐κB [62, 63], thereby activating T cell activation, proliferation, and differentiation. mtROS are also associated with T cell activation. mtROS control T cell activation by modulating the expression of interleukin‐2 (IL‐2) and IL‐4, which is determined by oxidized signals from mitochondrial respiratory complex I [61]. Complex I of the mitochondrial electron transport chain (ETC) is a source of induced ROS formation, while mtROS derived from complex III are essential for CD4+ T cell activation and the expansion of antigen‐specific T cells [64].

GSH is a key regulator of T cell metabolism [65]. Activated T cells rely on GSH to control their increasing ROS levels. T cells lacking the catalytic subunit of γ‐glutamylcysteine ligase, which is necessary for GSH synthesis, exhibit limited NFAT activation and reduced targeting of mTOR, leading to a sharp decline in MYC expression [65, 66]. And GSH‐deficient T cells fail to expand, causing compromised pathogen clearance and dysregulated autoimmune responses. Furthermore, glutathione peroxidase 4 (GPX4) prevents ferroptotic death of antigen‐stimulated T cells by neutralizing lipid peroxides via GSH‐dependent reduction, blocking iron‐catalyzed peroxidation. In activated regulatory T (Treg) cells, GPX4 deficiency triggers lipid peroxide accumulation and ferroptosis upon TCR/CD28 stimulation, impairing immunosuppressive function while promoting proinflammatory cytokine release (e.g., IL‐1β) [67, 68, 69].

##### 2.1.2.2 B Cell

Similar to T cells, the activation and metabolism of B cells are also influenced by ROS.

B cell receptor (BCR) signaling regulates B cell activation and differentiation, with ROS acting as second messengers to modulate B cell signaling pathways. Although NOX exerts minimal direct regulation on B cell activation, the removal of ROS impairs BCR‐induced activation [70]. ROS enhance BCR signaling by reducing the activation threshold of spleen tyrosine kinase (Syk) through the inhibition of protein tyrosine phosphatases (PTPs) and participate in multiple signaling pathways [71, 72]. A deficiency in hydrogen voltage‐gated channel 1 results in decreased ROS production, which affects the activation of Syk and Akt, thereby weakening BCR signal transduction [73, 74].

ROS also influence the apoptosis of B cells. The excessive production of ROS triggers B cell apoptosis through a cascade involving cytochrome *c* and caspases [59, 75]. In addition, the activation of the JNK/p38–MAPK signaling pathway by ROS can also induce B cell apoptosis [76, 77], resulting in the translocation of Bcl‐2‐associated x protein (Bax) to the mitochondria, which initiates the disruption of the mitochondrial membrane potential and activates caspase‐9 and caspase‐3, and then intensifies B cell apoptosis [77].

#### 2.1.3 ROS Function in Host Defense

ROS play a crucial role in host defense, acting both as essential antimicrobial agents and, when in excess, as mediators of OS that can impair tissue repair. During the immune response to wounds and infections, professional phagocytes such as neutrophils and macrophages generate ROS through the NOX complex as part of the respiratory burst [78, 79, 80]. This enzymatic complex reduces molecular oxygen to O_2_•−, which is rapidly converted to H_2_O_2_ and other reactive species within phagosomes, effectively killing engulfed pathogens [78, 79, 80]. Moreover, H_2_O_2_ can diffuse extracellularly, creating antimicrobial gradients extending hundreds of micrometers from the wound site, further restricting bacterial growth and preventing infection spread [80].

However, ROS are not only microbicidal agents but also signaling molecules that regulate immune cell recruitment and inflammatory responses. For instance, quercetin decreased ROS‐induced OS and inflammation by suppressing NOX2 production [81].

ROS also mediate host defense through the activation of key inflammatory signaling pathways [82]. They modulate NLRP3 inflammasome activation by oxidizing mitochondrial thioredoxin and releasing thioredoxin‐interacting protein, facilitating inflammatory cytokine maturation and pyroptosis [83, 84]. ROS influence NF‐κB signaling by promoting IKKβ activation and facilitating nuclear translocation, thereby amplifying proinflammatory gene expression [85, 86]. MAPK pathways (including ERK, JNK, and p38) are activated via ROS‐dependent oxidation of upstream kinases, sustaining inflammatory responses [87, 88]. Additionally, ROS regulate Janus kinase/signal transducer and activator of transcription (JAK/STAT) signaling through modulation of STAT phosphorylation, affecting cytokine‐driven immune functions [89].

### 2.2 Redox Regulation of TFs

Numerous TFs function as downstream effectors within intracellular signaling pathways and are subject to redox regulation, thereby influencing signal transduction processes within the cell.

#### 2.2.1 Nuclear Factor Erythroid 2‐Related Factor 2

The Keap1–Nrf2–antioxidant response elements (ARE) pathway plays a critical role in maintaining cellular redox balance and metabolism, as well as in inducing adaptive responses to OS, thereby being closely associated with the pathogenesis of various diseases [90, 91]. When the levels of ROS increase in cells, they oxidize cysteine residues in Keap1, leading to the dissociation of Nrf2. This dissociation allows Nrf2 to escape ubiquitination and the subsequent proteasomal degradation, facilitating its translocation to the nucleus [92]. In the nucleus, Nrf2 forms heterodimers with Maf proteins to activate the transcription of antioxidant enzyme genes regulated by ARE, thereby preventing OS [93]. The Keap1–Nrf2–ARE pathway established by this process is pivotal in combating OS and maintaining cellular metabolic balance, and it is associated with adaptive responses in various inflammatory diseases. Furthermore, OS also influences the phosphorylation of Nrf2 and the nuclear export of Keap1 by activating other kinases, thereby further regulating Nrf2 activity and degradation, which finely tunes cellular responses to OS.

Multiple studies have revealed sophisticated mechanisms by which mtROS modulate the Keap1–Nrf2 pathway [94]. Increased mtROS induce modifications of Keap1 cysteine residues, disrupting this interaction and permitting Nrf2 activation [94]. And there is multiple proteins serving as a critical mediator linking mtROS levels to the regulation of the antioxidant TF Nrf2. For instance, mtROS promote the accumulation of phosphoglycerate mutase 5, which interacts with Keap1 and facilitates its translocation to the outer mitochondrial membrane. This interaction impairs Keap1‐mediated ubiquitination and proteasomal degradation of Nrf2, resulting in increased Nrf2 stability and enhanced transcriptional activity [95, 96]. Furthermore, mtROS influence selective mitophagy through an Nrf2‐dependent positive feedback loop involving p62/SQSTM1. By competing with Nrf2 for Keap1 binding, p62 sustains Nrf2 activation, integrating mtROS signaling with the autophagic removal of damaged mitochondria [97].

Furthermore, Nrf2's regulatory influence extends to ferroptosis. Activation of Nrf2 enhances cellular antioxidant defenses by upregulating genes such as GPX4 and FTH1, which reduce both ROS levels and free iron availability, thereby inhibiting ferroptosis [98, 99]. Nrf2 also modulates ferroptosis indirectly through noncoding RNAs, including lncRNAs and microRNAs (miRNAs), which influence Keap1 expression and Nrf2 nuclear translocation [100, 101, 102].

#### 2.2.2 Nuclear Factor Kappa‐B

There is a significant interaction between ROS and NF‐κB. As a crucial TF, NF‐κB is involved in various cellular processes, including immunity, inflammation, cell proliferation, and apoptosis, and is associated with numerous diseases. Both its classical and nonclassical activation pathways are strictly regulated, with the classical pathway primarily activating NF‐κB through IκB kinase β (IKKβ)‐mediated phosphorylation of inhibitors of NF‐κB α (IκBα), which leads to its degradation and allows NF‐κB to translocate to the nucleus to activate target gene transcription [103, 104]. Studies have shown that ROS mainly inhibit the phosphorylation of IκBα, thereby impairing its ubiquitination and degradation processes, including the influence of exogenous H_2_O_2_ on the phosphorylation of IκBα at tyrosine residues [103, 104]. Furthermore, ROS suppress IKK‐activating kinases while covalently inhibiting Ubc12's ubiquitin‐conjugating activity [104].

#### 2.2.3 Hypoxia‐Inducible Factor 1‐Alpha

ROS play a critical role in regulating the activity of HIF‐1α [105]. Under normal conditions, HIF‐1α is hydroxylated by its key inhibitor, prolyl hydroxylase 2 (PHD2), leading to its degradation. However, elevated levels of ROS can inactivate PHD2 through redox‐dependent dimerization, thereby stabilizing HIF‐1α even in the presence of sufficient oxygen [105]. Additionally, ROS can directly oxidize cysteine residues in the PHD protein, further inhibiting its activity, reducing HIF‐1α degradation, and reinforcing its stabilization. Meanwhile, the Cys520 residue in HIF‐1α itself can also be oxidized by ROS, thereby inhibiting the ubiquitination mediated by von Hippel–Lindau tumour suppressor protein, which further stabilizes HIF‐1α. These ROS‐mediated oxidative modifications result in the accumulation of HIF‐1α within the cell and promote its translocation to the nucleus [ 106]. In the nucleus, HIF‐1α forms a complex with HIF‐1β and is recruited to hypoxia response elements, thereby transactivating hypoxia‐responsive transcriptional programs, such as genes involved in immune response, energy metabolism, iron metabolism, glucose metabolism and transport, as well as cell proliferation, survival, and angiogenesis [105, 107, 108].

#### 2.2.4 FOXO

ROS have a complex regulatory effect on FOXO TFs, involving both direct redox control, such as the acetylation of FOXO mediated by acetyltransferase p300, which reduces its DNA‐binding capacity, and indirect pathways, such as the modulation of FOXO phosphorylation state and other posttranslational modifications via the influence of upstream factors like growth factor receptor activity, protein kinases, and phosphatases [109]. In the PI3K–protein kinase B (PKB) signaling pathway, ROS are generally associated with the activation of growth factor receptors, which suppresses FOXO activity through the activation of this pathway, promoting cellular metabolism and growth [110]. Additionally, while OS may lead to the inactivation of FOXO, it also enhances the activity of certain kinases, such as members of the MAPK family [111, 112, 113], which further regulate FOXO phosphorylation and function, thereby playing a crucial role in maintaining balance at the cellular and organismal levels.

### 2.3 Redox Control of Epigenetics

ROS exert bidirectional regulatory functions in epigenetic modifications. On one hand, excessive ROS can lead to DNA damage, gene mutations, and epigenetic changes, thereby promoting the occurrence and progression of tumors. On the other hand, moderate levels of ROS act as signaling molecules that can regulate the activity of epigenetic modification enzymes, subsequently influencing gene expression patterns.

ROS affect DNA methylation through various mechanisms. On one hand, they can directly influence the methylation landscape by causing oxidative DNA damage, such as the hydroxylation of pyrimidines and 5‐methylcytosine, interfering with the epigenetic signals associated with 5‐hydroxymethylcytosine [114]. On the other hand, ROS can also impact the demethylation state of DNA through oxidative DNA modifications and ten‐eleven translocation enzyme‐mediated hydroxymethylation processes [115]. Furthermore, ROS can indirectly regulate the activity of epigenetic mechanisms, as the activity of histone modifying enzymes depends on the levels of intracellular metabolites, such as acetyl‐CoA, Fe, α‐ketoglutarate, nicotinamide adenine dinucleotide (NAD), and S‐adenosylmethionine [116].

In the context of tumor development, ROS can exert effects through epigenetic mechanisms. ROS‐induced DNA damage, such as the formation of 8‐hydroxy‐2′‐deoxyguanosine (8‐OHdG), can perturb DNA methyltransferase (DNMT)‐substrate recognition by interfering with the ability of DNA to serve as a substrate for DNMTs, potentially promoting malignancy [117, 118]. Moreover, ROS can further disrupt DNA methylation patterns and transcriptional activation processes by affecting the binding affinity of methyl‐binding proteins [119]. Additionally, studies have shown that the downregulation of key antioxidant enzyme expression is associated with hypermethylation of promoters. For example, decreased expression of manganese SOD (MnSOD) [120], metallothionein [121], and NAD(P)H: quinone oxidoreductase 1 (NQO1) [122] is closely linked to the incidence and progression of various cancers. The silencing of these antioxidant enzymes may be mediated by promoter‐specific DNA hypermethylation events, further confirming the crucial role of ROS‐induced epigenetic alterations in tumor development.

Furthermore, the cellular redox state can also indirectly impact the methylation status of DNA and histones by affecting the activity of epigenetic modification enzymes or the supply of metabolites. Metabolic intermediates provided by the mitochondrial tricarboxylic acid (TCA) cycle serve as substrates and cofactors for epigenetic modification enzymes, playing a critical role in regulating epigenetic modifications [123, 124]. ROS may also induce locus‐specific hypermethylation by upregulating DNMT expression or forming new complexes containing DNMT, further influencing cellular epigenetics [125].

### 2.4 Redox Regulation of Metabolism and Bioenergetics

**FIGURE 2:**
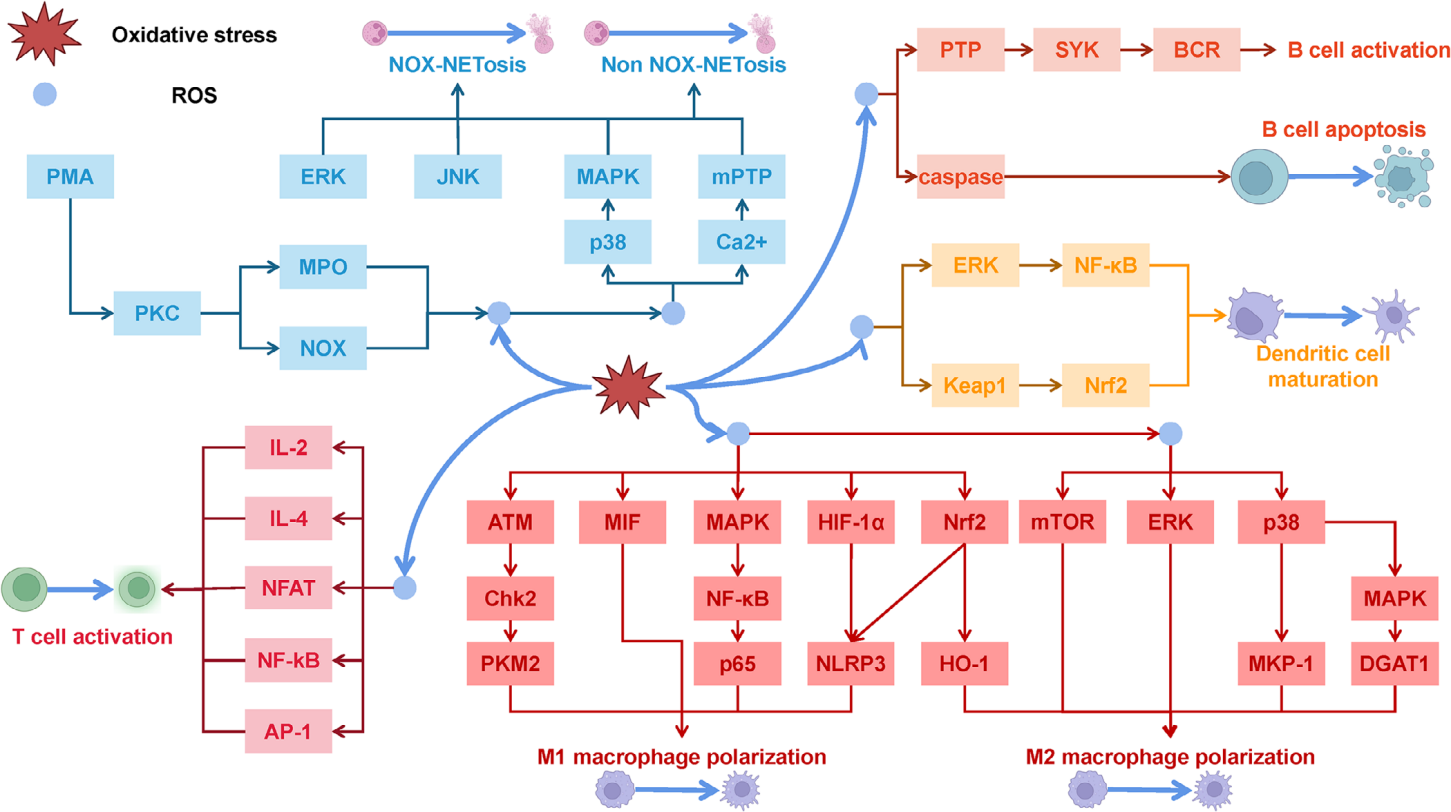
Redox regulation of metabolism and bioenergetics. Mitochondria are essential for aerobic respiration and ATP synthesis through oxidative phosphorylation, facilitated by the ETC, which consists of four core complexes (I, II, III, and IV) and coenzyme Q (CoQ). Electrons from nicotinamide adenine dinucleotide [reduced form] (NADH) and flavine adenine dinucleotide, reduced (FADH_2_) are sequentially transferred through these complexes, ultimately reducing oxygen to form water; however, electron leakage at complex I and III can lead to the generation of ROS such as O_2_•− and H_2_O_2_. ROS produced by complex III can stabilize the transcription factor HIF1α, enhancing cellular adaptation to hypoxic conditions. The intracellular antioxidant system, comprising enzymatic antioxidants such as SOD, CAT, plays a critical role in mitigating excess ROS and maintaining redox balance. Additionally, the activity of ETC complexes and the regulation of mitochondrial dynamics through fission and fusion events are pivotal in modulating ROS production. Beyond their role in ROS generation, these reactive species are vital in regulating mitochondrial energy metabolism by activating or inhibiting pathways, such as AMPK and various metabolic processes, thereby influencing the tricarboxylic acid cycle (TCA cycle), fatty acid β‐oxidation, and mitochondrial biogenesis. Together, these mechanisms are crucial for maintaining mitochondrial function and optimizing cellular energy homeostasis. This figure was created with BioRender (https://biorender.com/).

OS serves as a critical modulator of mitochondrial bioenergetics, dynamically influencing cellular metabolic homeostasis through its regulation of redox‐sensitive pathways in the ETC and TCA cycle (Figure 2).

#### 2.4.1 Redox Regulation of mtROS Production

Mitochondria are the primary sites for aerobic respiration in cells, synthesizing ATP through the process of oxidative phosphorylation. The ETC consists of four core complexes (i.e., complex I, II, III, and IV) and coenzyme Q (CoQ), which work together to form a coherent electron transport system [126, 127] that catalyzes chemiosmotic ATP biosynthesis. In this system, electrons originate from nicotinamide adenine dinucleotide [reduced form] (NADH) or flavine adenine dinucleotide, reduced (FADH_2_) and are gradually transferred through these complexes, ultimately being transferred to oxygen, leading to the generation of water [126, 127]. However, there is a phenomenon of electron leakage within the ETC, which typically occurs at specific sites in complex I and III, resulting in the combination of electrons with oxygen to produce superoxide anion radicals (O_2_•−) and other ROS such as H_2_O_2_ and hydroxyl radicals (•OH) [127].

The regulation of the intracellular antioxidant system involves the synergistic action of various antioxidant enzymes and nonenzymatic antioxidants, which effectively eliminate ROS and prevent their excessive accumulation. SOD, as a crucial enzyme, converts O_2_•− into H_2_O_2_, which is subsequently decomposed into water and nontoxic metabolites by CAT and GSH peroxidase (GSH‐Px), thereby maintaining redox balance within the cell [128, 129, 130]. In addition, the active states of ETC complexes are finely regulated through posttranslational modification mechanisms such as phosphorylation, acetylation, and nitration, which in turn affect ROS production. For example, the phosphorylation state of ETC complex I is directly correlated with its activity and ROS generation levels [131]. Furthermore, the regulation of mitochondrial dynamics, including the processes of mitochondrial fission and fusion, significantly impacts ROS generation. Mitochondrial fission promotes ROS production by increasing the surface area of the ETC, while mitochondrial fusion aids in ROS clearance and mitochondrial function restoration, thereby enabling a complex regulation of ROS production at the cellular level [132].

#### 2.4.2 Redox Regulation of Energy Metabolism

ROS play a crucial role in regulating mitochondrial energy metabolism. As signaling molecules, ROS can activate or inhibit specific signaling pathways, thereby modulating mitochondrial metabolic pathways. For example, ROS can activate AMPK, which is a key energy sensor that regulates cellular energy metabolism [133]. Additionally, ROS can influence various metabolic pathways within mitochondria, including the TCA cycle, fatty acid β‐oxidation, and amino acid metabolism. The regulation of these pathways alters the generation and consumption of metabolic products, significantly impacting the energy status of the cell [7]. Moreover, ROS are involved in the regulation of mitochondrial biogenesis processes, such as the replication, transcription, and translation of mitochondrial DNA (mtDNA), as well as mitochondrial dynamics, including mitochondrial fission and fusion. These processes are vital for maintaining normal mitochondrial function and fine‐tuning energy metabolism [7].

### 2.5 Redox Regulation of Proteostasis

**FIGURE 3:**
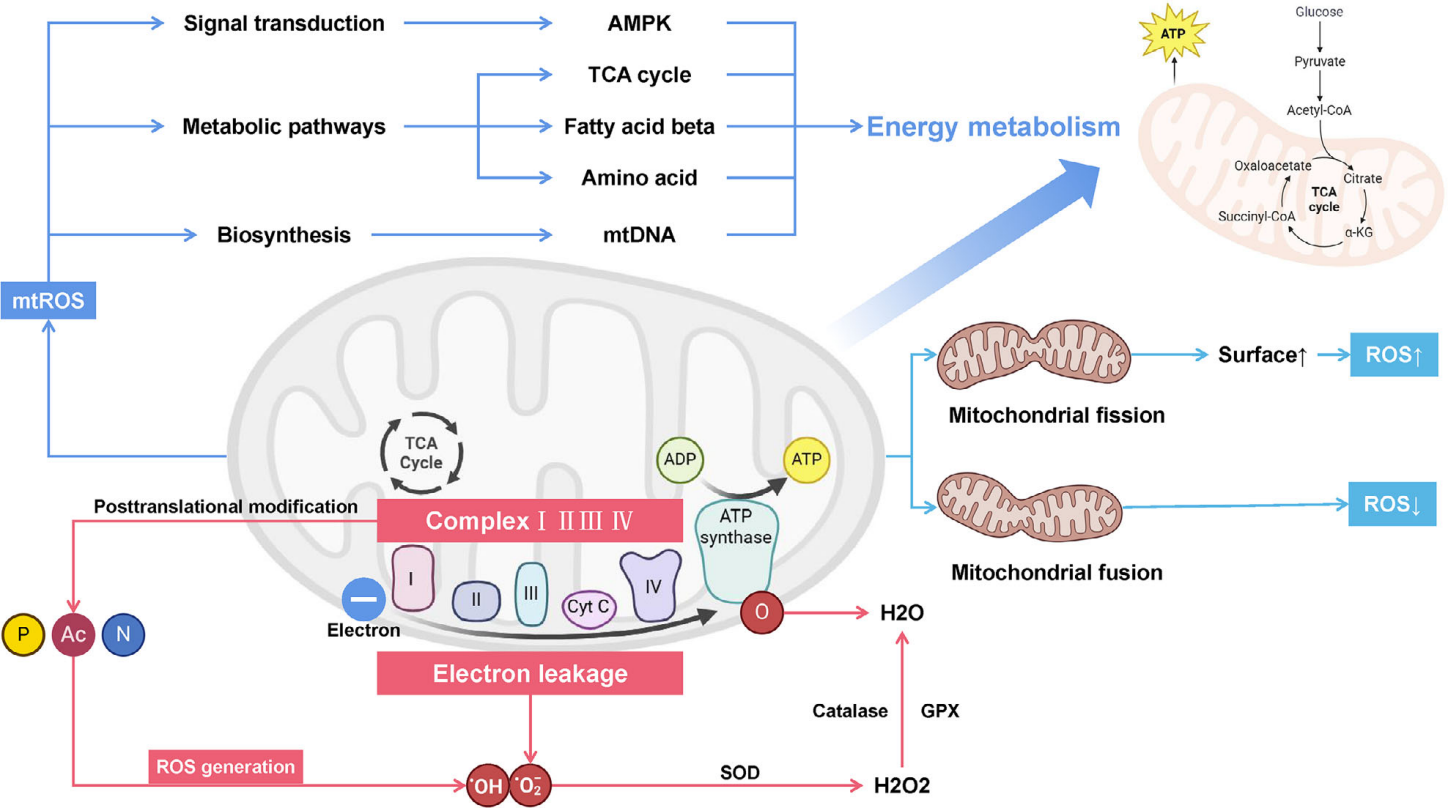
Redox regulation of proteostasis. In mRNA translation, the redox state modulates the activity of initiation factors, such as eukaryotic translation initiation factor 4E (eIF4E) and eukaryotic translation initiation factor 2 (eIF2), by influencing their phosphorylation and GTPase activities, respectively. Additionally, ROS impact translation termination through their effects on release factors like eukaryotic release factor 1 (eRF1) and eukaryotic release factor 1 (eRF3), while also influencing ribosome biogenesis. Within the ER, ROS are essential for maintaining the oxidative environment required for protein folding and disulfide bond formation, facilitated by enzymes like protein disulfide isomerase (PDI) and ERO1‐like alpha protein (ERO1A). However, excess ROS disrupt ER redox homeostasis, leading to misfolded proteins and triggering the unfolded protein response (UPR), which can further increase ROS levels. In protein degradation, ROS enhance the removal of misfolded proteins through the ubiquitin–proteasome system and autophagy. Under oxidative stress and heat shock 70 kDa protein (Hsp70), the 26S proteasome dissociates into 20S and 19S subunits, and S‐glutathionylation increases the proteolytic activity of the 20S proteasome. Furthermore, in autophagy, ROS activate AMP‐activated protein kinase (AMPK), influencing UNC51‐like kinase 1 (ULK1 kinase) and initiating autophagosome formation, while also directly affecting the activity of autophagy‐related 4 (ATG4) and so on, thereby orchestrating critical processes for protein homeostasis and cellular function such as cleavage and lipidation of microtubule‐associated protein 1A/1B‐light chain 3 (LC3)/ATG8. This figure was created with BioRender (https://biorender.com/).

Protein homeostasis is a crucial guarantee for normal cellular physiological functions, and its maintenance relies on complex regulatory mechanisms. Among these mechanisms, redox regulation plays a significant role, markedly influencing the synthesis, folding, and degradation of proteins within the cell (Figure 3).

#### 2.5.1 Redox Regulation of mRNA Translation

The entire process of mRNA translation is precisely regulated by the redox state. Studies have shown that the activity of initiation factors during translation is modulated by the redox state. For example, the phosphorylation state of eukaryotic translation initiation factor 4E (eIF4E) can influence its ability to bind to the 5′ cap structure of mRNA, and this phosphorylation process may be regulated by ROS [134, 135]. Furthermore, the GTPase activity of eukaryotic translation initiation factor 2 (eIF2) is also regulated by redox‐sensitive modifications, thereby affecting the efficiency of translation initiation [135, 136, 137, 138]. Translation termination involves the recognition of stop codons and the release of nascent proteins, and this process is similarly regulated by the redox state. For instance, the activities of release factors eukaryotic release factor 1 (eRF1) and eRF3 may be modulated by ROS, affecting the efficiency of translation termination [135]. Research indicates that H_2_O_2_ may directly impact ribosome biogenesis and the enrichment of proteins in cytoplasmic translation, thereby regulating the activity of ribosomal proteins and translation initiation factors [138].

#### 2.5.2 Redox Regulation of ER Homeostasis

ER is a core site for protein folding and modification within the cell, crucial for maintaining physiological functions. Within the ER, membrane proteins and secretory proteins undergo precise folding, especially the formation of disulfide bonds, a process reliant on the oxidative environment of the ER, termed “oxidative protein folding.” Protein disulfide isomerase (PDI) and the oxidoreductase ERO1‐like alpha protein (ERO1A) serve as key enzymes, working together to maintain the oxidative environment of the ER and induce disulfide bond formation [139, 140]. The active site of PDI effectively captures electrons from free thiols of nascent proteins, facilitating the precise construction of disulfide bonds [139, 140]. Accumulation of ROS can disrupt the redox homeostasis of the ER, leading to the accumulation of misfolded proteins and ER stress, which ultimately activates the unfolded protein response (UPR) [141, 142]. Conversely, the UPR can lead to the formation of additional ROS and affect mitochondrial function [141, 142].

#### 2.5.3 Redox Regulation of Protein Degradation

Under conditions of OS, cells enhance the removal of misfolded and/or oxidized proteins through a series of finely tuned regulatory mechanisms such as the ubiquitin–proteasome system and autophagy. The 26S proteasome is responsible for degrading ubiquitin‐tagged proteins under nonstress conditions. However, during OS and low ATP levels, it splits into the 20S and 19S subunits, a process supported by heat shock 70 kDa protein (Hsp70) [143]. S‐glutathionylation, as a redox regulatory mechanism, can directly act on the 20S proteasome, opening its gate structure and thus enhancing its proteolytic activity [144]. Additionally, the ubiquitin–proteasome system regulates cellular redox balance through the degradation of Nrf2 and the activation of NF‐κB, both of which can mediate ROS levels via their downstream antioxidant proteins [145]。

In the regulation of autophagy, the redox state plays a crucial role. Specifically, ROS produced by mitochondria can activate AMPK by oxidizing cysteine residues within the enzyme, leading to the phosphorylation of UNC51‐like kinase 1 (ULK1) kinase, which inactivates it; the inactivation of ULK1 is a key step in the formation of autophagosomes [146]. Moreover, the p62/Keap1/Nrf2 system reveals a collaborative mechanism between autophagy and redox regulation, where p62 activates Nrf2 in a redox‐independent manner by regulating Keap1 degradation, promoting Nrf2 nuclear translocation and inducing antioxidant gene transcription [146]. Multiple key components in the autophagy pathways are also directly regulated by the redox state. The protease autophagy‐related 4 (ATG4), which regulates microtubule‐associated protein 1A/1B‐light chain 3 (LC3)/ATG8 cleavage and lipidation, contains catalytic and redox‐sensitive regulatory cysteine residues [147]. The oxidation state of these cysteine residues can affect the activity of ATG4, thereby regulating the cleavage and lipidation of LC3/ATG8, critical steps in autophagosome formation and maturation [147]. Additionally, ATG3 and ATG7, two enzymes containing catalytic cysteine, undergo inhibition under oxidative conditions, which affects the progression of the autophagy process [148].

### 2.6 Roles of ROS in Wound Healing

ROS play critical and multifaceted physiological roles in the wound healing process. ROS function not only as antimicrobial agents but also as essential signaling molecules that regulate and promote various key stages of tissue repair.

First, ROS notably stimulate the proliferation and migration of pivotal cell types involved in repair, including fibroblasts, keratinocytes (KCs), and smooth muscle cells. H_2_O_2_ induces the expression of matrix metalloproteinases (MMPs) such as MMP‐1 and MMP‐2 at low concentrations, facilitating cell migration through extracellular matrix (ECM) remodeling—a fundamental requirement for cell movement and new tissue formation [149, 150, 151]. In fibroblasts, ROS‐mediated AP‐1‐dependent MMP‐1 transcription and JNK pathway activation further exemplify their regulatory role in cellular migration and tissue remodeling [149, 152, 153].

Second, ROS play a pivotal physiological role in angiogenesis. Vascular endothelial growth factor (VEGF) stimulation in ECs triggers a biphasic ROS production involving NOX enzymes and mitochondria [154, 155, 156]. Initially, VEGF rapidly induces NOX4‐derived H_2_O_2_ in the cytosol, which subsequently activates NOX2, leading to sustained mtROS generation [157]. This ROS‐induced ROS release amplifies and sustains VEGF receptor‐2 signaling, promoting endothelial proliferation, migration, and new blood vessel formation [157]. Mechanistically, NOX4 activation may involve rapid tyrosine phosphorylation facilitated by adaptor proteins such as Grb2 within multiprotein complexes, similar to mechanisms observed with insulin‐like growth factor 1 [158]. NOX4‐derived H_2_O_2_ further activates NOX2 through phosphorylation of regulatory subunits like p47phox [159, 160] and Rac1 [161].

ROS also modulate the activity of diverse growth factors and their receptors, which are instrumental in wound repair. H_2_O_2_ promotes the activation of platelet‐derived growth factor (PDGF) signaling and triggers epidermal growth factor receptor phosphorylation, driving proliferation and migration of KCs [162, 163].

## 3 Overview of OS

**FIGURE 4:**
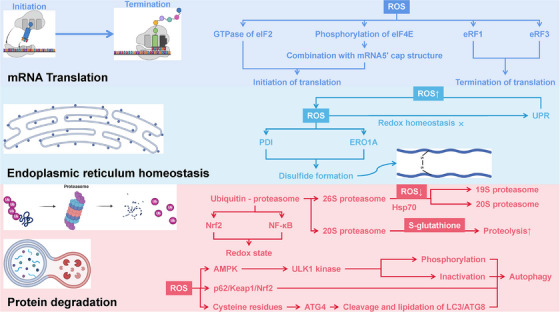
Overview of oxidative stress. Lipid peroxides serve multiple functions in cellular physiology, including the inhibition of autophagy via the mammalian target of rapamycin (mTOR), pathway and the promotion of selective autophagy to eliminate damaged organelles. During apoptosis, peroxidized fatty acids enhance the permeability of the mitochondrial outer membrane, leading to the release of cytochrome *c* and subsequent cell death. Additionally, lipid peroxides are involved in ferroptosis and act as danger signals that activate proinflammatory pathways. In the immune response, lipid peroxides bind to immune cell receptors such as Toll‐like receptor 4 (TLR4) to activate the NF‐κB signaling pathway, enhance the inflammatory response, and promote recruitment and activation of macrophages and T cells. Protein oxidation occurs primarily due to ROS and other oxidants, resulting in the oxidative modification of specific amino acid residues, which disrupt protein functions and contribute to cellular signaling and metabolic dysfunction. This oxidative damage modifies protein–protein interactions and can impair cellular pathways. In terms of DNA, oxidative damage primarily leads to base modifications and strand breaks, with notable products like 8‐OHdG exhibiting high mutagenic potential. Cells respond to oxidative DNA damage by activating repair mechanisms, including base excision repair, which involves a coordinated effort of specific repair enzymes. The accumulation of oxidative mutations has significant implications for tumorigenesis, while oxidative damage is also a contributing factor in neurodegenerative diseases. Mitochondria, as the energy centers of the cell, produce ATP through the ETC, but oxidative stress can lead to excessive ROS production, causing mitochondrial dysfunction and increased cell death. This figure was created with BioRender (https://biorender.com/).

OS emerges as a systemic imbalance between ROS generation and antioxidant buffering capacity, manifesting through four interconnected pathological axes: LPO, protein oxidation, DNA oxidative damage, and mitochondrial bioenergetic dysfunction (Figure 4).

### 3.1 Lipid Peroxidation

LPO is primarily initiated by ROS. In this process, ROS react with polyunsaturated fatty acids (PUFAs) to form lipid radicals, such as acrylate radicals (R•), which subsequently generate various peroxidized fatty acids (ROOH) and aldehydes, including 4‐hydroxy‐2‐alkenal and acrolein, through chain reactions [164, 165]. Lipid peroxides play multiple roles in cellular physiology. For example, they can act as autophagy signals by activating the mTOR pathway to inhibit autophagy, or they may induce selective autophagy under specific conditions to eliminate damaged organelles [166, 167, 168, 169]. Additionally, they can initiate the autophagy process through the JNK‐Bcl‐2/Beclin1 signaling pathway [167]. During apoptosis, peroxidized fatty acids can activate apoptotic signaling pathways, promoting the activation of Bax and Bak proteins, which increases the permeability of the mitochondrial outer membrane, triggering the release of cytochrome *c* and ultimately leading to cell death [167]. ROS can also modulate apoptosis through pathways such as NF‐κB [167, 170, 171].

Furthermore, lipid peroxides play a critical role in ferroptosis, where iron‐catalyzed free radical generation accelerates the accumulation of lipid peroxides, resulting in nonapoptotic cell death [167, 172]. In the immune system, these peroxides act as danger signals, activating the NF‐κB signaling pathway upon binding to immune cell receptors (e.g., Toll‐like receptor 4 [TLR4] [173]), which enhances the inflammatory response and promotes the recruitment and activation of macrophages and T cells [167].

However, abnormal LPO is closely associated with various diseases. In neurodegenerative diseases such as Alzheimer's disease (AD), peroxidation products accelerate the aggregation of amyloid proteins and may lead to neuronal death by disrupting cellular signaling [172, 174, 175]. In the development of tumors and cancer, LPO products promote the proliferation and metastasis of cancer cells, influencing crucial signaling pathways such as MAPK and PI3K/Akt [176].

### 3.2 Protein Oxidation

Protein oxidation is a process induced by ROS, nitric oxide (NO), and other oxidants, primarily leading to the oxidative modification of certain amino acid residues in proteins (such as cysteine, lysine, and tyrosine). These oxidative modifications can significantly alter the physical and chemical properties of proteins, leading to conformational changes, functional loss, and aggregation. Oxidized proteins typically form carbonyl derivatives, sulfonyl derivatives, and disulfide bonds, which may interfere with their normal functions. In cellular physiology, oxidatively damaged proteins undergo a series of processing steps. First, these damaged proteins are marked for degradation through ubiquitination, allowing them to be recognized and transferred into autophagosomes for degradation, thereby maintaining intracellular homeostasis. Moreover, oxidized proteins can affect signaling pathways by altering protein–protein interactions, such as influencing the activity of key signaling pathways like MAPK and PI3K/Akt.

During apoptosis, oxidized proteins serve as regulatory factors. The tumor suppressor protein p53 exhibits increased stability and activity upon oxidative damage to DNA or proteins, inducing cell cycle arrest, inhibiting cell proliferation, and potentially leading to apoptosis [177, 178]. The Bax protein is responsible for promoting changes in mitochondrial membrane permeability during apoptosis, facilitating the release of cytochrome *c*, which in turn activates the caspase cascade and results in cell death [177, 179, 180]. Oxidized proteins also play a significant role in immune responses; for example, oxidized low‐density lipoprotein (oxLDL) can be taken up by macrophages, transforming them into foam cells, a key process in the development of atherosclerosis (AS) [181, 182, 183].

Protein oxidation is closely associated with the onset of various human diseases. In neurodegenerative diseases like AD, the accumulation of oxidatively modified tau protein and β‐amyloid protein forms neurofibrillary tangles and plaques, leading to neuronal death and functional impairment [184]. Additionally, in type 2 diabetes, oxidative damage from glucose may impair insulin receptor tyrosine kinase activity by altering the phosphorylation states of downstream signaling molecules such as insulin receptors and insulin receptor substrates (IRS‐1), leading to insulin resistance [185].

### 3.3 DNA Oxidative Damage

DNA oxidative damage is characterized by injuries caused by ROS and other oxidants, primarily involving modifications to DNA bases and strand breaks [186, 187]. Common oxidative damage products include 8‐OHdG, a modification resulting from the oxidation of guanosine in DNA, which exhibits a high mutagenic potential [186, 187].

Following oxidative damage, cells activate their DNA repair mechanisms, with base excision repair being the most prevalent pathway. This process involves specific repair enzymes such as DNA glycosylases, DNA polymerases, and ligases, which collaboratively recognize, repair, and replace damaged nucleotides. Additionally, oxidatively damaged DNA can activate checkpoint kinases such as ATM, initiating cell cycle responses [186, 188].

In the context of cancer, the relationship between DNA oxidative damage and tumorigenesis is particularly significant [189]. The accumulation of mutations can activate oncogenes or inactivate tumor suppressor genes, such as the loss of p53 function, promoting unlimited cell proliferation [190, 191]. DNA oxidative damage is also closely linked to various neurodegenerative diseases; in AD, the accumulation of oxidative damage is considered a contributing factor to neuronal death [192, 193, 194].

### 3.4 Mitochondrial Respiration and Metabolism

Mitochondria are the energy centers of the cell and are also the primary sites for ROS generation. In the mitochondrial ETC, ATP synthesis primarily relies on the effective operation of complexes I–IV [126, 127]. Under normal conditions, mitochondria provide ATP to cells through oxidative phosphorylation. However, under conditions of OS, excessive ROS can have a destructive impact on mitochondria, directly affecting ATP synthesis and the stability of cellular metabolism [7, 133].

In mitochondria, excessive ROS generation usually leads to damage to the mitochondrial membrane, increasing membrane permeability and inducing the release of proapoptotic factors such as cytochrome *c*. This process activates apoptotic signaling pathways and further triggers the caspase cascade, resulting in cell death [195, 196, 197]. Additionally, OS may compromise the integrity of mtDNA, leading to mutations in the mitochondrial genome and diminished function, which holds significant pathological relevance in many chronic diseases [198].

Mitochondrial dysfunction is recognized as one of the essential mechanisms underlying various disease states. In patients with metabolic syndrome and type 2 diabetes, mitochondrial dysfunction is often accompanied by abnormalities in energy metabolism, resulting in hepatic fat infiltration and insulin resistance [199, 200, 201, 202]. Furthermore, mitochondrial dysfunction is closely associated with cardiovascular diseases; studies indicate that declines in mitochondrial function in heart disease patients directly lead to the apoptosis of cardiac myocytes and a reduction in cardiac function [203, 204, 205]. In neurodegenerative diseases, OS from mitochondrial damage induces an imbalance in neuronal energy metabolism, increasing β‐amyloid accumulation and accelerating the progression of neurodegenerative diseases such as AD [206, 207, 208].

## 4 Role of OS in Human Disease

**FIGURE 5:**
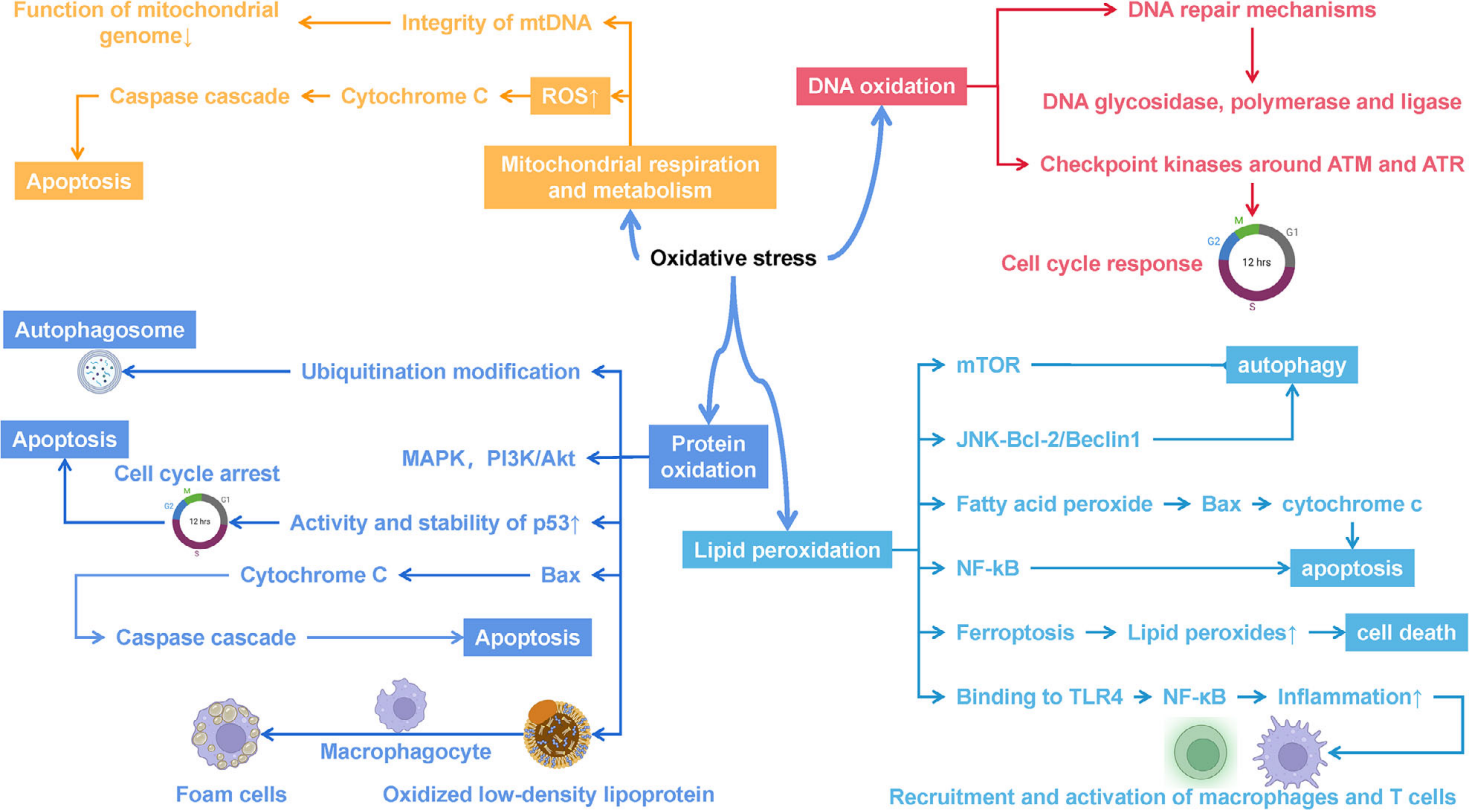
Overview of oxidative stress in human disease. In neurodegenerative conditions, oxidative stress is implicated in the pathological processes of Alzheimer's disease, Parkinson's disease, and Huntington's disease, as well as in brain injuries and chronic pain syndromes. In the context of cancer, oxidative stress primarily influences the epithelial–mesenchymal transition, tumor growth, metastasis, and chemoresistance mechanisms. Within the circulatory system, it contributes to cardiovascular diseases, including heart failure, atherosclerosis, aortic dissection, heart transplantation, and myocardial infarction. In liver pathology, oxidative stress impacts hepatotoxicity, alcoholic liver disease, liver fibrosis, and cirrhosis. Regarding kidney disease, it affects nephrotoxicity, diabetic nephropathy, acute kidney injury, and chronic kidney disease. In metabolic disorders, oxidative stress is implicated in the development of diabetes mellitus, obesity, hyperlipidemia, nonalcoholic fatty liver disease, and fatty liver disease. In autoimmune conditions, oxidative stress plays a crucial role in the pathogenesis of systemic lupus erythematosus, rheumatoid arthritis, psoriasis, and other immune‐mediated diseases. In musculoskeletal disorders, it is associated with conditions such as osteoarthritis, osteoporosis, and sarcopenia, leading to functional declines in skeletal muscle and joint health. Furthermore, in retinal diseases, oxidative stress influences glaucoma, diabetic retinopathy, and age‐related macular degeneration. Last, in reproductive system disorders, it affects both male and female infertility. This figure was created with BioRender (https://biorender.com/).

OS is increasingly recognized as a fundamental driver of disease pathology across multiple organ systems (Figure 5). Recent advances have deepened our understanding of its role in disrupting cellular homeostasis, modulating inflammatory and metabolic pathways, and contributing to disease progression. The intricate interplay between OS and various molecular networks has unveiled novel insights into its systemic impact, providing new perspectives on disease mechanisms and therapeutic strategies. In the following sections, we explore emerging breakthroughs in elucidating the significance of OS in human health and disease, with a focus on its implications in both disease progression and preclinical therapeutic interventions.

### 4.1 Nervous System Diseases

#### 4.1.1 Alzheimer's Disease

AD is a prevalent neurodegenerative disorder characterized by progressive cognitive dysfunction and behavioral changes, with OS recognized as key factors in the pathogenesis of AD. In the brain tissue of AD patients, the level of OS is significantly elevated, and this phenomenon is closely associated with degenerative changes in neurons and declines in cognitive abilities. The increase in ROS in AD patients primarily arises from multiple factors, including mitochondrial dysfunction, the aggregation of amyloid‐beta (Aβ), and neuroinflammation.

In AD, the accumulation of Aβ has a pronounced impact on cellular metabolism and survival, particularly regarding the dynamic balance of mitochondrial function and OS [209]. The accumulation of Aβ compromises the integrity of the mitochondrial membrane, leading to a reduction in mitochondrial inner membrane potential. This process not only activates the mitochondrial permeability transition but also enhances the membrane potential, thereby exacerbating the production of ROS [206, 210]. The accumulated ROS can directly damage mitochondria, resulting in functional impairments and triggering a series of apoptotic pathways involving cytochrome *c* [206]. Additionally, ROS can activate various cellular stress kinase signaling pathways, such as JNK [211] and p38–MAPK [212], through the damaging of intracellular lipids, proteins, and DNA, thus promoting apoptosis and accelerating the progression of AD [206].

Moreover, OS is closely linked to the immune responses of microglia and astrocytes in AD. Microglia, acting as immune sentinels in the brain, are typically activated as a protective response. However, during the pathological process of AD, overactive microglia release large amounts of proinflammatory factors such as tumor necrosis factor‐α (TNF‐α), IL‐1β, and IL‐6 [192]. These proinflammatory factors activate signaling pathways like NF‐κB and the NLRP3 inflammasome, leading to further inflammatory responses and creating a vicious cycle of interaction [192, 213]. This neuroinflammation may result in neurons being unable to effectively counter oxidative damage, which is a significant contributor to cognitive decline in AD patients [192, 213].

In AD, reactive nitrogen species (RNS) also contributes to neurodegeneration primarily through nitrative and nitrosative modifications of key proteins involved in disease pathology [192, 213]. RNS‐mediated S‐nitrosylation of tau promotes tau hyperphosphorylation, aggregation, and Aβ oligomerization, accelerating plaque and tangle formation [214, 215, 216]. Peroxynitrite (ONOO⁻)‐induced tyrosine nitration further destabilizes tau and amyloid precursor protein, exacerbating protein misfolding and synaptic dysfunction [214, 215, 217, 218].

RNS also impair neurotrophic signaling by nitrating proNGF, reducing its binding to TrkA and downregulating TrkA expression, thereby shifting signaling toward p75NTR‐mediated apoptosis [214]. Concurrently, nitrative damage to motor proteins and microtubule‐associated tau disrupts axonal transport of survival signals, especially affecting basal forebrain cholinergic neurons [214, 215]. Additionally, NO‐related pathways dysregulation, including impaired NO/cGMP signaling, contributes to synaptic failure and cognitive deficits [215, 219, 220]. The interplay of RNS‐induced protein modifications, neurotrophic receptor imbalance, and transport deficits underlies a pathogenic cascade linking oxidative/nitrative stress with neuronal loss and dysfunction in AD [215].

In the context of AD, the dysregulation of iron homeostasis is also closely related to heightened OS. Existing studies have indicated a significant increase in iron levels in the brains of AD patients. This excess iron can accelerate ROS production via the Fenton reaction, leading to LPO and cell death, and may also activate ferroptosis, a newly recognized mechanism of cell death [221, 222]. Iron promotes the accumulation of lipid peroxides and inhibits GPX4 activity by reacting with intracellular ROS, thereby enhancing cellular oxidative damage [221, 222, 223, 224]. Furthermore, excessive iron accumulation damages mitochondria and interferes with mitochondrial energy metabolism, further exacerbating oxidative damage [221, 222, 225]. Therefore, regulating iron homeostasis in the brain has become a crucial research direction for AD therapy, with the development of drugs aimed at reducing iron accumulation and inhibiting OS being a current focus of preclinical studies.

#### 4.1.2 Parkinson's Disease

Parkinson's disease (PD) is a progressive neurodegenerative disorder characterized by various motor impairments including muscle rigidity and resting tremor. OS emerges as a central pathogenic mechanism in PD, intricately linked with multiple pathological hallmarks. In the PD brain, heightened OS correlates with dopaminergic neuron loss, α‐synuclein (α‐syn) aggregation, aberrant iron deposition, and neuroinflammatory activation.

Iron dysregulation plays a pivotal role in PD progression through OS induction and ferroptosis association [226]. Excessive iron catalyzes free radical generation via Fenton reactions, exacerbating cellular damage [226, 227]. Dopaminergic neurons exhibit upregulated transferrin and transferrin receptor (TFR) expression, promoting iron overload that triggers proinflammatory cytokine release and accelerates neuronal injury [226, 227, 228]. Critical iron accumulation induces ferroptosis—an iron‐dependent cell death pathway. In PD substantia nigra, iron deposition stimulates ROS production and initiates irreversible ferroptotic processes through disrupted iron homeostasis, enhanced LPO, and mitochondrial dysfunction [226, 229].

OS concurrently drives α‐syn pathological aggregation. Aggregated α‐syn compromises membrane integrity, alters permeability, and induces ROS generation [230]. Membrane‐bound α‐syn aggregates facilitate abnormal calcium influx, activating ROS‐producing signaling cascades [230]. These changes deplete endogenous GSH reserves, increasing neuronal vulnerability to oxidative damage. This self‐perpetuating cycle disrupts chaperone‐mediated autophagy and accelerates neuronal destruction [230, 231].

Dopamine (DA) serves dual roles as a neurotransmitter and antioxidant modulator. It stabilizes GPX4 to inhibit iron‐mediated ROS production, maintaining redox homeostasis [232]. However, DA depletion in PD patients weakens antioxidant defenses, increasing neuronal susceptibility to oxidative membrane damage and apoptosis. The DA transporter (DAT) critically regulates synaptic DA levels and neuronal protection [233]. DAT‐mediated DA reuptake prevents oxidative toxicity from synaptic DA excess while supporting intracellular GSH maintenance for ROS neutralization [233, 234, 235]. PD‐associated DAT downregulation causes synaptic DA accumulation, exacerbating OS and creating a pathogenic loop that promotes ROS accumulation and neuronal death [233, 235].

Mitochondrial dysfunction significantly amplifies OS in PD. Genetic mutations (e.g., Parkin) impair mitophagy, permitting damaged mitochondria to accumulate ROS [231]. Compromised mitochondrial function reduces ATP synthesis and disrupts membrane integrity, worsening oxidative environments [231]. Enhanced calcium influx through mitochondrial l‐type channels accelerates basal OS in substantia nigra dopaminergic neurons via increased DA metabolism, promoting age‐related mitochondrial oxidative damage and cell death in PD pathogenesis [236, 237].

In PD, RNS contribute to dopaminergic neurodegeneration primarily through nitrative damage to mitochondrial proteins, leading to impaired electron transport and energy failure [238, 239]. ONOO⁻ nitrates mitochondrial and cellular proteins, exacerbating OS and neuronal loss in the substantia nigra [238, 239]. Concurrently, activated glial cells release proinflammatory NO, amplifying oxidative/nitrative stress and sustaining neuroinflammation, which further damages dopaminergic neurons [238, 239].

RNS‐mediated nitration disrupts DA metabolism and exacerbates mitochondrial dysfunction, while genetic variations in NO synthase (nNOS) may increase susceptibility by disturbing NO balance [215, 240, 241]. Experimental models show that reducing nitrative stress through NOS inhibition lessens neuronal death and motor deficits, suggesting nitrative stress as a significant mechanism in PD pathogenesis [215, 240‐242].

#### 4.1.3 Huntington's Disease

Huntington's disease (HD) is a fatal autosomal dominant neurodegenerative disorder primarily caused by abnormal CAG repeat expansions in the huntingtin gene. The disease is characterized by selective degeneration of the striatum, manifesting as choreiform movements, progressive dementia, and dystonia. HD pathogenesis involves complex mechanisms attributed to mutant huntingtin protein (mHTT), which induces cellular dysfunction and neuronal death. Emerging evidence highlights the critical role of OS in HD pathology, particularly through iron dysregulation and mitochondrial dysfunction [215, 243, 244, 245].

Iron accumulation demonstrates a strong association with OS in HD patients and animal models. Postmortem analyses reveal elevated iron levels in the caudate nucleus and striatum of HD patients [246]. This iron overload exacerbates OS and disrupts cellular redox homeostasis. Mechanistically, reduced expression of iron regulatory proteins (IRP1/IRP2) coupled with increased TFR levels indicates impaired iron metabolism regulation. Concurrent upregulation of ferroportin may paradoxically exacerbate intracellular iron accumulation, potentiating ferroptosis [247]. Therapeutic interventions using iron chelators like desferrioxamine (DFO) improve motor and cognitive deficits in HD mice, substantiating the pathogenic role of iron‐mediated OS [248].

OS further intersects with mitochondrial dysfunction in HD pathogenesis. As primary ROS production sites, damaged mitochondria in HD exhibit enhanced ROS generation. mHTT induces mtDNA damage and compromises respiratory chain activity, aggravating OS [209, 243]. Through direct suppression of peroxisome proliferator‐activated receptor gamma coactivator 1α (PGC‐1α), mHTT disrupts mitochondrial biogenesis and amplifies ROS production [243, 249]. Additionally, calcium homeostasis dysregulation and altered mPTP dynamics promote apoptotic signaling cascades [209, 243, 249].

LPO driven by OS critically impacts neuronal survival. Ferroptosis inhibitors (e.g., Fer‐1) significantly attenuate oxidative lipid damage and neuronal death in HD cellular models, identifying LPO as a potential therapeutic target [250]. RNAi screening implicates arachidonate 5‐lipoxygenase (ALOX5) as a key mediator in mHTT‐induced ferroptosis, with ALOX5 deficiency abolishing polyglutamine of mHTT (HTTQ94)‐triggered ferroptosis under oxidative conditions. GPX4 further emerges as a crucial antioxidant defense component, modulating LPO and ferroptosis to support neuroprotective mechanisms [247].

#### 4.1.4 Major Depressive Disorder

Major depressive disorder (MDD), a psychiatric condition characterized by persistent low mood and anhedonia, has emerged as a leading global cause of mental and physical disability. Growing evidence implicates OS as a critical pathogenic contributor to MDD through mechanisms involving cellular damage, inflammatory responses, and eventual cell death. Excessive ROS disrupt neural signaling pathways and interact with cellular lipids, proteins, and nucleic acids, compromising neuronal structural integrity and functional capacity.

Epidemiological investigations and animal studies collectively demonstrate the association between disrupted metal ion homeostasis and emotional dysregulation. Iron accumulation and dysregulated iron metabolism show significant correlations with depressive symptom severity [251]. Experimental evidence reveals that ferroptosis inhibitors (e.g., Fer‐1) ameliorate depression‐like behaviors and promote neuronal growth, suggesting OS–ferroptosis crosstalk as a potential regulatory axis in depression pathology [251].

Beyond neuronal death, OS interacts with neuroinflammatory processes in MDD pathogenesis. Oxidative damage‐derived molecules activate innate immune responses and sterile inflammation, driving proinflammatory cytokine production [252, 253]. Clinical and preclinical studies consistently demonstrate elevated OS markers (8‐OHdG and malondialdehyde [MDA]) correlating with depressive symptom severity in MDD patients and animal models, highlighting oxidative‐neuroinflammatory interplay as a potential disease mechanism [254, 255].

MDD patients exhibit altered antioxidant enzyme activities, with SOD and GPX levels correlating with clinical severity. Concurrent depletion of nonenzymatic antioxidants (e.g., vitamins C/E) further compromises systemic antioxidant capacity, exacerbating oxidative damage. Dietary supplementation with bioactive antioxidants (e.g., N‐acetylcysteine [NAC]) shows potential adjunctive benefits in mitigating depressive symptoms through redox homeostasis restoration [252].

#### 4.1.5 Neuropathic Pain

Neuropathic pain (NeP) represents a prevalent and progressive neurological disorder characterized by spontaneous or evoked pain accompanied by heightened pain hypersensitivity and hyperreactivity. Emerging evidence implicates OS as a critical contributor to NeP pathogenesis through cellular damage promotion and chronic pain development and maintenance. The primary endogenous sources of OS involve NOX enzymes and mitochondrial ETCs, with NOX‐derived ROS constituting a major pathogenic component [252]. Experimental studies demonstrate that OS exacerbates neuronal damage and modulates nociceptive signaling pathways, significantly enhancing pain sensitivity in murine models [256].

OS interacts synergistically with LPO in NeP progression. ROS‐induced LPO degrades membrane fatty acids, compromising neural cell integrity. Concurrent neuroinflammatory responses amplify oxidative damage, establishing a vicious cycle that perpetuates pain perception. Spinal cord neurons exhibit direct functional impairment correlated with accumulated LPO products [256, 257, 258]. Furthermore, OS extends beyond neuronal effects to microglial activation, where ROS‐mediated crosstalk between activated microglia and astrocytes drives neuroinflammation through cytokine release and secondary ROS generation [256, 257, 258].

Clinical investigations document reduced antioxidant enzyme activity and elevated OS biomarkers in chronic pain patients. This redox imbalance influences neuronal survival while inducing maladaptive plasticity in central nervous system pain processing pathways. ROS accumulation parallels increased proinflammatory cytokine levels, suggesting mechanistic involvement in peripheral and central sensitization processes [256, 257, 258]. The progressive nature of oxidative damage underscores its dual role as both consequence and driver of NeP pathophysiology.

#### 4.1.6 Traumatic Brain Injury

Traumatic brain injury (TBI) represents a major global cause of disability and mortality, with OS serving as a critical pathological mediator postinjury. Following TBI, ROS production surges dramatically, overwhelming endogenous antioxidant defenses. This redox imbalance induces oxidative damage to cellular membranes, proteins, and glycolipids, ultimately compromising neuronal structural integrity and functionality while amplifying inflammatory responses and exacerbating secondary ischemic‐reperfusion injuries [259].

Emerging evidence links cerebral iron dyshomeostasis to OS pathogenesis in TBI. Mechanical trauma disrupts blood–brain barrier (BBB) integrity and induces intracranial hemorrhage, facilitating iron accumulation that perpetuates ROS generation through self‐amplifying cycles. Such iron overload not only correlates with neuronal dysfunction but also potentiates neurodegenerative processes [260, 261]. The postsynaptic density protein 95 (PSD95) critically mediates N‐methyl‐d‐aspartate receptor–PKCα coupling and interacts with neuronal nNOS to enhance NO production, thereby intensifying OS [260, 261, 262]. Post‐TBI temporal analyses reveal progressive reductions in synaptic markers including PSD95, synapsin I, and synapse‐associated protein 97 (SAP‐97), reflecting dynamic synaptic remodeling [260, 261, 262]. Concurrent glutamate excitotoxicity—driven by BBB disruption, vesicular release mechanisms, and transporter dysregulation—induces profound synaptic alterations that compound neural damage [260, 261, 262].

Acute‐phase cytokine storms involving IL‐1β, TNF‐α, and transforming growth factor (TGF‐β) induce cerebral inflammation and compromise BBB integrity post‐TBI. IL‐1β elevation promotes cerebral edema and neuronal loss, while TNF‐α dysregulation disrupts synaptic plasticity and central nervous system ion homeostasis, directly correlating with neurological deficits [260]. Therapeutic strategies targeting oxidative pathways show promise: melanin derivatives mitigate neuronal injury through MT2/IL‐33 pathway activation and ROS reduction, while Nrf2 overexpression counteracts TBI‐induced ferroptosis and synaptic damage via enhanced antioxidant responses [260].

Iron chelation therapy demonstrates clinical potential by reducing cerebral iron deposition and apoptosis, alleviating acute edema and chronic neurotoxicity. Natural Nrf2 activators like curcumin exhibit neuroprotective effects in preclinical models by boosting neuronal antioxidant capacity [263, 264]. These interventions highlight redox modulation as a viable therapeutic axis in TBI management.

#### 4.1.7 Spinal Cord Injury

Spinal cord injury (SCI) constitutes a severe neurological trauma characterized by high mortality and significant disability. Emerging evidence highlights the critical involvement of OS during the acute phase of SCI, with this process involving not only increased ROS production but also compromised antioxidant defense mechanisms [265, 266]. Post‐SCI, ROS levels surge dramatically, encompassing elevated superoxide, H_2_O_2_, and hydroxyl radical concentrations that directly correlate with pathological progression [265, 266].

OS exhibits a reciprocal relationship with mitochondrial dysfunction in SCI pathogenesis [266, 267, 268]. mtROS generation activates phospholipases and related enzymes, triggering membrane fatty acid release and impairing ATP synthesis [267, 268]. This pathological cascade disrupts intracellular ion homeostasis, particularly elevating sodium and calcium concentrations, which amplifies oxidative cytotoxicity and cellular damage while potentiating neutrophil activation and secondary ROS generation [267, 268].

In the context of inflammatory responses, OS drives microglial activation and proliferation, initiating proinflammatory cytokine release that reciprocally enhances ROS production‐establishing a self‐perpetuating cycle [266]. Upregulated inflammatory mediators including IL‐6 and TNF‐α demonstrate strong OS correlations, actively promoting neuronal apoptosis and exacerbating injury severity post‐SCI [266].

The Nrf2 signaling pathway emerges as a crucial regulatory mechanism in OS management [266]. Nrf2 activation enhances antioxidant enzyme expression through coordinated transcriptional regulation, restoring redox homeostasis and mitigating SCI‐associated oxidative damage [266]. This endogenous defense system represents a promising therapeutic target for modulating secondary injury processes.

### 4.2 Cancers

#### 4.2.1 The Epithelial–Mesenchymal Transition

Epithelial–mesenchymal transition (EMT), a pivotal biological mechanism in cancer pathology, involves the phenotypic and functional transformation of epithelial cells into mesenchymal‐like states. EMT activation frequently serves as a critical driver of tumor cell migration, invasion, and metastasis during cancer progression [269, 270], with OS emerging as key regulatory modulators of this process.

Accumulating evidence indicates elevated ROS levels during EMT in multiple cancer types, leading to intracellular labile iron pool accumulation and heightened ferroptosis susceptibility [271, 272]. For instance, in melanoma models, TGF‐β1 not only induces EMT phenotypes but also upregulates antioxidant enzymes GPX4 and solute carrier family 7 membrane 11 (SLC7A11) [273]. TGF‐β1‐driven EMT correlates with ROS generation, where enhanced OS paradoxically reduces ferroptosis resistance in post‐EMT cells, underscoring redox regulation in EMT–ferroptosis crosstalk [273].

OS extends beyond direct effects on proliferation/apoptosis to reinforce tumor aggressiveness via EMT‐related TFs and signaling pathways [274]. mtROS generation, for example, promotes hypoxia‐induced EMT in alveolar epithelial cells [275]. ROS‐mediated OS facilitates E‐cadherin downregulation—a hallmark EMT event marking epithelial identity loss [274, 276]. A critical regulatory axis involves ROS amplification of TGF‐β signaling, where Smad complex activation drives downstream EMT gene networks [274, 277, 278]. Consequently, mesenchymal marker upregulation (vimentin, fibronectin) and E‐cadherin suppression exhibit strong OS dependencies [274, 276].

Within tumor microenvironments, ROS modulate cancer‐associated fibroblast (CAF) behavior through HIF‐1α adaptation mechanisms [274, 279]. CAFs exhibit dual redox regulation by elevating ROS production while simultaneously activating antioxidant gene expression, a strategy influencing epithelial plasticity and potentially enhancing tumor invasiveness and therapy resistance [274, 279, 280]. This bidirectional interplay highlights OS as both a facilitator and consequence of EMT‐driven malignancy.

#### 4.2.2 Cancer Growth

The interplay between cancer progression and OS is complex and bidirectional. Excessive ROS generation drives cellular damage, promotes tumor proliferation and metastasis, yet paradoxically may induce apoptosis under specific conditions. Substantial evidence highlights OS as a critical facilitator of tumor cell growth, migration, and chemoresistance across multiple cancer types.

ROS critically influence cellular proliferation, survival, and apoptosis through diverse signaling cascades. The MAPK pathway, a key OS‐responsive axis, mediates ROS‐induced phosphorylation and activation of TFs AP‐1 and NF‐κB, thereby regulating survival‐, proliferation‐, and apoptosis‐associated gene networks [212, 281, 282].

The PI3K/Akt survival pathway is similarly modulated by ROS. ROS directly activate PI3K, triggering downstream Akt phosphorylation. Activated Akt suppresses proapoptotic factors (e.g., caspase‐9), thereby enhancing tumor cell survival [283, 284, 285]. Concurrently, the Keap1–Nrf2 system serves as a master OS regulator. Under redox imbalance, Nrf2 escapes Keap1‐mediated degradation, translocates to the nucleus, and activates antioxidant gene expression, bolstering cellular defense against oxidative damage [286, 287, 288, 289].

ROS further regulate the JAK/STAT axis by inducing JAK activation and subsequent STAT phosphorylation. Nuclear‐translocated STAT proteins modulate genes governing proliferation, inflammation, and apoptosis. Aberrant JAK/STAT activation correlates with tumor progression and poor prognosis in multiple malignancies [290, 291, 292, 293]. The Wnt/β‐catenin pathway, activated under OS, promotes tumorigenesis through β‐catenin nuclear accumulation and transcriptional activation of proproliferative genes [294, 295, 296, 297].

The p53 pathway exhibits dual roles in OS responses. ROS‐induced DNA damage activates p53‐mediated cell cycle control and repair mechanisms. Irreparable damage triggers p53‐dependent apoptosis, constraining tumor growth. This tumor‐suppressive function underscores critical role of p53 in genomic stability maintenance and cancer cell fate determination [298, 299, 300, 301].

Notably, ROS exert concentration‐dependent effects: while low levels support tumor proliferation, excessive ROS induce senescence or cell death. This dual regulatory role necessitates precise redox homeostasis in cancer cells to survive high‐stress microenvironments, highlighting the delicate balance between protumorigenic and cytotoxic ROS thresholds [302].

Besides, OS can reshape the tumor microenvironment through induction of cellular senescence and the senescence‐associated secretory phenotype (SASP). Persistent ROS accumulation triggers irreversible growth arrest in cancer cells, leading to SASP secretion of proinflammatory factors (e.g., IL‐6, IL‐8, VEGF) that paradoxically fuel tumor progression despite cell cycle exit [301].

#### 4.2.3 Cancer Metastasis

Cancer metastasis, a critical phase in malignant progression, involves the dissemination of primary tumor cells to distant sites. OS plays a multifaceted yet pivotal role in this process, not only promoting cancer cell proliferation and survival but also modulating metastatic efficiency through context‐dependent induction of cell death.

The crosstalk between ROS and TGF‐β signaling profoundly influences metastatic cascades [303]. As a key driver of advanced cancer progression, TGF‐β activity is redox‐regulated, with ROS amplifying its signaling to upregulate metastasis‐associated genes [303]. Mechanistically, ROS enhance the expression of prometastatic proteins (e.g., SNAIL, SLUG) while suppressing E‐cadherin in pancreatic ductal adenocarcinoma, thereby augmenting tumor invasiveness [303].

Hypoxic microenvironments further potentiate metastatic progression by elevating ROS levels. HIFs coordinate with TGF‐β signaling to regulate metastasis‐related gene networks, enhancing tumor cell aggressiveness. This ROS–hypoxia interplay exacerbates tumor malignancy, enabling cancer cells to thrive in complex microenvironments [303].

Fibrotic remodeling, particularly TGF‐β‐driven fibrosis, contributes to metastasis by fostering immune suppression [303]. ROS overproduction strongly correlates with fibrogenesis, which remodels the tumor microenvironment and impairs immune cell function, facilitating immune‐evasive cancer dissemination [303].

#### 4.2.4 Drug Resistance in Cancer

Cancer drug resistance, a major cause of therapeutic failure, severely impacts patient survival and quality of life. OS plays a dual role in resistance mechanisms during chemotherapy and targeted therapies.

In cancer cells, ROS modulate survival and drug resistance through redox‐sensitive signaling pathways. Subtoxic ROS levels promote prosurvival and antiapoptotic pathways, whereas excessive ROS may induce mutagenic adaptations underlying resistance [304]. Drug‐resistant tumors often exhibit enhanced antioxidant defenses via upregulated GSH and SOD, mitigating ROS accumulation to evade drug toxicity [305].

ROS‐regulated TFs, including Nrf2 and NF‐κB, orchestrate resistance networks. Nrf2 activation under OS induces ARE‐driven cytoprotective gene expression, correlating with multidrug resistance across cancers [306]. NF‐κB concurrently enhances survival by upregulating antiapoptotic genes and antioxidant pathways [304, 306].

Therapeutic strategies targeting redox homeostasis show promise in reversing resistance. ROS‐boosting agents or antioxidant inhibitors may sensitize resistant cells to chemotherapy. Preclinical studies demonstrate that selective inhibition of antioxidant enzymes elevates intracellular ROS, restoring chemosensitivity and improving therapeutic efficacy [305]. Combinatorial regimens integrating conventional therapies with redox modulators represent a rational approach to overcome resistance.

#### 4.2.5 Inhibition of ROS‐Induced Ferroptosis in Cancers

Ferroptosis is a distinct iron‐dependent regulated cell death modality triggered by the accumulation of lipid peroxides produced through ROS. This ROS‐induced ferroptosis functions as a powerful tumor‐suppressive mechanism by selectively eliminating cancer cells. Excessive ROS and LPO compromise cancer cell viability, thereby inhibiting tumor growth and progression.

Key tumor suppressors, such as p53 and BAP1, promote ferroptosis by repressing the expression of antioxidant defense components like SLC7A11, a subunit of the cystine/glutamate antiporter system Xc− responsible for cystine uptake required for GSH synthesis, and GPX4 [307, 308, 309]. Downregulation of SLC7A11 and GPX4 diminishes cellular antioxidant capacity, allowing ROS and lipid peroxide accumulation to trigger ferroptotic cell death. Thus, impairment of the GSH/GPX4 axis facilitates ROS‐mediated ferroptosis, effectively suppressing tumor development through iron‐dependent oxidative destruction of malignant cells [310, 311, 312].

The intrinsic metabolic rewiring of cancer cells often results in elevated ROS levels, which makes them particularly vulnerable to ferroptosis. Moreover, factors within the tumor microenvironment, including immune cells such as cytotoxic CD8+ T cells, enhance ferroptosis by secreting interferon‐γ (IFN‐γ), which downregulates SLC7A11 expression in cancer cells, further sensitizing them to ROS‐induced ferroptosis [313].

### 4.3 Cardiovascular Diseases

The centrality of mtROS in driving pathology unites seemingly disparate systems. In AD, defective mitophagy allows ROS accumulation that triggers tau hyperphosphorylation. Similarly, doxorubicin (DOX)‐induced cardiotoxicity arises from drug binding to cardiac mtDNA. Despite shared mitochondrial origins, therapeutic strategies diverge: neuronal protection emphasizes antioxidant gene upregulation (e.g., Nrf2), whereas cardiac interventions target iron chelation to prevent ferroptosis.

#### 4.3.1 Atherosclerosis

AS, a complex chronic disease intricately linked to OS, involves multifaceted redox mechanisms throughout its initiation and progression. This section delineates the distinct roles and molecular pathways of OS in AS pathophysiology.

During early atherogenesis, endothelial cells exposed to hyperglycemia, hyperlipidemia, and hemodynamic shear stress exhibit elevated ROS production [314, 315, 316]. ONOO⁻ oxidizes tetrahydrobiopterin (BH4) to BH2, triggering eNOS uncoupling. This shifts eNOS from NO generation toward superoxide production, further diminishing NO bioavailability and exacerbating oxidative and nitrative stress [314, 315, 316]. The resulting endothelial dysfunction impairs vasodilation and favors proinflammatory signaling through NF‐κB activation, promoting leukocyte recruitment and glycocalyx disruption. Simultaneously, ONOO⁻ modifies LDL components, accelerating LDL oxidation and foam cell formation, key steps in plaque development [314, 315, 316]. In vascular smooth muscle cells (VSMCs), RNS‐induced mitochondrial damage contributes to cellular calcification and vascular stiffness by disrupting NO–guanylate cyclase signaling [317, 318]. Thus, sustained RNS accumulation perpetuates a proatherogenic environment characterized by endothelial dysfunction, inflammation, and vascular remodeling [317, 318].

LDL oxidation generates atherogenic oxLDL—a pivotal driver of plaque development [316, 319, 320]. oxLDL promotes endothelial dysfunction and proinflammatory responses, facilitating monocyte adhesion and transmigration. Subendothelial accumulation of oxLDL‐laden macrophages as foam cells accelerates lipid deposition and fatty streak formation [316, 319, 320].

Macrophages exhibit dual roles in AS progression [316, 321‐323]. Initially, oxLDL uptake via lectin‐like oxLDL receptor 1 receptors induces foam cell formation and ROS overproduction, which activates NF‐κB signaling to release proinflammatory cytokines (TNF‐α, IL‐6). This chemotactic cascade recruits additional immune cells, amplifying inflammatory feedback loops [316, 321‐323].

VSMC dynamics are redox‐modulated in AS [324, 325, 326]. ROS stimulate VSMC proliferation and migration via MAPK/PI3K/NF‐κB pathways, contributing to neointimal hyperplasia [327, 328, 329]. Paradoxically, excessive ROS may induce VSMC apoptosis, reducing fibrous cap‐forming cells and destabilizing atherosclerotic plaques [324, 325, 326].

ER stress, exacerbated by ROS and homocysteine, activates apoptosis signal regulating kinase‐1 (Ask1) and upregulates ER stress markers (C/EBP homologous protein, phosphorylated protein kinase R‐like ER kinase, glucose regulated protein 78) alongside inflammatory mediators (NLRP3, IL‐1β, caspase1) [330, 331, 332]. ER stress synergizes with cholesterol dysmetabolism to enhance macrophage apoptosis and inflammatory responses, further accelerating atherogenesis [330, 331, 332].

ROS‐mediated apoptosis and necrosis critically fuel plaque progression [333]. Oxidative damage to endothelial cells, VSMCs, and macrophages activates p53 and caspase pathways, promoting cellular debris accumulation and lipid core expansion [333]. Necrotic cell‐derived prorepair signals paradoxically amplify local inflammation, establishing a self‐perpetuating cycle of vascular injury [333].

#### 4.3.2 Heart Failure

Heart failure, a chronic cardiovascular disorder predominantly affecting the elderly, is characterized by pathological alterations including myocardial hypertrophy and cardiac fibrosis. OS plays a pivotal role in its pathogenesis, primarily mediated through ROS generation and accumulation that inflict myocardial damage and pathological remodeling.

Under pathological conditions, mitochondrial ETC dysfunction drives excessive O_2_•− production, inducing oxidative cardiomyocyte injury [334]. NOX activity and expression are markedly upregulated by mechanical stretch, angiotensin II, and TNF‐α, correlating with heart failure progression [334]. Concurrently, xanthine oxidase hyperactivity contributes to ROS overproduction, while uncoupled NOS generates pathological O_2_•− under stress, exacerbating cardiac structural remodeling and contractile dysfunction [334].

A hallmark of heart failure involves the progressive depletion of endogenous antioxidant defenses under chronic OS. Experimental models demonstrate significant reductions in key antioxidant enzymes—SOD, CAT, and GPX—impairing cellular redox homeostasis and amplifying oxidative damage [335]. GSH depletion, closely associated with myocardial dysfunction, correlates with elevated TNF‐α levels postinjury and inversely relates to disease severity in advanced dilated or ischemic cardiomyopathy [334].

OS orchestrates myocardial remodeling through redox‐sensitive signaling cascades. ROS activate prohypertrophic pathways including Src tyrosine kinase, Ras GTPase, and MAPK, perturbing intracellular calcium homeostasis to induce cardiomyocyte hypertrophy, apoptosis, and fibrosis [334]. Mechanistically, ROS stimulate TFs NF‐κB and AP‐1, driving proinflammatory cytokine secretion (e.g., IL‐1β) that amplifies local inflammation and cardiac tissue injury [336].

ROS‐mediated apoptosis critically exacerbates heart failure progression. DNA and mitochondrial damage triggered by ROS activates proapoptotic pathways, accelerating cardiomyocyte loss [337]. This apoptotic cascade synergizes with fibrotic remodeling, as activated fibroblasts deposit collagen via TGF‐β signaling, further compromising cardiac architecture and function [337]. The interplay between OS, apoptosis, and fibrosis establishes a self‐reinforcing cycle that perpetuates heart failure pathogenesis.

#### 4.3.3 DOX Induced Cardiomyopathy

DOX, a widely used chemotherapeutic agent, is limited by its cardiotoxic effects and propensity to induce cardiomyopathy. OS constitutes a central mechanism in DOX‐induced cardiomyopathy, driving biochemical cascades that impair cardiomyocyte function and survival.

DOX undergoes enzymatic reduction via NADH dehydrogenase and eNOS to form semiquinone radicals. These radicals undergo redox cycling in the presence of oxygen, regenerating the parent quinone while generating O_2_•− [338]. SOD catalyzes O_2_•− conversion to H_2_O_2_, which subsequently generates •OH via Fenton chemistry, inducing oxidative damage to DNA, lipids, and proteins, ultimately triggering cardiomyocyte apoptosis [338].

DOX further exacerbates OS through iron overload mechanisms. By chelating Fe^3+^ and reducing it to Fe^2+^, DOX facilitates Fenton reactions where Fe^2+^ reacts with H_2_O_2_ to produce cytotoxic •OH [338, 339, 340, 341]. Myocardial iron accumulation correlates with cardiomyopathy progression, as iron–ROS interplay promotes LPO and ferroptosis—a regulated cell death pathway characterized by membrane integrity loss and iron‐dependent oxidative damage [338, 339, 340, 341].

Endogenous antioxidant defenses are critically compromised in DOX cardiotoxicity [341, 342]. Downregulation of SOD and GPX, particularly the lipid peroxide‐detoxifying enzyme GPX4, depletes cellular antioxidant capacity, leading to lethal LPO and cardiomyocyte vulnerability [341, 342].

The Nrf2–Keap1 axis, a master regulator of redox homeostasis, is dysregulated in DOX‐induced injury [341, 343, 344]. Under physiological conditions, Keap1 targets Nrf2 for proteasomal degradation. OS triggers Nrf2 nuclear translocation to activate antioxidant genes, yet DOX suppresses Nrf2 signaling, diminishing cytoprotective responses [341, 343, 344].

The redox‐sensitive p66Shc protein and its regulator sirtuin 1 (Sirt1) modulate cardiomyocyte ROS susceptibility. DOX upregulates p66Shc while downregulating Sirt1, amplifying oxidative damage [338, 339, 345, 346]. Concurrently, NOX isoforms (NOX2/NOX4) contribute to ROS generation via single‐electron transfer mechanisms, exacerbating myocardial injury [338, 339, 345, 346]. These interconnected pathways highlight the multifaceted redox dysregulation underlying DOX cardiotoxicity.

#### 4.3.4 Metabolic Cardiomyopathy

Metabolic cardiomyopathy, characterized by myocardial triglyceride accumulation and hyperglycemia‐induced insulin resistance, predominantly manifests in obese individuals. High‐glucose and high‐fat diets trigger deleterious oxidative modifications of metabolic regulatory proteins, driving maladaptive tissue remodeling that exacerbates left ventricular diastolic dysfunction.

In diabetic patients, elevated blood glucose directly amplifies mtROS production. Hyperglycemic conditions alter mitochondrial metabolism, particularly increasing electron leakage from the ETC, which elevates O_2_•− generation [338, 339, 345, 346]. While cardiomyocyte antioxidant systems typically neutralize ROS, diabetic pathophysiology compromises these defenses. Overexpression of poly(ADP‐ribose) polymerase (PARP), a biomarker of OS and DNA damage, depletes nicotinamide adenine dinucleotide [oxidized form] (NAD^+^) reserves, disrupting energy metabolism and cellular survival. This establishes a vicious cycle where ROS overaccumulation and DNA damage reciprocally amplify myocardial injury [347, 348].

Mitochondria serve as pivotal mediators of diabetic OS. Cardiac mitochondrial ETC constitutes a major ROS source, with chronic ROS production impairing physiological processes such as NO‐mediated vasodilation [347, 348]. Reduced NO bioavailability aggravates myocardial ischemia and cardiac dysfunction. Beyond oxidizing lipids, proteins, and DNA, excessive ROS activate apoptotic pathways, inducing programmed cardiomyocyte death and functional deterioration [347, 348].

Elevated free fatty acid (FFA) levels in diabetes further potentiate oxidative injury. Impaired insulin‐mediated glucose metabolism promotes cardiac FFA accumulation, which activates NOX to generate O_2_•−, intensifying intracellular OS [347, 348]. NOX‐derived O_2_•− reacts with NO to form ONOO⁻, a potent oxidant causing cellular damage. FFA metabolism‐generated ROS additionally drive membrane LPO, disrupting cellular architecture and exacerbating metabolic dysfunction [349, 350, 351].

Despite endogenous antioxidant defenses—including SOD and GSH—diabetic conditions suppress their activity, heightening cellular vulnerability [349, 350, 351]. Oxidative byproducts like MDA and oxLDL elicit robust inflammatory responses, establishing a redox‐inflammatory feedback loop that accelerates cardiac pathology progression [349]. This interplay underscores the critical role of OS in diabetic metabolic cardiomyopathy pathogenesis.

#### 4.3.5 Heart Transplantation

Cardiac transplantation remains a critical intervention for end‐stage heart disease, yet postoperative OS poses significant challenges. OS in this context involves multifaceted mechanisms that may impair graft function and compromise long‐term outcomes. This section delineates key pathways underlying OS in cardiac transplantation.

Ischemia/reperfusion (I/R) injury constitutes a primary OS trigger during transplantation. The excision of the donor heart induces ischemic hypoxia, followed by abrupt oxygen reintroduction during reperfusion. This transition triggers uncontrolled mitochondrial ETC activity, generating O_2_•− [352]. Concurrent NOX activation further amplifies ROS production, directly damaging cardiomyocytes through apoptosis, necrosis, and functional impairment [352].

Postreperfusion ROS activate innate inflammatory pathways, notably NF‐κB signaling, which drives proinflammatory cytokine release (TNF‐α, IL‐1, IL‐6) [352]. These cytokines reciprocally enhance ROS generation and recruit immune cells to the graft, establishing a self‐perpetuating cycle of oxidative‐inflammatory injury [353, 354, 355]. This crosstalk elevates acute graft damage risk and adversely impacts long‐term survival [353, 354, 355].

Mainstay immunosuppressive therapies paradoxically exacerbate OS. Cyclosporine A, while preventing rejection, suppresses endogenous antioxidant defenses (e.g., GSH reductase), heightening cellular redox vulnerability. Chronic immunosuppressant use thus amplifies oxidative injury risk, potentially accelerating graft dysfunction over time [356, 357].

Posttransplant metabolic derangements (hyperglycemia, dyslipidemia) synergize with OS. Hyperglycemia augments mtROS via enhanced electron leakage and advanced glycation end (AGE)‐product formation. Elevated FFA activate NOX‐dependent ROS generation, while FFA‐β‐oxidation generates LPO byproducts [352, 356]. These metabolic–oxidative perturbations impair graft healing, promote maladaptive remodeling, and hasten functional decline.

Collectively, these mechanisms underscore OS as a modifiable therapeutic target to improve cardiac transplantation outcomes. Strategic interventions balancing immunosuppression with redox homeostasis may mitigate graft injury and enhance postoperative recovery.

#### 4.3.6 Aortic Dissection

Aortic dissection (AD), a life‐threatening cardiovascular condition, involves structural disintegration of the aortic wall leading to hematoma formation between the intimal and medial layers. Its pathogenesis involves multifactorial contributions including genetic predisposition, environmental triggers, and lifestyle factors, with OS serving as a pivotal mediator through effects on VSMCs, ECM, inflammatory responses, and endothelial function [358].

ROS overproduction induces VSMC dysfunction and programmed cell death in AD progression [358]. While healthy VSMCs maintain contractile phenotypes to withstand hemodynamic stress, chronic OS drives phenotypic switching to synthetic VSMCs characterized by enhanced proliferation and secretory activity [359, 360, 361]. This transition, mediated through MAPK pathway activation, correlates with upregulated MMP expression and structural destabilization of the aortic wall [359, 360, 361].

OS critically disrupts ECM homeostasis in AD [358, 362, 363]. Histopathological hallmarks including elastic fiber fragmentation and aberrant collagen deposition are redox‐regulated. ROS upregulate MMPs to degrade structural proteins (elastin, collagen), while impairing ECM synthesis [362, 363]. Combined VSMC loss and ECM degradation markedly reduce aortic tensile strength, accelerating dissection propagation and rupture risk [362, 363].

AD progression involves macrophage infiltration and activation, establishing a vicious cycle of oxidative‐inflammatory damage [358, 363, 364]. Inflammatory cells release ROS, proinflammatory cytokines, and MMPs that synergistically degrade ECM components and induce VSMC apoptosis. OS concurrently exacerbates endothelial dysfunction, further amplifying aortic vulnerability [363, 364].

Endothelial cells critically regulate vascular tone and integrity through NO synthesis [358, 365]. OS diminishes NO bioavailability, promoting VSMC hyperplasia and endothelial dysfunction. NO deficiency disrupts vascular homeostasis, exacerbating aortic wall remodeling and dissection risk [365]. These interconnected mechanisms underscore OS as a therapeutic target for AD management.

### 4.4 Liver Diseases

PUFA oxidation underlies membrane fragility in multiple systems. Atherosclerotic plaque rupture shares a LPO signature with alcoholic hepatitis's hepatocyte ballooning, both driven by 4‐hydroxynonenal (4‐HNE) adduct formation.

#### 4.4.1 Hepatotoxicity

OS plays a central role in hepatotoxicity through mechanisms involving excessive ROS generation and multifaceted cellular damage. Under physiological conditions, the liver maintains redox equilibrium between ROS production and clearance. However, external insults (e.g., drugs, alcohol, viral infections) induce mitochondrial dysfunction, triggering pathological ROS overproduction. Excessive ROS initiate LPO, attacking PUFAs in cellular membranes to generate cytotoxic aldehydes (MDA, 4‐HNE) that disrupt membrane integrity, permeability, and function [366, 367].

Oxidative protein damage induces irreversible denaturation and functional loss, activating apoptosis via p53, caspase, and Bcl‐2/Bax pathways [366, 367, 368]. Concurrently, ROS oxidatively modify DNA to form mutagenic adducts (e.g., 8‐OHdG), causing genomic instability, cell cycle arrest, and programmed cell death [366, 367].

OS synergizes with inflammatory responses through NF‐κB signaling activation, driving proinflammatory cytokine release (TNF‐α, IL‐6, IL‐1β). This redox‐inflammatory crosstalk perpetuates hepatic injury, establishing a self‐amplifying cycle that exacerbates liver pathology [366, 367].

Endogenous antioxidant defenses (SOD, GPX, reduced GSH) are overwhelmed under sustained OS. For instance, GPX4 downregulation in alcoholic liver disease (ALD) impairs ROS detoxification, accelerating hepatocyte injury [366, 367]. Mitochondrial integrity critically regulates redox homeostasis, as membrane potential collapse and respiratory chain defects amplify ROS generation, inducing bioenergetic failure and apoptosis [366, 367, 369]. These interconnected mechanisms highlight OS as both a driver and consequence of hepatotoxic injury.

#### 4.4.2 Alcoholic Liver Disease

ALD is a form of liver injury caused by chronic alcohol consumption, characterized by a complex pathogenesis in which OS plays a crucial role. Alcohol consumption not only exacerbates the generation of ROS within hepatocytes but also progressively weakens the antioxidant defense mechanisms of liver, creating a vicious cycle that ultimately leads to escalating levels of OS and progressively worsening liver damage.

First, the metabolic process of alcohol is a key factor in inducing OS. Ethanol is primarily metabolized in the liver by alcohol dehydrogenase (ADH) into acetaldehyde, which is subsequently converted into acetate by aldehyde dehydrogenase. This process significantly increases the production of NADH, enhancing the reductive state within the liver. This reductive state not only increases metabolic pressure on mitochondria but also leads to dysfunction in the ETC, promoting the excessive production of ROS [370, 371]. Particularly, when the NADH/NAD^+^ ratio decreases, the metabolic homeostasis of the liver is disrupted, reducing fatty acid oxidation while increasing fatty acid synthesis due to the enhanced reductive state, ultimately leading to fat accumulation and LPO in the liver [370, 371].

LPO is one of the direct consequences of OS and a significant mechanism of damage in ALD. Enhanced ROS reacts with PUFAs in cell membranes, promoting lipid oxidation and generating toxic LPO products such as LPO and 4‐HNE [372, 373]. Additionally, the effects of ethanol and acetaldehyde downregulate adiponectin and STAT3, inhibiting the activity of 5′‐AMPK and peroxisome proliferator‐activated receptor α (PPARα), ultimately promoting fatty acid accumulation in the liver and leading to LPO [372, 373]. These toxic compounds not only directly damage hepatocyte membranes, disrupting cell structure and function, but also bind to membrane proteins, affecting cell signaling and inducing apoptosis or necrosis, thereby increasing the extent of liver damage.

In the context of OS, DNA and proteins suffer varying degrees of oxidative damage. In ALD, DNA damage in hepatocytes often manifests as the accumulation of oxidative modifications, particularly an increase in 8‐OHdG [373]. This DNA damage not only leads to gene mutations but may also affect gene expression, halt DNA synthesis, initiate cell detection mechanisms, and cause cell cycle arrest [373]. Regarding protein oxidative damage, oxidation‐induced amino acid modifications alter the structure and function of key proteins. When proteins are oxidized, their structure and function change, potentially being recognized as new antigens by the immune system, further inducing autoimmune responses [373]. Tyrosine oxidation can form dityrosine, while cysteine oxidation produces sulfinic acid, sulfonic acid, and disulfide bonds, leading to protein cross‐linking and aggregation. Excessive protein oxidative damage disrupts cellular homeostasis, increasing the risk of hepatotoxicity and cell death [373]. These apoptotic signaling pathways, such as the activation of p53 and the caspase family, are directly related to ROS production, exacerbating hepatocyte damage [373].

OS also plays a role in ALD by modulating inflammatory responses. The excessive production of ROS can activate NF‐κB and other proinflammatory signaling pathways, enhancing the secretion of proinflammatory cytokines such as TNF‐α, IL‐6, and IL‐1β [370], not only causing local inflammation in the liver but also potentially triggering systemic immune responses, further aggravating hepatocyte damage. As liver inflammation worsens, the interaction between related cytokines and ROS forms a vicious cycle, leading to progressively worsening liver pathology [370].

Simultaneously, chronic alcohol consumption significantly impacts the antioxidant capacity of liver. This imbalance in antioxidant mechanisms is primarily reflected in the decreased expression levels of antioxidant enzymes. Key antioxidant enzymes such as SOD, GSH‐Px, and CAT typically exhibit significantly reduced activity under alcohol stimulation. This renders hepatocytes unable to effectively clear the increased ROS in the body, thereby exacerbating the degree of OS [374]. Additionally, alcohol consumption depletes GSH and other crucial antioxidants in the body, further weakening the resistance of liver to oxidative damage. This damage not only affects hepatocyte survival but also induces intracellular inflammatory responses, aggravating the progression of ALD [374].

#### 4.4.3 Liver Fibrosis and Cirrhosis

Hepatic fibrosis is a pathological condition arising from liver injury, characterized by hepatocyte apoptosis and excessive deposition of collagen and other ECM components in the hepatic stroma. OS plays a pivotal role in the initiation and progression of hepatic fibrosis, primarily through hepatocyte damage and activation of hepatic stellate cells (HSCs).

During fibrogenesis, OS is typically triggered by inflammatory factors, toxins, and viral infections, which induce excessive production of ROS in the liver. When hepatocytes are injured and mitochondrial dysfunction occurs, ROS generation is markedly elevated. These ROS not only directly cause oxidative damage to hepatocyte membranes, proteins, and DNA, thereby inducing apoptosis, but also stimulate the release of proinflammatory cytokines such as TNF‐α and IL‐1β [374], exacerbating hepatic inflammation.

As inflammation intensifies, activated Kupffer cells and neutrophils further contribute to ROS generation, collectively promoting HSCs activation and migration. ROS production primarily depends on NOX. In HSCs, the expression of NOX1, NOX2, and NOX4 drives excessive ROS generation, which in turn enhances HSC activation [376]. Experimental studies demonstrate that mice deficient in NOX regulatory components fail to produce ROS when exposed to angiotensin II, PDGF, leptin stimulation, or apoptotic body treatment, resulting in attenuated hepatic fibrosis in bile duct ligation or carbon tetrachloride‐induced models. These findings underscore the critical role of NOX in fibrotic progression [376]. Recent studies further reveal that NOX‐mediated effects extend beyond HSCs, as macrophage‐derived NOX1 promotes hepatocarcinogenesis through induction of inflammatory cytokines, highlighting the multifaceted roles of NOX in liver pathology.

Under OS, activated HSCs undergo sustained proliferation and transdifferentiation into myofibroblasts via multiple signaling pathways, including TGF‐β/Smad, leading to excessive collagen synthesis and ECM deposition that drives stromal hyperplasia and fibrotic scar formation [375]. Notably, OS not only enhances collagen production in HSCs but also inhibits their apoptosis, creating a self‐reinforcing loop that accelerates fibrogenesis [375].

A complex interaction network exists between OS and ECM remodeling during hepatic fibrosis. Persistent oxidative damage to hepatocytes amplifies inflammatory responses, further activating HSCs and aggravating fibrotic changes. Concurrently, activated HSCs secrete additional proinflammatory factors and cytokines that sustain elevated OS levels. This vicious cycle drives rapid progression and clinical deterioration of hepatic fibrosis [377].

#### 4.4.4 Liver Transplantation

OS plays a pivotal role in liver transplantation, profoundly influencing functional recovery and long‐term prognosis of the grafted liver. Both the surgical procedure itself and postoperative immunosuppressive therapy can induce substantial ROS production, triggering OS that causes structural and functional damage to the hepatic tissue.

During liver transplantation, the organ undergoes ischemia–reperfusion injury (IRI) resulting from temporary cessation of blood flow during donor liver procurement and implantation. Subsequent reperfusion leads to abrupt oxygen influx, driving excessive ROS generation. These radicals oxidize cellular components including lipids, proteins, and DNA, inducing structural and functional alterations that culminate in apoptosis or necrosis. Beyond direct hepatocyte damage, oxidative modifications disrupt cellular signaling pathways. Clinical evidence demonstrates strong correlations between elevated OS and posttransplant complications including hepatic dysfunction, cholestasis, and acute rejection episodes.

The accelerated ROS production during IRI primarily stems from enzymatic activation pathways. Key enzymes such as xanthine oxidase, NOX, and NOS catalyze molecular oxygen reduction to generate superoxide anions, substantially elevating ROS levels [378, 379, 380]. These radicals not only inflict direct hepatocellular damage but also initiate localized immune responses through damage‐associated molecular pattern (DAMP) release into the extracellular milieu following cellular injury. DAMPs subsequently activate innate immunity, perpetuating inflammatory cascades and secondary tissue damage [378, 379].

Postoperative immunosuppression paradoxically modulates OS dynamics. While mainstay agents like corticosteroids and calcineurin inhibitors effectively prevent graft rejection, certain immunosuppressants exhibit ROS‐promoting properties that exacerbate oxidative damage. This pharmacological paradox suppresses hepatic antioxidant defenses, potentially contributing to persistent posttransplant hepatic dysfunction [378, 379].

Chronic OS constitutes a critical mediator of graft deterioration and fibrogenesis. In long‐term transplant recipients, sustained oxidative injury promotes chronic rejection and hepatic fibrosis through inflammatory amplification and LPO cascades. The cumulative oxidative damage not only compromises hepatic architecture and function but also impairs regenerative capacity, ultimately diminishing long‐term graft survival rates.

### 4.5 Kidney Diseases

Fibrotic remodeling across organs converges on ROS‐activated TGF‐β signaling. HSCs and renal fibroblasts undergo oxidative phenotype switching, depositing pathological collagen. However, fibrotic drivers differ: NOX4 dominates renal interstitial fibrosis, whereas hepatic fibrosis relies on CYP2E1‐derived ROS.

#### 4.5.1 Nephrotoxicity

OS plays a pivotal role in the pathogenesis of nephrotoxicity, encompassing a complex interplay of biochemical reactions and cellular processes. Fundamentally, OS arises from an imbalance between the generation of ROS and the cellular antioxidant defense mechanisms, culminating in cellular dysfunction and tissue injury. Within the renal context, OS not only induces direct damage to renal tubular epithelial cells but also triggers inflammatory cascades and programmed cell death, ultimately contributing to the deterioration of renal function.

The molecular mechanisms underlying nephrotoxicity are mediated through multiple signaling pathways, particularly the MAPK family, including p38 MAPK, ERK, and JNK [381]. These pathways are intricately involved in OS responses and renal immune regulation. For example, activation of the p38–MAPK pathway facilitates IκB kinase‐mediated degradation of IκBα, leading to nuclear translocation and transcriptional activation of NF‐κB. This establishes a positive feedback loop that amplifies the release of proinflammatory cytokines, including TNF‐α and IL‐6 [381]. Concurrently, Nrf2, a master regulator of antioxidant responses, is activated under OS conditions to orchestrate the expression of cytoprotective genes, thereby maintaining intracellular redox homeostasis [381, 382]. The dynamic interplay between these pathways is critical in modulating OS and inflammatory responses in renal pathophysiology.

Apoptosis of renal tubular epithelial cells represents a hallmark of nephrotoxic injury, particularly in the context of drug‐induced or toxin‐mediated renal damage [383, 384]. The apoptotic cascade is predominantly initiated by excessive ROS generation, which disrupts intracellular calcium homeostasis and activates the factor‐related apoptosis ligand/factor‐related apoptosis (Fas ligand/Fas) receptor system. This leads to mitochondrial outer membrane permeabilization, cytochrome *c* release, and subsequent activation of the caspase‐3‐dependent apoptotic pathway. Such mechanisms render renal tubular cells particularly vulnerable to toxic insults, contributing to the progression of acute kidney injury (AKI) and renal failure. The loss of tubular epithelial cells compromises their regenerative capacity, thereby exacerbating renal structural and functional impairment and establishing a self‐perpetuating cycle of injury [384, 385].

ER stress has emerged as a critical contributor to nephrotoxicity. Under conditions of OS, renal cells experience ER dysfunction, characterized by the accumulation of misfolded or unfolded proteins within the ER lumen [385, 386]. This triggers the UPR, which initially activates autophagy as an adaptive mechanism to mitigate ER stress. However, sustained ER stress leads to the suppression of both ER function and autophagic flux, resulting in the accumulation of damaged cellular components [385, 386]. Mechanistic studies have demonstrated that ER stress‐induced cellular injury is closely associated with apoptotic pathways, which simultaneously inhibit renal antioxidant defense systems, ultimately culminating in cellular dysfunction and progressive renal damage [385].

Furthermore, exposure to nephrotoxic agents elicits robust inflammatory responses characterized by the release of proinflammatory mediators, including TNF‐α, IL‐1β, and IL‐6, which further potentiate OS. NF‐κB, a central transcriptional regulator of inflammation, plays a critical role in this process by enhancing the expression of proinflammatory cytokines, thereby establishing a vicious cycle between OS and inflammatory signaling. This inflammatory milieu not only exacerbates renal parenchymal injury but also disrupts the renal microenvironment, impairing normal physiological functions and contributing to the progression of chronic kidney disease (CKD) [383, 387‐389].

#### 4.5.2 Diabetic Nephropathy

OS plays a pivotal role in the pathogenesis of diabetic kidney disease (DKD), involving multiple biochemical pathways and cellular processes. Diabetes, a metabolic disorder characterized by chronic hyperglycemia, drives excessive ROS generation, leading to redox imbalance. This imbalance not only damages renal tissues but also exacerbates tubular and glomerular dysfunction.

Chronic hyperglycemia, a hallmark of diabetes, significantly promotes ROS overproduction, initiating metabolic disturbances. A critical mechanism involves the formation of AGE products (AGEs). These AGEs bind to cell surface receptors (e.g., RAGE), further stimulating ROS generation and inducing oxidative cellular damage [390, 391, 392]. Concurrently, AGEs trigger inflammatory responses by releasing proinflammatory cytokines such as TNF‐α and IL‐1β, amplifying OS and establishing a self‐perpetuating cycle of injury [390, 391, 392, 393]. Notably, NOX isoforms, particularly NOX4 and NOX5, serve as primary ROS sources in renal tubular cells and podocytes. Experimental evidence highlights that NOX4 upregulation correlates with DKD progression, as its activation enhances ROS generation and exacerbates oxidative renal injury. This sustained OS disrupts both tubular and glomerular function, accelerating DKD advancement [351].

A tightly interdependent relationship exists between OS and inflammation in DKD [18, 394]. Hyperglycemia‐induced ROS overproduction directly damages cells while stimulating the release of inflammatory mediators, including TNF‐α and IL‐6. Mechanistically, ROS activate NF‐κB, a master transcriptional regulator of proinflammatory cytokine expression, thereby amplifying inflammation. Moreover, ROS induce monocyte chemoattractant protein‐1 overexpression, promoting macrophage and T‐cell infiltration into renal tissues and aggravating injury [390, 391, 394]. TNF‐α further perpetuates this cycle by stimulating ROS production and modulating cellular proliferation/apoptosis through feedback mechanisms. This persistent crosstalk between OS and inflammation disrupts the renal microenvironment, impairing physiological function [390, 391, 394].

Renal fibrosis represents a critical pathological feature of DKD. Chronic ROS generation under hyperglycemic conditions activates renal stellate cells, driving excessive collagen and ECM deposition. This fibrotic process is tightly regulated by the activation of TGF‐β and connective tissue growth factor [390]. ROS enhance the expression of these profibrotic factors, stimulating fibroblast activity and ECM accumulation, which disrupt renal architecture and function. Progressive fibrosis impairs tubular and glomerular regenerative capacity, culminating in end‐stage renal disease [390]. Notably, AGEs synergistically accelerate DKD progression by amplifying inflammatory responses and activating fibrotic signaling pathways.

Mitochondria serve as a major ROS source in DKD pathogenesis. Hyperglycemia‐induced mitochondrial dysfunction exacerbates OS by impairing ETC efficiency [392]. Studies demonstrate that mitochondrial damage not only reduces ATP synthesis but also triggers cytochrome *c* release, activating apoptotic pathways. In renal endothelial cells and podocytes, mitochondrial dysfunction activates signaling cascades such as PI3K/Akt and NF‐κB, promoting inflammation, apoptosis, and fibrosis [392]. Furthermore, iron overload and LPO exacerbate mitochondrial injury, depleting antioxidant defenses and worsening diabetic renal damage. Thus, mitochondria act both as targets of OS and as critical mediators linking OS to cellular injury in DKD [392].

#### 4.5.3 Acute Kidney Injury

AKI is a heterogeneous clinical syndrome, which arises from a diverse array of underlying causes, including ischemia, nephrotoxins, and systemic inflammatory responses, leading to varying degrees of renal dysfunction. OS plays a pivotal role in the pathogenesis and progression of AKI, involving multiple signaling pathways and cellular responses.

IRI is a major cause of AKI, typically resulting from renal hypoperfusion followed by reperfusion, which triggers profound OS. During ischemia, ATP synthesis in renal tubular cells is markedly reduced, leading to impaired cellular metabolism and mitochondrial dysfunction [395, 396]. Ischemia disrupts the mitochondrial ETC, increasing ROS production [395, 396]. Upon reperfusion, the sudden influx of oxygen drives the rapid generation of superoxide and H_2_O_2_ by mitochondria, further exacerbating OS [395, 396]. Elevated ROS levels directly damage cellular membranes, proteins, and DNA, increasing membrane permeability and disrupting cellular integrity, ultimately leading to cell dysfunction and death. Concurrently, IRI activates the NF‐κB pathway, promoting the release of proinflammatory cytokines such as TNF‐α and IL‐6, which amplify local inflammation and oxidative damage, thereby inducing apoptosis [355, 397].

Sepsis is another critical contributor to AKI, often accompanied by systemic inflammatory responses and microcirculatory dysfunction. In that case, the immune system undergoes hyperactivation, with macrophages and neutrophils generating reactive species such as superoxide and ONOO⁻ to eliminate pathogens [398, 399]. However, these ROS also inflict damage on surrounding renal tubular and endothelial cells [398, 399]. Additionally, the activation of iNOS in sepsis produces excessive NO, which reacts with ROS to form ONOO⁻, a highly toxic compound that exacerbates tubular cell injury [398, 399, 400]. The release of DAMPs further amplifies inflammation by activating TLRs. The interplay between DAMPs and ROS creates a vicious cycle, intensifying local inflammation and OS [398, 399]. Moreover, sepsis‐induced microvascular dysfunction compromises renal blood supply, exacerbating cellular hypoxia and renal injury [398, 399].

Drug‐induced AKI involves multiple mechanisms that exacerbate OS [401]. For instance, during drug metabolism, cytochrome P450 enzymes in microsomes generate ROS. The detoxification process may lead to excessive ROS production, increasing the risk of oxidative damage [402]. Furthermore, the depletion of GSH, a critical antioxidant, results in ROS accumulation and impaired cellular antioxidant capacity. Certain drugs directly damage mitochondria, particularly at complexes I and III of the ETC, leading to increased O_2_•− generation. Mitochondrial membrane potential and permeability are also altered, impairing ATP synthesis and promoting cell death [403]. Drug‐induced ER stress causes the accumulation of misfolded proteins, activating the UPR and increasing ROS production, thereby elevating the risk of apoptosis [404, 405].

Viral infections contribute to AKI through multiple mechanisms, with OS being a central factor. During viral infections, biotransformation enzymes such as cytochrome P450 and xanthine oxidase are activated, leading to increased ROS production. The accumulation of ROS is closely associated with cellular and tissue damage. The respiratory burst of monocytes and macrophages during immune responses generates substantial ROS, which, while aiding viral clearance, also damages surrounding cells [406]. Additionally, certain viral infections may increase intracellular iron availability, enhancing the Fenton reaction to produce hydroxyl radicals, a potent oxidant that damages cellular membranes and biomolecules. Viral infections also induce ER and mitochondrial dysfunction, leading to sustained ROS production, exacerbating OS, and impairing cell survival and function [407].

#### 4.5.4 Chronic Kidney Disease

OS plays a crucial role in the initiation and progression of CKD, with mechanisms involving various biochemical reactions and cellular signaling pathways. In the context of CKD, OS is triggered by multiple factors, including prolonged hyperglycemia, hypertension, obesity, and chronic inflammation. The sustained generation of ROS leads to damage to renal tissue and promotes the progression of pathological changes.

Mitochondria serve as the primary centers for intracellular energy metabolism, and their normal functioning is essential for maintaining cellular health. Mitochondria produce ATP through the process of oxidative phosphorylation, but they also generate ROS. In the context of CKD, the ETC in mitochondria is impaired, resulting in excessive accumulation of ROS [408, 409]. Studies have shown that the overproduction of ROS not only directly causes damage to mitochondrial membranes and disrupts membrane potential, leading to apoptosis, but also induces oxidative damage to mtDNA. This damage is closely associated with the progression of CKD, and the limited repair mechanisms for mtDNA accelerate the loss of mitochondrial function and the decline of renal function [408, 409]. Moreover, the abnormal opening of the mPTP can lead to the release of cytosolic contents under pathological conditions, inducing apoptosis [408, 409].

ER stress is an significant factor in the progression of CKD. The ER is responsible for synthesizing and modifying cellular membrane and secretory proteins. When the ER encounters protein folding disorders or accumulates misfolded proteins, it activates the UPR, which restores ER homeostasis by promoting autophagy or inducing apoptosis [410, 411, 412]. The state of ER stress can result in loss of cell function and pathological damage [410, 411, 412]. Additionally, peroxisomes serve as the main antioxidant organelles in cells, responsible for removing intracellular ROS. Research has found that in patients with CKD, the decline in peroxisomal function prevents the efficient clearance of ROS, exacerbating OS and triggering a series of cellular injuries and interstitial fibrosis [410, 411, 412].

The relationship between OS and renal inflammation is closely intertwined, primarily achieved through the activation of various signaling pathways. ROS can activate TFs such as NF‐κB, promoting the release of inflammatory mediators, including TNF‐α and IL‐6. These inflammatory factors not only directly affect the function of tubular cells and glomerular endothelial cells but also stimulate the proliferation and activation of renal stellate cells, promoting the fibrotic process of the renal interstitium. Furthermore, the sustained release of proapoptotic proteins and other inflammatory mediators exacerbates renal cellular injury and the inflammatory response, forming a vicious cycle of exacerbating negative feedback [413].

eNOS plays a central role in maintaining endothelial function and microcirculatory stability. Dysfunction of eNOS results in a decreased generation of NO while increasing the production of ROS, thereby exacerbating endothelial dysfunction. The activity of endogenous NOS is influenced by various factors, particularly the depletion of BH4 and the accumulation of asymmetric dimethylarginine, which further aggravate eNOS dysfunction [409, 414]. In CKD patients, impaired endothelial function not only affects the microcirculation of the kidneys but also leads to ischemia of renal tissue, thereby exacerbating OS and cellular injury. Therefore, restoring endothelial cell function and eNOS activity may represent potential strategies for reversing the progression of CKD [409, 414].

#### 4.5.5 Kidney Transplantation

In kidney transplantation, OS not only affects the quality of the donor kidney and the restoration of function posttransplant but is also closely associated with postoperative complications such as acute rejection, chronic rejection, and renal failure.

In the context of kidney transplantation, abnormal accumulation of iron generates a large amount of ROS through the Fenton reaction, consequently leading to intracellular OS. During the IRI process of the donor kidney, iron accumulation can trigger ferroptosis by inhibiting the cystine/glutamate antiporter (XC system) [415, 416, 417]. The XC system is responsible for transporting cystine into cells for the synthesis of GSH, which is critical for the activity of GPX4 in eliminating lipid peroxides. Therefore, the inhibition of the XC system leads to decreased GPX4 activity, promoting the accumulation of lipid peroxides and initiating ferroptosis [415, 416, 417]. Against this backdrop, protective mechanisms against ferroptosis have gradually gained attention, including the modulation of iron metabolism, the application of antioxidants such as vitamin E, and related enzymes like CoQ10, emphasizing the potential to protect cells from OS.

Mitochondria are the main energy metabolism centers within cells, and their proper functioning is key to maintaining cellular physiology and health. However, during the I/R process of kidney transplantation, mitochondrial function is significantly impaired, resulting in excessive ROS generation [418, 419, 420]. Under ischemic conditions, the increase in the NADH/NAD^+^ ratio within mitochondria and the elevation of membrane potential promote the generation of O_2_•−, further exacerbating oxidative damage [418, 419, 420]. After reperfusion, the restoration of oxygen metabolism stimulates mitochondria to produce more ROS, which not only damages the mitochondrial membrane but may also trigger apoptosis and ferroptosis, creating a vicious cycle [418, 419, 420].

Throughout the process of kidney transplantation, OS is also closely related to immune rejection responses. OS can activate the host immune system, causing it to attack the graft. A substantial amount of ROS can promote the migration and activation of inflammatory cells, enhancing the immune response and triggering acute rejection. Studies have shown that OS activates signaling pathways such as NF‐κB, promoting the release of proinflammatory cytokines, including IL‐1, IL‐6, and TNF‐α, aggravating the rejection response and leading to more severe functional loss following kidney transplantation [421, 422, 423, 424, 425]. Additionally, chronic rejection is also associated with OS. Prolonged OS can lead to the activation of renal stellate cells and collagen deposition, ultimately resulting in fibrosis and the progressive decline of renal function [426].

### 4.6 Metabolic Disorders

#### 4.6.1 Diabetes

OS plays a significant role in the development and progression of diabetes, with mechanisms involving various cellular pathways and biological reactions. Diabetes is a complex metabolic disorder characterized by hyperglycemia, insulin resistance, and insufficient insulin secretion. OS is one of the core mechanisms of diabetes pathogenesis, affecting several critical metabolic processes, such as glucose oxidation, fat metabolism, and the inflammatory response.

Insulin resistance is one of the primary characteristics of diabetes, and OS suppresses insulin signaling through multiple pathways. In a hyperglycemic state, excessive ROS can oxidize IRS, inhibiting their phosphorylation, which directly interferes with the effective transmission of insulin signals [427]. Studies have shown that ROS can also activate phosphatases, subsequently reducing the activity of IRS and affecting the activation of downstream signaling pathways. This mechanism leads to the impaired translocation of glucose transporter type 4, which affects cellular glucose uptake, thereby creating a vicious cycle that exacerbates insulin resistance [427].

OS also enhances insulin resistance through mitochondrial dysfunction, as normal mitochondrial function is crucial for insulin signaling [428, 429]. As OS increases, the functionality of the mitochondrial respiratory chain becomes impaired, leading to insufficient energy production and increased proton leakage. This process further decreases cellular ATP levels, impacting the ability of insulin‐stimulated cells to uptake glucose and increasing the risk of developing insulin resistance [428, 429].

Additionally, OS promotes the formation of insulin resistance through the activation of proinflammatory signaling pathways. ROS can activate NF‐κB and JNK/stress‐activated protein kinase signaling pathways, leading to the serine phosphorylation of IRS‐1 and IRS‐2, which further impairs normal insulin signaling [428, 430, 431]. Research has found a close association between low‐grade chronic inflammation and insulin resistance, suggesting that OS may contribute to this chronic inflammation, where inflammatory mediators such as TNF‐α and IL‐6 exacerbate the pathological state of insulin resistance [428, 431].

Another mechanism involves the dysfunction of pancreatic β‐cells. OS impairs β‐cell function, reducing insulin production and secretion [427, 432, 433]. High concentrations of ROS induce apoptosis in β‐cells, resulting in an increase in apoptotic cells. This process is mediated through multiple proapoptotic signaling pathways, such as affecting the expression of TFs like MafA, which in turn suppresses the transcription of the insulin gene [427, 432‐434]. Furthermore, free radicals damage ATP‐sensitive K channels, and this impairment directly affects insulin secretion capacity, making glycemic control more challenging [427, 432, 433, 435].

Glycolysis is a crucial pathway in carbohydrate metabolism responsible for converting glucose into energy. Under normal physiological conditions, the ROS produced during glycolysis are balanced by antioxidant systems. However, in a hyperglycemic state, the accelerated glycolytic process leads to excessive accumulation of intermediates such as glucose‐6‐phosphate (G6P) and fructose‐6‐phosphate (F6P), which trigger OS [436, 437]. G6P can cause DNA damage by activating PARP‐1; this damage, in turn, exacerbates ROS generation and affects glycolysis [436, 437, 438]. At the same time, the formation of AGEs under high glucose conditions is also closely related to OS, where AGEs induce OS through their receptors, further damaging cellular function [436, 437].

##### 4.6.1.1 Role of RNS in Diabetes

Chronic overproduction of RNS, notably ONOO⁻, plays a critical role in the pathogenesis of diabetes by inducing mitochondrial dysfunction and OS, particularly in insulin‐sensitive tissues such as skeletal muscle and adipose tissue [318]. Excessive RNS generation impairs the ETC activity within mitochondria, leading to disrupted energy metabolism and increased production of ROS. This mitochondrial dysfunction exacerbates redox imbalance, which contributes to insulin resistance by interfering with key intracellular signaling pathways [318, 439, 440]. ONOO⁻‐mediated nitration of IRS further compromises insulin signaling by modifying tyrosine residues essential for downstream glucose transport processes. Such nitrative modifications inhibit proper IRS function, reducing glucose uptake and exacerbating hyperglycemia [318, 440].

#### 4.6.2 Obesity

Obesity is widely recognized as a global health issue, fundamentally arising from a long‐term energy imbalance, primarily characterized by excessive caloric intake and insufficient energy expenditure. In addition to traditional risk factors for obesity, such as excessive energy consumption and a lack of physical activity, OS also plays a key role in the development of obesity.

OS is significantly involved in the metabolic dysregulation associated with obesity. Under the influence of high‐calorie diets, levels of FFA and glucose in the body significantly increase, and these metabolites affect the redox status of cells through multiple metabolic pathways. Excessive glucose produces a large amount of reducing coenzymes, such as NADH and FADH_2_, via glycolysis and the TCA cycle. These coenzymes lead to electron leakage in the ETC, resulting in the production of superoxide and thereby inducing OS. In addition, excessive glucose can also be converted to sorbitol via the polyol pathway, consuming NADPH and exacerbating ROS generation [441]. In obese individuals, the presence of excess FFA challenges the capacity of mitochondrial energy metabolism, leading to an overflow of electrons in the ETC, causing electron leakage and generating large amounts of ROS. This increase in ROS not only reduces energy generation efficiency but also elevates the level of OS in cells [441, 442]. Furthermore, the excessive accumulation of FFA promotes LPO, a process that causes significant cellular damage. The deposition of FFAs on cell membranes affects membrane integrity and fluidity, leading to cellular dysfunction [441, 442]. Simultaneously, the accumulation of FFA stimulates the release of proinflammatory cytokines, such as TNF‐α and IL‐6, promoting a systemic low‐grade inflammatory response, thereby creating a vicious cycle. Specifically, the presence of FFA activates the NOX pathway in adipose tissue, enhancing ROS production, which exacerbates oxidative damage and affects insulin signaling pathways, leading to increased insulin resistance. Notably, high levels of FFA in tissues such as mitochondria, liver, and muscle are closely correlated with the occurrence of insulin resistance and metabolic syndrome [441, 442].

At the same time, the relationship between OS and inflammatory responses is also extremely close. In the state of obesity, adipose tissue serves not only as an energy reservoir but also as an endocrine organ that releases a large amount of proinflammatory factors, including an increased level of TNF‐α, which further promotes the release of IL‐6. The increase in these factors leads to the development of systemic low‐grade inflammatory responses. The release of proinflammatory factors promotes ROS generation and further activates proinflammatory cellular pathways such as NF‐κB and JNK, creating a vicious cycle [441, 443].

#### 4.6.3 Hyperlipidemia

Hyperlipidemia is defined as a state of abnormally elevated concentrations of lipids (such as cholesterol and triglycerides) in the blood, and it is a promoting factor for many metabolic diseases.

In hyperlipidemia, enhanced OS promotes the occurrence of ferroptosis, affecting the function of cardiac cells and VSMCs. Research indicates that excessive iron accumulation can inhibit the activity of lipoprotein lipase, resulting in abnormal triglyceride accumulation. When lipids are esterified in cell membranes, they become substrates for LPO, further exacerbating the cellular OS response [444]. In a hyperlipidemic environment, the generation of oxLDL increases, a process that not only leads to endothelial dysfunction but also promotes iron‐dependent cell death by inhibiting the antioxidant enzyme GPX4 [445].

OS enhances both endogenous and exogenous inflammatory responses in hyperlipidemia, creating a vicious cycle. On one hand, excessive FFA in a hyperlipidemic state stimulate adipose tissue to produce various proinflammatory factors, triggering systemic chronic low‐grade inflammation. This inflammation activates macrophages and other immune cells, prompting their infiltration into damaged tissues and exacerbating OS. On the other hand, OS itself can also promote the intensification of inflammatory responses. The excessive production of ROS not only stimulates endothelial cells to release inflammatory factors but also exacerbates the atherosclerotic process by upregulating the formation of oxLDL. Furthermore, oxLDL activates the NLRP3 inflammasome, initiating inflammatory signaling pathways that lead to the release of inflammatory factors such as IL‐1β, further promoting systemic inflammatory responses [446].

#### 4.6.4 Nonalcoholic Fatty Liver Disease

Nonalcoholic fatty liver disease (NAFLD) is characterized by the abnormal accumulation of fat in the liver, unrelated to alcohol consumption, and is closely associated with obesity, diabetes, and metabolic syndrome. In recent years, OS has garnered widespread attention in the development and progression of NAFLD and has become a key aspect of its pathological mechanism research.

Mitochondria play a crucial role in NAFLD. As the primary energy suppliers of cells, mitochondrial dysfunction is closely linked to the dysregulation of hepatic fat metabolism [447, 448]. In patients with NAFLD, mitochondria often exhibit ultrastructural damage and mtDNA damage. These impairments reduce the activity of respiratory chain complexes, leading to decreased ATP synthesis and an accompanying increase in ROS production. Within mitochondria, the β‐oxidation of fatty acids is a vital step for energy generation; however, excess ROS can directly damage mitochondrial membranes, triggering dysregulation of oxidative phosphorylation. This dysregulation not only affects ATP synthesis but also increases the formation of lipid peroxides, leading to further oxidative damage and diminished cellular function. In this process, mitochondria increasingly become a source of OS, creating a vicious cycle that ultimately exacerbates the progression of NAFLD [447, 448].

Fatty acid metabolism plays a central role in the onset of NAFLD, and OS is critical for the regulation of this process. Under normal circumstances, the liver should maintain a dynamic balance between lipogenesis and fatty acid oxidation. However, in NAFLD, excessive accumulation of FFAs stimulates lipogenesis while simultaneously inhibiting fatty acid oxidation. OS interacts with the regulatory pathways of fatty acid metabolism via ROS, promoting enhanced lipogenesis and suppressed fatty acid oxidation. Elevated levels of ROS not only promote the expression of lipogenic genes but can also inhibit the activity of PPARα and carnitine palmitoyltransferase 1, which are associated with fatty acid oxidation, leading to further accumulation of fatty acids in the liver. This metabolic dysregulation not only affects the energy balance of the liver but also exacerbates the severity of fatty liver [449, 450].

### 4.7 Autoimmune Diseases

Chronic inflammation fueled by ROS creates a self‐amplifying loop across oncologic, metabolic, and autoimmune pathologies. CAFs secrete IL‐6 under oxidative conditions, mirroring adipocyte‐derived proinflammatory cytokines in obesity. The NLRP3 inflammasome serves as a redox‐sensitive hub in all three contexts: promoting tumor metastasis via IL‐1β, impairing insulin signaling in diabetes, and breaking immune tolerance in lupus. Yet, tissue‐specific regulators emerge—hypoxia‐inducible factors dominate in tumors, while leptin–ROS crosstalk prevails in metabolic disorders.

#### 4.7.1 Rheumatoid Arthritis

Rheumatoid arthritis (RA) is a multifactorial autoimmune disease characterized by the destruction of bone and cartilage, as well as synovial proliferation, leading to joint pain, swelling, and stiffness. In the pathogenesis of RA, activated fibroblast‐like synoviocytes exhibit tumor‐like proliferative characteristics, resulting in cartilage erosion and eventual joint destruction. The initial symptoms of RA typically include pain in the fingers and wrists, and as the disease progresses, larger joints such as the shoulders and knees may also be affected, ultimately leading to restricted joint movement, joint deformities, and even disability. Additionally, RA may cause systemic multiorgan damage and extra‐articular manifestations.

In the synovial tissue of RA patients, common ROS include O_2_•−, H_2_O_2_, •OH, and NO. These molecules play a significant role in the pathophysiology of RA, particularly by promoting local inflammatory responses that further exacerbate damage to joints and synovial tissues [451]. Studies have shown that the levels of antioxidants such as GSH and SOD are often reduced in RA patients, leading to a significant increase in OS [451].

In the synovial tissue of RA, activated macrophages and T cells induce the generation of ROS by releasing proinflammatory cytokines such as TNF‐α and IL‐1β. These inflammatory factors not only directly damage synovial cells but also trigger further inflammatory responses by increasing ROS generation. At this stage, a vicious cycle emerges between OS and inflammation, promoting cellular damage and enhancing disease progression [19]. OS also exacerbates local inflammation and contributes to further joint destruction by affecting T cell activation [19, 452].

In the pathogenesis of RA, OS further promotes inflammatory responses and cellular damage by activating specific signaling pathways, such as the MAPK and NF‐κB pathways. The MAPK pathways, including P38, ERK, and JNK, are significantly activated during the inflammatory processes of RA, facilitating the release of proinflammatory cytokines [453, 454, 455]. NF‐κB, as a major regulatory factor in the inflammatory response, is activated under the influence of OS, enhancing the release of IL‐1 and TNF‐α, thereby forming a self‐amplifying feedback loop. With the accumulation of these proinflammatory factors, joint inflammation intensifies, the invasiveness of synovial cells increases, and ultimately leads to joint structural destruction [452].

Patients with RA commonly exhibit endothelial dysfunction, which is manifested in both macrocirculation and microcirculation. Studies have shown a positive correlation between disease activity in early RA patients and microvascular dysfunction. The OS damage to endothelial cells not only affects the integrity of blood vessels but may also lead to the development of comorbidities such as AS and cardiovascular diseases. Research has found that IL‐1 can induce OS even in the preclinical stage of RA, further leading to vascular damage [456].

#### 4.7.2 Systemic Lupus Erythematosus

Systemic lupus erythematosus (SLE) is a unique autoimmune disease characterized by the excessive production of various autoantibodies targeting the cellular nucleus, cytoplasm, and cell membrane. These autoantibodies form immune complexes that deposit in various tissues and organs, leading to tissue damage, where OS plays a key role.

Treg cells are crucial for maintaining immune balance and preventing autoimmune responses. However, in SLE patients, both the quantity and function of Treg cells are significantly reduced. OS influences the differentiation and function of Treg cells through various mechanisms. Forkhead box protein 3 (Foxp3) is a key TF for Treg cell development and function; OS inhibits the expression of Foxp3 by increasing the production of IL‐6, consequently reducing the number of Treg cells. Elevated levels of IL‐6 have been widely documented in SLE patients, suggesting that OS may be one of the primary reasons for the reduction of Treg cells [457, 458, 459, 460]. Additionally, the mTOR signaling pathway plays a negative regulatory role in the differentiation and function of Treg cells. The mTOR complex 1 (mTORC1) and mTORC2 complexes promote the differentiation of T helper 1 cell (Th1) and Th17 cells by inhibiting the expression of Foxp3 and the expansion of Treg cells, further exacerbating immune imbalance [457, 458, 461‐463]. Leptin, a hormone closely related to OS, further weakens Treg cell function by inhibiting Treg cell proliferation and enhancing mTOR activity [464].

Th17 cells are key effector cells in the pathogenesis of SLE, and their amplification is closely related to OS. Ultraviolet (UV) radiation is a significant environmental trigger for SLE, promoting Th17 cell differentiation by inducing DNA damage and the production of proinflammatory cytokines. UV radiation also generates endogenous aryl hydrocarbon receptor (AHR) ligands like 6‐formylindolo[3,2‐b]carbazole, which activate AHR, further promoting the amplification of Th17 cells. AHR serves as a critical regulator of Th17 cell differentiation, and its activation exacerbates the symptoms of SLE [465, 466, 467]. Importantly, the mTOR signaling pathway also plays a significant role; OS activates the mTORC1 signaling pathway, regulating TFs associated with Th17 cells and positively promoting their differentiation, thereby aggravating the progression of SLE [468, 469, 470].

In SLE patients, mitochondrial dysfunction is one of the significant manifestations of OS, directly affecting immune cell function. The high‐energy state of mitochondria induced by OS results in increased mitochondrial potential and insufficient ATP synthesis, leading to increased ROS production and further intensifying the inflammatory response. Lymphocytes from SLE patients are more prone to apoptosis under OS, resulting in a reduction in lymphocyte numbers, which further exacerbates immune imbalance [471].

OS affects T cell function through various mechanisms, including the alteration of cytokine expression, regulation of gene transcription, and changes in mitochondrial function. OS promotes the production of TH2 cytokines and suppresses the expression of Th1 cytokines, leading to an imbalance in T cell differentiation [472, 473]. GSH deficiency and increased mitochondrial calcium storage exacerbate this imbalance. At the same time, OS alters T cell signaling pathways and lineage development by affecting DNA methylation and histone modification [474].

#### 4.7.3 Psoriasis

Psoriasis is a chronic immune‐mediated skin disease characterized by the appearance of red patches covered with silvery scales on the skin. The pathophysiological features of this disease indicate that OS plays a significant role in the development and progression of psoriasis. Studies have shown that the exacerbation of OS is not only associated with the severity of skin lesions but may also impact the effectiveness of treatments.

Research demonstrates that OS markers such as MDA and NO levels are significantly elevated in the blood and skin of psoriatic patients, indicating an increased extent of LPO and protein oxidation. Concurrently, levels of antioxidants such as GSH and vitamin E are generally reduced, reflecting a weakened antioxidant capacity. Additionally, the activity of antioxidant enzymes, including SOD and CAT, is decreased, further indicating dysregulation of the antioxidant system [475, 476].

DCs play a critical role in the pathogenesis of psoriasis. OS enhances the production of proinflammatory cytokines such as IL‐8 and TNF‐α by DCs, promoting their interaction with T cells. The functional enhancement of these inflammatory DCs drives the immunopathological processes associated with the onset of psoriasis [477, 478]. In psoriasis, Th1‐type cytokines such as IFN‐γ and IL‐2 are significantly increased, while the levels of Th2‐type cytokines like IL‐10 are relatively low, further promoting the immune response related to psoriasis [478]. Meanwhile, OS also promotes the abnormal proliferation of KCs. The overexpression of cytokines such as TNF‐α stimulates the proliferation and differentiation of KCs, leading to impaired skin barrier function and characteristic skin lesions [478, 479, 480, 481]. Moreover, OS is closely related to endothelial cells and angiogenesis in psoriatic patients. ROS promote vascular formation through a VEGF‐mediated pathway, which further exacerbates the local inflammatory environment in psoriasis [482].

OS plays a role in the pathogenesis of psoriasis by activating multiple signaling pathways. Studies indicate that ROS can activate signaling pathways such as MAPK and NF‐κB. The abnormal activation of ERK, JNK, and p38 within the MAPK pathway is closely associated with the excessive proliferation and inflammation of psoriatic lesions. In particular, the activation of p38–MAPK during the pathological process may worsen the inflammatory state [20, 483, 484]. NF‐κB, as a key TF, has significantly elevated activation levels in psoriatic lesions, enhancing the expression of proinflammatory genes and perpetuating inflammation [20, 485]. Furthermore, OS triggers the activation of the JAK–STAT signaling pathway, wherein the upregulation of STAT3 plays an significant role in the immunopathology of psoriasis. The abnormal activation of these signaling pathways not only exacerbates inflammatory responses but also promotes the proliferation and differentiation of KCs [486, 487].

### 4.8 Musculoskeletal Diseases

#### 4.8.1 Osteoarthritis

Osteoarthritis (OA) is a prevalent joint disease increasingly recognized as a significant health problem, particularly among the elderly population. It is characterized by swelling and pain in the affected joints, often accompanied by restricted mobility and disability. OS is a critical contributor to the progression of OA, with studies indicating that OS levels in damaged joint tissue are significantly higher than those in healthy joints, thereby exacerbating injury and functional impairment in the joints.

In the pathological processes of OA, OS is closely linked to the production of various inflammatory factors, especially IL‐1β and TNF‐α, which play pivotal roles in the inflammatory response. ROS can induce and amplify the production of these proinflammatory cytokines, creating a vicious cycle that leads to chronic joint inflammation. Specifically, ROS enhance the expression of matrix‐degrading proteins, inhibit the synthesis of the ECM, and elevate the risk of apoptosis or necrosis in chondrocytes, thereby worsening cartilage damage and functional impairment [488, 489]. Research has demonstrated increased expression levels of pro‐oxidative enzymes, such as NOX2, in the synovium of OA patients, which correlates with collagen metabolism. Notably, the upregulation of NOX2 contributes to collagen degradation by generating ROS, adversely affecting the structural integrity of the joints [488, 489].

Furthermore, OS in the context of OA not only promotes cell apoptosis but also encompasses other regulated forms of cell death, such as ferroptosis. The importance of ferroptosis in OA is being increasingly acknowledged, characterized primarily by excess iron accumulation and LPO. In OA chondrocytes, iron accumulation exacerbates oxidative damage by inducing ROS production. The downregulation of the GPX4 enzyme is closely associated with GSH depletion, leading to diminished cellular resistance to OS and resulting in cell death [489, 490]. The occurrence of ferroptosis further contributes to the degradation of the ECM, facilitating the pathological progression of OA [490]. Additionally, OS promotes apoptosis, which can occur via intrinsic and extrinsic pathways; ROS can activate both routes. In the intrinsic pathway, ROS induce proapoptotic factors, such as Bak and Bax, through the activation of cell cycle regulators like p53 and JNK, leading to increased mitochondrial membrane permeability and subsequent apoptosis. Low ROS levels may induce cell cycle arrest to allow for DNA repair. The extrinsic pathway is activated through transmembrane death receptors like Fas and TNF‐related apoptosis‐inducing ligand receptors. The ROS produced interact with the intrinsic apoptotic processes, thereby enhancing the extrinsic signaling [489, 490, 491, 492].

In OA, ROS presence modulates several signaling pathways, such as the activation of the MAPK and NF‐κB pathways. OS initiates the phosphorylation of MAPK, which promotes NF‐κB activation, consequently increasing the expression of various proinflammatory cytokines. The activation of these signaling pathways not only fuels the inflammatory response in OA but also influences cell survival and metabolism [493, 494].

#### 4.8.2 Osteoporosis

Osteoporosis is an age‐related condition with increasing incidence among the elderly, significantly elevating the risk of fractures. Maintaining the integrity and homeostasis of bone tissue relies on a balance between osteoblasts and osteoclasts. OS is crucial in the development of osteoporosis, as excessive ROS can damage bone cells, affecting both bone resorption and formation, and thus accelerating the progression of the disease.

Mitochondria are critical energy factories within cells and play an essential role in energy metabolism. As individuals age, mitochondrial dysfunction leads to increased ROS production. mtDNA, lacking the protective mechanisms present in nuclear DNA, is particularly vulnerable to oxidative damage and is closely associated with cellular aging. OS damages mitochondrial function and disrupts ATP synthesis, diminishes mitochondrial membrane potential, and alters calcium homeostasis, adversely impacting osteoblast functionality. Additionally, OS influences cellular metabolism by affecting mitochondrial dynamics, including fission and fusion processes. Under conditions of OS, bone mesenchymal stem cells (BMSCs) can transfer intact mitochondria to damaged cells; however, in osteoporosis, the migration of oxidatively damaged mitochondria may negatively affect cellular function [495, 496].

BMSCs are integral to bone metabolism, but their behavior significantly shifts under OS. For instance, AGEs have been shown to inhibit the osteogenic potential of BMSCs while promoting their differentiation into adipocytes. This negative impact can be partially alleviated through the regulation of mitochondrial autophagy [497]. Furthermore, Sirt3, an NAD^+^‐dependent enzyme, can protect the osteogenic function of BMSCs by activating mitochondrial autophagy, thereby slowing the aging process. Sirt3 overexpression has demonstrated an inhibitory effect on osteoporosis in mouse models, highlighting its protective role in the OS response of bone cells [498].

OS impacts bone metabolism via multiple mechanisms, particularly by disrupting the balance between osteoblasts and osteoclasts. Research reveals that in osteoporosis, the accumulation of ROS decreases nuclear translocation of the Nrf2 TF, thereby reducing the expression of antioxidant genes, which promotes osteoclastogenesis and aggravates osteoporosis. Specifically, H_2_O_2_ stimulates the differentiation of osteoclasts while reducing Nrf2 expression through the activation of the NF‐κB signaling pathway [499]. In another critical aspect of bone metabolism, OS enhances bone resorption by increasing osteoclast formation. This occurs due to OS‐induced upregulation of receptor activator of nuclear factor kappa‐B ligand (RANKL) and downregulation of osteoprotegerin (OPG); the rise in RANKL activates osteoclasts, while the decrease in OPG leads to reduced inhibitory control over osteoclast development. Chronic OS and inflammation can significantly accelerate osteoclast formation, resulting in bone loss [499, 500, 501].

Additionally, OS affects bone density by promoting apoptosis in bone cells. Excessive ROS have been shown to induce permanent cell cycle arrest and apoptosis in osteoblasts, worsening osteoporosis. In patients with osteoporosis, the apoptosis of bone cells not only increases osteoclast generation by releasing RANKL but also disrupts osteogenic function by inhibiting the Wnt/β‐catenin signaling pathway [499, 502, 503]. Finally, excessive iron accumulation and OS can trigger ferroptosis in osteoblasts, a process characterized by LPO and the inactivation of antioxidant enzymes such as GPX4 [499].

#### 4.8.3 Sarcopenia

Sarcopenia is primarily characterized by a decrease in skeletal muscle mass and a decline in muscle function. Primary sarcopenia is mainly age‐related, whereas secondary sarcopenia arises due to conditions such as heart failure, kidney failure, malignancies, and chronic obstructive pulmonary disease.

In elderly muscle cells, the accumulation of OS is closely linked to a diminished antioxidant and repair system. As antioxidant defenses weaken, oxidized molecules more readily accumulate within muscle cells, resulting in a loss of the cells' capability to respond to OS. This may be a critical factor in the inadequate response of muscle tissue to oxidative challenges [504, 505]. Additionally, excessive production of reactive oxygen and nitrogen species (RONS), combined with a reduced responsiveness of the antioxidant system, can contribute to the onset of sarcopenia and hinder muscle regeneration. During the pursuit of muscle function recovery, OS not only impedes the regenerative capacity of muscle cells but may also activate the ubiquitin–proteasome system, leading to accelerated muscle atrophy. This system is typically activated in muscle cells, enhancing the degradation of damaged proteins and further diminishing muscle quality and functionality [504, 505].

The high energy demands of muscle contraction lead to substantial production of RONS. For instance, during muscle contraction, mitochondria produce free radicals such as superoxide anions. Moderate levels of RONS stimulate cells to undergo adaptive responses by activating TFs that regulate the expression of antioxidant enzyme genes, thus safeguarding muscle cells against oxidative damage. However, an imbalance between the generation and elimination of RONS can lead to OS, which significantly accelerates the progression of sarcopenia [506, 507]. NOX is one of the principal oxidases in skeletal muscle cells, found in the plasma membrane and transverse tubules, catalyzing the conversion of NADPH to NADP⁺ while releasing superoxide anions. The O_2_•− generated by this enzyme during muscle contraction modulates redox signaling, maintaining cellular vitality. However, dysregulation of this signaling may contribute to critical pathways associated with muscle atrophy [506, 507, 508].

Mitochondria serve as the main energy producers within cells and are also a key source of oxidants. The susceptibility of mtDNA to oxidative damage, coupled with ineffective repair mechanisms, leads to the accumulation of mtDNA mutations during aging, creating a detrimental cycle that impairs functionality. According to the mitochondrial free radical theory of aging, oxidative damage‐induced mitochondrial dysfunction triggers the synthesis of defective ETCs, hampers oxidative phosphorylation, and reduces ATP production, resulting in enhanced ROS generation [509, 510, 511]. Evidence indicates a strong correlation between mtDNA damage and muscle atrophy in mouse models, signifying the central role of mitochondrial dysfunction in age‐related sarcopenia [509, 512].

In sarcopenia, OS leads to iron overload in muscle cells, further aggravating cellular damage. LPO resulting from iron overload increases intracellular ROS production and initiates ferroptosis through the p53/SLC7A11 pathway [513]. Research has shown that iron overload reduces phosphorylation levels of FOXO3a and Akt while increasing the expression of E3 ubiquitin ligases associated with muscle atrophy, such as muscle RING‐finger 1 and atrogin‐1 [514, 515, 516, 517]. Moreover, the role of macrophages in muscle regeneration is significant. These immune cells facilitate muscle repair by expressing CD163, ferritin, and HO‐1 to absorb and store iron. The release of iron by macrophages promotes muscle regeneration, while inhibiting macrophage iron output can diminish regenerative capacity and enhance fat accumulation [514, 515].

### 4.9 Retinal Disease

#### 4.9.1 Glaucoma

Glaucoma is a chronic ocular disease characterized by optic nerve damage and loss of visual fields, often linked to elevated intraocular pressure (IOP). Recent research has demonstrated that OS plays a critical role in the onset and progression of glaucoma. OS is caused by the excessive generation of ROS and a failure of antioxidant defense mechanisms, leading to damage in various cell types, including ganglion cells and retinal pigment epithelial (RPE) cells.

The trabecular meshwork (TM) is a crucial structure for maintaining stable IOP by regulating the outflow of aqueous humor. In primary open‐angle glaucoma, TM cells undergo pathological changes that increase outflow resistance and elevate IOP. This elevation results in pressure on the optic nerve head, promoting the death of retinal ganglion cells (RGCs) and subsequent optic nerve damage. OS exacerbates this pathological process by impairing the normal function of TM cells [518, 519]. ROS can diminish the effectiveness of local antioxidants, enhance aqueous humor outflow resistance, and contribute to raised IOP. Studies have shown that TM cells exhibit high sensitivity to OS, leading to lysosomal dysfunction and disruptions in autophagy, which promote cellular senescence and compromise TM function. In TM cells from glaucoma patients, matrix accumulation and inflammatory responses have been observed, further intensifying oxidative damage. Increased ROS levels can affect the adhesion and integrity of TM cells, leading to blockages in aqueous outflow pathways [518, 519, 520, 521].

OS results in mitochondrial dysfunction, which is essential for the survival of RGCs. Both increases in intracellular ROS and the accumulation of mtDNA mutations can trigger apoptosis or necrosis, particularly in RGCs, which are especially vulnerable due to their high metabolic demands. Thus, OS not only directly affects cell survival but also further impairs visual transduction functions [522, 523].

Recent studies have identified that miRNAs play significant roles in the changes of ECM synthesis in TM cells induced by OS [518]. miR‐29b has been implicated in conferring protective effects under chronic OS and physiological oxygen levels. In normal conditions, miR‐29b negatively regulates the expression of key collagens (such as collagen type I alpha 1 chain [COL1A1] and COL1A2) involved in the synthesis and deposition of the ECM in TM cells. However, under chronic OS conditions, the downregulation of miR‐29b results in increased expression of these genes, leading to cytotoxic effects [524, 525, 526]. Additionally, other miRNAs, including miR‐141 and miR‐93, have been shown to have close associations with OS in the pathophysiology of glaucoma. miR‐141 decreases OS by activating the Nrf2 signaling pathway, while miR‐93 inhibits Nrf2, thus promoting apoptosis in RGCs. These findings suggest that the modulation of miRNAs could represent a potential therapeutic approach for glaucoma [527, 528].

Exosomes produced by nonpigmented ciliary epithelial cells (NPCE) are crucial for maintaining TM function and regulating IOP in response to OS. These exosomes can support TM cell metabolism and mitigate oxidative damage, helping to disrupt the vicious cycle between OS and TM dysfunction. Notably, under OS, NPCE‐derived exosomes can upregulate the Wnt/β‐catenin signaling pathway, which participates in cell proliferation and apoptosis [529, 530]. Studies have revealed that exosomes are rich in proteins that participate in ECM remodeling, influencing the composition of the ECM by regulating the activity of MMPs, thereby reducing resistance to aqueous humor outflow [529].

#### 4.9.2 Diabetic Retinopathy

Diabetic retinopathy (DR) is one of the most common complications among diabetic patients, primarily characterized by retinal vascular lesions and optic nerve damage. Increasing evidence indicates that OS plays a vital role in the onset and progression of DR.

In diabetic patients, mtDNA is subjected to OS, resulting in epigenetic changes. mtDNA is more susceptible to free radical attacks in the diabetic environment due to ineffective protective mechanisms, leading to damage in specific regions, particularly the displacement loop (D‐loop). This D‐loop contains crucial sequences for regulating mtDNA replication and transcription; damages and increases in mutations in this region directly disrupt normal mitochondrial function [531, 532]. Impaired mtDNA not only diminishes the ability of mitochondria to synthesize ATP but also affects the ETC, consequently leading to excessive ROS production. Moreover, mtDNA damage can result in dysregulation of cellular signaling, triggering inflammatory responses and apoptosis, thus worsening pathological changes in the retina.

Mitochondria are essential for cellular energy metabolism, and their functionality is significantly compromised in diabetic conditions. Continuous hyperglycemia triggers mitochondria to produce excessive ROS, which damages mitochondrial membranes and alters membrane permeability. This alteration enables the release of proapoptotic factors, such as cytochrome *c*, from the mitochondria into the cytoplasm, activating the caspase cascade and ultimately initiating apoptosis [533, 534]. During this process, the activity of antioxidant enzymes, such as SOD and GPX, is significantly reduced, leading to diminished antioxidant capacity and exacerbating oxidative damage to cells. Therefore, mitochondrial dysfunction not only impacts the survival of retinal cells directly but also affects overall visual function, resulting in persistent damage to retinal neurons.

In the context of hyperglycemia, various metabolic pathways are activated, leading to the overproduction of ROS and the promotion of OS [535]. Under high glucose conditions, the polyol pathway converts glucose to sorbitol via aldose reductase, which is subsequently oxidized to fructose. This process consumes NADPH, leading to reduced NADPH levels. NADPH is a precursor for the synthesis of reduced GSH, and its reduction significantly decreases the antioxidant capabilities of cell, making cells more susceptible to oxidative damage. Additionally, the accumulation of sorbitol and fructose raises intracellular osmotic pressure, resulting in cellular edema and membrane permeability damage, which further drives the development of retinal pathology [536, 537, 538, 539]. In the hexosamine pathway, glucose is transformed into F6P. Activation of this pathway in hyperglycemic conditions increases ROS production and inhibits glyceraldehyde‐3‐phosphate dehydrogenase activity, leading to the overflow of its metabolites into the hexosamine pathway. This process not only increases H_2_O_2_ production but also promotes changes in endothelial cells, enhancing microvascular permeability and subsequently affecting the normal structure and function of the retina [540, 541, 542]. In the PKC pathway, high glucose states activate this pathway through the synthesis of ROS and diacylglycerol, causing the upregulation of PKC isoforms such as PKC‐α, PKC‐β, PKC‐δ, and PKC‐ε. These activated PKC isoforms facilitate changes in endothelial cell permeability and induce the expression of VEGF, promoting angiogenesis, endothelial cell damage, and pericyte loss, thereby accelerating the progression of DR [543, 544, 545, 546].

In DR, the activation of multiple signaling pathways significantly influences OS, particularly the NF‐κB and NOX pathways. NF‐κB, a key TF in OS, is activated under hyperglycemic conditions, promoting the expression of inflammatory factors such as TNF‐α and IL‐6, which further exacerbate inflammatory responses in the retina. The persistent activation of NF‐κB not only facilitates apoptosis but also inhibits the expression of antioxidant enzymes, leading to prolonged exposure of retinal cells to an oxidative environment, thereby worsening pathological changes in the retina [547, 548]. The activation of the NOX complex also plays a significant role in DR. One key function of NOX is to generate ROS, and its excessive activation under diabetic conditions leads to increased OS, interacting with the activation of other signaling pathways, such as MMPs. This interaction exacerbates further intracellular damage, affecting retinal microvascular function and strongly correlating with the worsening of retinal pathology [351, 549, 550].

#### 4.9.3 Age‐Related Macular Mediated Degeneration

Age‐related macular degeneration (AMD) is the most prevalent retinal degenerative disease among the elderly, primarily characterized by the gradual loss of central vision. Recent studies have established that OS is crucial in the onset and progression of AMD. OS results from the excessive production of ROS and a disruption in antioxidant defense mechanisms, leading to detrimental effects on RPE cells and photoreceptor cells.

RPE cells play a vital role in the retina; they maintain the integrity of the blood‐retinal barrier and are responsible for phagocytosing shed outer segments of photoreceptors. In this process, the high metabolic activity and oxygen demands of RPE cells generate significant amounts of ROS, increasing their susceptibility to oxidative damage. In high oxygen tension environments, such as the macular region, OS in RPE cells significantly escalates, further contributing to AMD pathogenesis [551, 552, 553]. As OS intensifies, oxidative damage to proteins, lipids, and mtDNA occurs within RPE cells. Research has identified that ROS accumulation is closely linked to cell death pathways; low levels of ROS may trigger apoptosis, while high levels can induce necrosis. This dysfunction in RPE cells subsequently leads to degenerative changes in the macula, promoting the development of AMD [553, 554].

Autophagy in RPE cells effectively eliminates oxidatively damaged proteins and organelles, thereby mitigating OS. However, when autophagy is suppressed, oxidatively damaged proteins and organelles cannot be adequately cleared, exacerbating OS and facilitating the progression of AMD [555, 556, 557, 558]. Studies suggest that the Nrf2 and PGC‐1 pathways are pivotal in regulating autophagy and combating oxidative damage [554]. Nrf2 serves as a key TF that allows cells to respond to OS by activating the expression of various antioxidant and detoxification genes, including HO‐1 and NQO1. Under normal conditions, Nrf2 forms a complex with Keap1, which targets Nrf2 for degradation. However, under OS, conformational changes in Keap1 inhibit Nrf2 degradation, allowing it to translocate to the nucleus to activate antioxidant responses. Nrf2 enhances the antioxidant capacity of cells and also contributes to cellular resistance to oxidative damage by influencing autophagy and the ubiquitin–proteasome system [559, 560]. Concurrently, PGC‐1α, a critical factor in regulating mitochondrial biogenesis, bolsters RPE cells’ antioxidant defenses by inducing genes involved in oxidative metabolism and antioxidant enzymes. Evidence suggests a cooperative interaction between PGC‐1α and Nrf2, as both promote the expression of antioxidant enzymes and enhance cellular responses to OS. Additionally, the activation of Nrf2 and PGC‐1α can boost autophagic activity, reduce intracellular protein aggregation, and improve the cellular protective mechanisms [561, 562].

OS not only directly damages RPE cells but also induces inflammatory responses, particularly through the activation of the NLRP3 inflammasome. Elevated levels of ROS stimulate the release of proinflammatory factors such as IL‐1β and TNF‐α, which contribute to the pathological progression of AMD. There is a bidirectional relationship between inflammation and OS, as inflammatory responses can further exacerbate OS, leading to a vicious cycle that intensifies damage to RPE cells and photoreceptor cells [557].

### 4.10 Reproductive System Disease

#### 4.10.1 Male Infertility

Male infertility represents a growing public health concern. Substantial evidence indicates that OS plays a pivotal role in the pathogenesis of male infertility by inducing oxidative damage to germ cells and reproductive tissues, ultimately impairing spermatogenesis and semen quality.

ROS generated during OS encompass O_2_•−, H_2_O_2_, and •OH. Human spermatozoa exhibit particular vulnerability to OS owing to their plasma membrane composition rich in PUFAs and limited endogenous antioxidant defenses [563, 564]. This makes sperm vulnerable to lipid, protein, and DNA damage when ROS is excessive, which in turn affects sperm motility, function, and fertilization ability [564, 565]. This biological predisposition renders sperm susceptible to ROS‐mediated damage through three primary mechanisms: (1) LPO of membrane PUFAs compromises membrane integrity, impairing sperm motility and viability [566, 567]; (2) DNA fragmentation and oxidative base modifications jeopardize genetic integrity, reducing fertilization potential and increasing risks of embryonic abnormalities [567, 568, 569, 570]; (3) protein carbonylation disrupts structural and functional proteins essential for motility and chromatin packaging [567]. Mitochondrial dysfunction exacerbates these effects, as oxidative damage to ETC components reduces ATP synthesis critical for sperm motility and survival [571, 572].

#### 4.10.2 Female Infertility

OS constitutes as key pathogenic factor in female infertility through multifaceted impacts on reproductive physiology.

Ovarian follicles, comprising oocytes and associated granulosa cells, undergo ROS‐mediated regulation during maturation [573, 574, 575]. Physiological ROS levels derived from follicular fluid components (macrophages, leukocytes, and paracrine factors) facilitate luteinizing hormone (LH) surge‐induced ovulation and oocyte maturation [573, 574, 575]. This redox balance demonstrates dual regulatory roles: physiological ROS concentrations support folliculogenesis through steroidogenesis modulation and cytokine signaling, while excessive ROS induces membrane LPO that compromises oocyte developmental competence [573, 574, 575].

The peri‐ovulatory phase exhibits characteristic ROS fluctuations, with pre‐ovulatory follicles demonstrating elevated ROS levels that facilitate follicle rupture and oocyte release [573, 574, 575]. While LH surge‐associated ROS elevation is physiologically essential, supraphysiological levels impair oocyte quality through oxidative damage mechanisms. Persistent ROS accumulation in unruptured follicles may precipitate apoptotic pathways, adversely affecting subsequent cycle outcomes [573, 574, 575].

In polycystic ovary syndrome, a prevalent endocrinopathy characterized by hyperandrogenism, oligo‐ovulation, and polycystic ovarian morphology, OS exacerbates ovarian dysfunction through multiple pathways [575]. Insulin resistance‐driven ROS overproduction synergizes with antioxidant depletion to establish a pro‐oxidant microenvironment. Concurrent chronic inflammation, mediated by TNF‐α and IL‐6 overexpression, further disrupts follicular development through paracrine mechanisms, ultimately contributing to anovulation and infertility [575].

## 5 Strategies for Treating Human Disease

### 5.1 SOD and SOD Mimetics

As a critical intracellular antioxidant enzyme, SOD effectively scavenges O_2_•−, mitigating OS‐induced cellular damage. OS demonstrates strong pathophysiological associations with various human diseases, including cardiovascular disorders, neurodegenerative diseases, diabetes mellitus, and malignancies.

SOD protects endothelial function by catalyzing superoxide anion dismutation, thereby reducing peri‐endothelial OS [576]. Experimental evidence indicates capacity of SOD to inhibit LDL oxidation, a pivotal process in atherogenesis [577]. In myocardial IRI models, exogenous SOD supplementation significantly improves cardiac function, attenuates cardiomyocyte apoptosis, and reduces cardiovascular event rates. The SOD‐M3 isoform demonstrates superior enzymatic activity, cardioprotective properties, and structural stability, positioning it as a promising therapeutic candidate for ischemic heart disease [578]. Enhanced SOD concentrations improve ROS clearance during ischemic episodes, counteracting oxidative damage. Site‐directed modifications of SOD‐M3 optimize its solubility and stability in physiological environments, with enzymatic activity being crucial for therapeutic efficacy [578]. Melatonin exerts cardioprotection by upregulating Sirt3 expression in cardiomyocytes, subsequently enhancing MnSOD activity to alleviate IRI [579]. These findings suggest that exogenous antioxidants (e.g., SOD, nicotinamide riboside) mitigate mtROS production via Sirt3‐SOD2 signaling pathways, suppressing apoptosis and enhancing cardiac ischemic tolerance [580]

SOD and its mimetics represent promising therapeutic targets for neurodegenerative conditions. In AD, zinc/copper supplementation enhances Cu/Zn‐SOD activity, alleviating oxidative neuronal damage. MnSOD mimetics reverse Aβ oligomerization, while MnSOD haploinsufficiency exacerbates cerebrovascular amyloidosis and tau phosphorylation at Ser‐396 in transgenic AD models through mitochondrial OS potentiation [581, 582, 583]. For PD, localized administration of MnSOD mimetics reduces dopaminergic neuron loss and improves motor function while minimizing systemic side effects. Therapeutic strategies include upregulating endogenous SOD synthesis to protect DA‐producing neurons [[Link], [Link]]. In amyotrophic lateral sclerosis, pharmacological enhancement of mutant Cu/Zn‐SOD activity combined with antioxidant supplementation (vitamin E, GSH) alleviates oxidative damage and improves motor neuron survival. Gene‐editing approaches to correct SOD1 mutations show potential in reducing ROS‐mediated apoptosis [586, 587].

Hyperglycemia‐induced OS contributes to diabetic microvascular complications. SOD supplementation preserves pancreatic β‐cell function by converting superoxide radicals to H_2_O_2_, reducing intracellular oxidative burden. Gene therapy‐mediated SOD overexpression enhances β‐cell antioxidant capacity and insulin secretion. Novel MnSOD mimetics replicate native enzyme functions, demonstrating therapeutic potential in diabetes management. In streptozotocin‐induced type 1 diabetes mellitus models, MnSOD‐overexpressing β‐cells improve glycemic control through NF‐κB inhibition and ROS scavenging [588, 589].

MnSOD downregulation in malignancies elevates ROS levels, promoting tumor proliferation and metastasis. SOD activation reverses neoplastic phenotypes, particularly in multiple myeloma, by restoring redox homeostasis. MnSOD mediates apoptosis/autophagy signaling and influences chemoradiation resistance through metabolic reprogramming, favoring glycolytic energy production in cancer cells [590, 591]. In murine breast cancer models, MnSOD overexpression exerts antitumor effects by modulating immune cell infiltration and tumor microenvironment dynamics. Posttranslational modifications (e.g., lysine acetylation) regulate roles of MnSOD in cancer metabolism and immune evasion [591]. SOD mimetics upregulate MnSOD expression and activate AMPK signaling, suggesting novel therapeutic strategies targeting metabolic plasticity in malignancies [592].

### 5.2 GSH‐Px Mimetics

GPX is a significant antioxidant enzyme capable of detoxifying intracellular oxidants by catalyzing the reduction of H_2_O_2_ and organic peroxides. Among the GPX family, GPX1 and GPX4 are major focuses of research, playing critical roles in various disease progressions and providing potential targets for the development of new therapeutic strategies.

In metabolic diseases such as insulin resistance and obesity, the expression level of GPX1 has a significant impact on insulin signaling pathways. Research has shown that excessive levels of GPX1 in mouse models can lead to insulin resistance and hyperinsulinemia, primarily due to the inhibition of normal insulin‐mediated Akt signaling. In this context, GPX1 reduces intracellular levels of ROS, impairing mitochondrial function and ATP production. Conversely, the absence of GPX1 enhances cellular sensitivity to insulin, as indicated by increased insulin‐mediated ROS production and activation of Akt signaling under a high‐fat diet [593]. This finding suggests that, under certain conditions, elevated ROS levels may actually improve insulin sensitivity. Therefore, adjusting the expression of GPX1 and its antioxidant activity may present a novel strategy for preventing and treating type 2 diabetes and metabolic syndrome.

In IRI, GPX4 is a critical regulator of ferroptosis. Studies have indicated that IRI leads to a downregulation of GPX4 expression, increasing cellular sensitivity to ferroptosis. In the kidneys, myocardium, and nervous system, decreased GPX4 levels correlate closely with ROS accumulation, LPO, and mitochondrial dysfunction. In renal tubular epithelial cells, the reduction of GPX4 promotes ferroptosis, contributing to acute kidney failure [594, 595, 596]. In the myocardium, silencing GPX4 results in elevated ROS levels and drives the expression of acyl‐CoA synthetase long‐chain family member 4, thereby triggering ER stress and LPO. Conversely, upregulation of GPX4 has been shown to work in tandem with Hsp60/10 to limit cytochrome *c* release, thus minimizing mitochondrial damage induced by IRI. Additionally, overexpression of GPX4 can protect mitochondrial phospholipid bilayers from oxidative damage and enhance the functionality of the ETC complexes I, III, and IV, resulting in improved mitochondrial efficacy and cardiac contractile performance when GPX4 levels are elevated [597, 598, 599, 600, 601]. In the nervous system, persistent GPX4 reduction within 24 h postcerebral hemorrhage triggers a secondary inflammatory response, which increases BBB permeability and exacerbates brain edema, neuroconductive dysfunction, and neuronal death [602, 603, 604]. Therefore, enhancing the expression or activity of GPX4 is regarded as an effective approach to mitigate cell damage from IRI and protect organ function.

In neurodegenerative diseases, particularly AD and PD, the regulation of GPX4 expression is crucial for preventing neuronal ferroptosis. Research indicates that downregulation of GPX4 in AD models leads to the accumulation of LPO products and the death of hippocampal neurons [605, 606]. Pharmacological agents that promote GPX4 expression, such as the PPARα agonist GW7647, have been shown to decrease β‐amyloid burden and exert neuroprotective effects [607]. Furthermore, supplementation with docosahexaenoic acid (DHA) enhances neuronal function and reduces oxidative damage to hippocampal cells by increasing GPX4 transcription [608, 609].

GPX4 plays a complex role in cancer development. Tumor cells with a high burden of RAS mutations often exhibit sensitivity to GPX4‐mediated ferroptosis. Research indicates that pharmacological interventions can block the activation of the Nrf2/GPX4 signaling pathway, thereby enhancing the sensitivity of tumor cells to ferroptosis. For example, in lung adenocarcinoma, dual knockout of Nrf2 and GPX4 significantly raises tumor cell mortality rates. Additionally, in triple‐negative breast cancer and gliomas, high GPX4 expression correlates closely with increased cell proliferation and metastasis. GPX4 also influences the tumor microenvironment by affecting immune responses and angiogenesis, thus modulating tumor growth [610, 611, 612, 613]. Studies reveal that T cells lacking GPX4 exhibit limited functionality and increased susceptibility to ferroptosis, while upregulation of GPX4 helps maintain T cell function and stability. In addition, GPX4 affects macrophage polarization, promoting the conversion from protumor M2 macrophages to tumor‐suppressing M1 macrophages, thereby enhancing antitumor immune responses [67, 69, 614, 615].

Moreover, GPX mimetics have shown therapeutic potential in various studies. By modulating GPX activity, these mimetics can have roles in treating cancer, cardiovascular diseases, and neurodegenerative disorders, helping to reduce cellular damage and improve tissue function.

### 5.3 Iron Chelation

Iron, an essential trace element, participates in oxygen transport, cellular energy production, and DNA synthesis. However, excessive iron accumulation induces OS through Fenton reaction‐mediated hydroxyl radical generation, causing cellular damage, inflammatory responses, and biomolecule oxidation.

Under physiological conditions, iron metabolism is tightly regulated by ferritin and transferrin. Dysregulated iron homeostasis promotes free iron‐catalyzed ROS overproduction, perpetuating oxidative damage to cellular membranes, proteins, and DNA—mechanisms implicated in cardiovascular, neurodegenerative, metabolic, hepatic, and oncological pathologies [616]. Iron chelators (deferoxamine, deferasirox, deferiprone) mitigate iron‐induced OS by forming stable complexes with labile iron, reducing its bioavailability while modulating inflammatory signaling and apoptosis pathways [616].

Neoplastic cells exhibit iron addiction to sustain proliferation and bioenergetic demands. Tumorigenesis is potentiated by iron overload‐induced ROS generation [617], with malignant cells upregulating TFRs and siderophores to enhance iron uptake [618, 619]. Chelators demonstrate antitumor efficacy through dual mechanisms: (1) iron depletion via extracellular efflux and intracellular sequestration; (2) ROS reduction and tumor suppressor gene activation (TP53, PTEN). Deferasirox inhibits breast/colorectal cancer proliferation in vitro and xenograft models [620, 621, 622], while modulating STAT3, TGF‐β, and Wnt signaling pathways [620, 622‐625].

Iron chelation protects against ferroptosis—an iron‐dependent LPO process. Lipophilic antioxidants (ferrostatin‐1, liproxstatin‐1) ameliorate cardiac IRI by reducing labile iron pools and ROS generation [626, 627]. Mechanistically, ferrostatin‐1 attenuates AS in *ApoE−/−* mice via GPX4 preservation and inflammatory pathway suppression [627, 628, 629], while liproxstatin‐1 improves myocardial recovery post‐I/R [630]. Clinical studies demonstrate chelators’ capacity to enhance mitochondrial function, reduce myocardial fibrosis, and improve cardiac output in iron‐overload cardiomyopathy [631, 632, 633, 634]

Elevated cerebral iron deposition exacerbates oxidative damage in AD and PD. Deferiprone reduces Aβ aggregation in AD models and substantia nigra iron content in PD, showing cognitive preservation potential [635, 636, 637]. In pantothenate kinase‐associated neurodegeneration and HD, chelation therapy decreases basal ganglia iron deposition [638].

Iron chelating agents show a variety of potential targets and therapeutic strategies in the treatment of diabetes [639]. Iron catalyzes AGE formation through metal‐catalyzed oxidation. Chelators (e.g., penicillamine) inhibit AGE generation and OS in diabetic models [640, 641]. Angiotensin‐converting enzyme inhibitors/angiotensin II receptor blockers such as ramipril and valsartan demonstrate renal protection via AGE reduction and antioxidant enzyme upregulation (e.g., SOD) [642].

### 5.4 Nrf2 activators

The TF Nrf2 orchestrates cellular antioxidant defenses through ARE‐mediated transcriptional activation of cytoprotective genes. This central regulatory role positions Nrf2 activators as promising therapeutics for OS‐associated pathologies.

Nrf2 activation mitigates oxidative mechanisms underlying cardiac dysfunction and atherogenesis. Pharmacological activation using diallyl disulfide enhances nuclear Nrf2 translocation and downstream antioxidant enzyme expression, demonstrating cardioprotective effects through apoptosis inhibition in preclinical models [643, 644, 645].

Nrf2 exhibits dual roles in carcinogenesis: While conferring antioxidant protection, constitutive activation promotes tumor survival and chemoresistance. Epigallocatechin gallate counteracts tumor progression through cyclooxygenase‐2/iNOS downregulation and MMP‐mediated invasion suppression [646, 647]. Dimethyl fumarate activates Nrf2‐dependent pathways to induce cancer cell apoptosis while inhibiting angiogenesis across multiple malignancies [648, 649, 650, 651, 652, 653]. Curcumin enhances treatment sensitivity in prostate, colorectal, and ovarian carcinomas through redox modulation [654, 655].

Nrf2 dysfunction exacerbates oxidative neuronal damage in AD and PD. Forsythoside A demonstrates multimodal neuroprotection through: (1) dopaminergic signaling potentiation; (2) iron homeostasis restoration; (3) NF‐κB pathway inhibition; (4) anti‐inflammatory cytokine induction [656].

Nrf2 activation preserves β‐cell function by countering oxidative insulin resistance. Sulforaphane prevents diabetic cardiomyopathy via ferritin/SLC7A11‐mediated ferroptosis inhibition in experimental cardiomyopathy models [657]. Oltipraz ameliorates hyperglycemia and pancreatic damage through Nrf2/HO‐1 axis activation, concurrently improving lipid metabolism and insulin secretion capacity [658].

### 5.5 NOX Inhibitors

NOX is a class of enzymes that are widely distributed across various cell types and primarily responsible for reducing molecular oxygen to superoxide anion, a critical process in the development of OS. The activation of NOX results in the production of large amounts of ROS and is closely linked to various diseases, including cardiovascular diseases, neurodegenerative diseases, diabetes, chronic inflammation, and cancer.

In cardiovascular diseases, the activation of NOX is closely associated with pathological conditions such as hypertension, AS, and heart failure. The overproduction of superoxide anions not only directly damages endothelial cells but also significantly reduces the biological activity of NO, culminating in endothelial dysfunction. Consequently, the development of NOX inhibitors to decrease superoxide anion levels has emerged as an effective strategy for improving cardiovascular function. For example, the NOX inhibitor VAS2870 has been shown to prevent reperfusion‐induced hypertension and improve outcomes in acute stroke treatment [659]. Furthermore, the specific inhibitor Nox2ds‐tat has demonstrated effectiveness in significantly reducing superoxide anion production and improving vascular contraction responses in experimental models of hypertension, highlighting the therapeutic potential of NOX inhibitors in treating cardiovascular diseases [660].

In the nervous system, the overactivation of NOX is similarly implicated in the OS associated with neurodegenerative diseases, such as AD and PD. Research suggests that the activation of NOX leads to excessive ROS production, resulting in neuronal death and neural damage. Inhibitors of NOX, such as apocynin, have been found to lower OS levels in neurons, providing protection for neural cells. The application of these inhibitors not only alleviates neuroinflammation and mitigates increases in ROS, NO, and TNF‐α levels but may also enhance cognitive function, positioning NOX as a potential therapeutic target in neurodegenerative diseases [661].

In the context of diabetes and its complications, NOX activation is critical in insulin resistance and pancreatic β‐cell dysfunction. Hyperglycemia promotes NOX activation, worsening OS, which can lead to abnormal insulin secretion and disrupted lipid metabolism. NOX inhibitors may improve β‐cell function and enhance insulin sensitivity by lowering ROS levels, thus participates in diabetes management [662]. For instance, NOX2 inhibitors have been shown to regulate ROS production and insulin secretion in the pancreatic β‐cells of diabetic mouse models, demonstrating beneficial metabolic effects and providing hope for the clinical treatment of diabetic patients [662, 663].

In chronic inflammation and cancer, NOX activity is frequently abnormally elevated, resulting in excessive ROS production that facilitates cell proliferation and tumor progression [664]. The use of NOX inhibitors can significantly diminish OS within the tumor microenvironment, thus inhibiting tumor cell growth and metastasis. Studies indicate that specific NOX inhibitors, such as apocynin, inhibit the translocation of p47phox, selectively preventing NOX2 activation, which in turn reduces O_2_•− production in vitro and exhibits anti‐inflammatory actions in vivo. Additionally, fulvene‐5 has demonstrated the capacity to inhibit both NOX2 and NOX4, successfully preventing neovascularization derived from endothelial cells in mouse models [664].

### 5.6 Mitochondrial Antioxidant

As the cellular powerhouses, mitochondria constitute both essential energy producers (via oxidative phosphorylation) and primary generators of ROS. Physiological ROS production becomes pathogenic when exceeding homeostatic thresholds under stress conditions, driving cellular damage and disease progression. This dual nature positions mitochondrial‐targeted antioxidants as promising therapeutics for cardiovascular, neurodegenerative, metabolic, and oncological disorders.

The mitochondrial antioxidant system comprises SOD, GPX, and GSH. Pharmacological enhancement of these endogenous enzymes, combined with exogenous antioxidant supplementation, effectively counteracts oxidative damage. In mild TBI models, the mitochondrial‐targeted peptide SS‐31 ameliorates OS by suppressing Nox4 activation and MDA production, while concurrently activating Nrf2–ARE signaling and inhibiting NF‐κB p65‐mediated inflammation [665]. SS‐31 further demonstrates renal protection in cisplatin‐induced nephrotoxicity through mtROS/NLRP3 pathway modulation [666].

MitoQ, a conjugate of ubiquinone and triphenylphosphonium, selectively accumulates in the mitochondria to inhibit LPO through radical scavenging [667, 668, 669, 670]. Preclinical studies have demonstrated its efficacy in preserving cardiac function following ischemia‐reperfusion, attenuating neurodegenerative pathology, and enhancing mitochondrial bioenergetics [670, 671, 672]. Additionally, Tiron complements this approach through dual mechanisms of metal chelation and ROS neutralization [670].

Hyperglycemia‐induced mitochondrial dysfunction aggravates insulin resistance by impairing β‐cell function through ROS production. Administration of SS‐31 has been shown to restore insulin sensitivity in the skeletal muscle of diet‐induced obese mice, underscoring the potential of mitochondrial antioxidants in diabetes management [673].

And now emerging strategies are now diversifying to address mitochondrial OS through novel mechanisms and molecules. Astaxanthin, a potent carotenoid antioxidant, exhibits superior membrane integration and free radical scavenging compared with conventional antioxidants. By restoring the activities of mitochondrial respiratory complexes II and III, astaxanthin prevents oxidative damage and skeletal muscle atrophy in various experimental models. Its membrane‐targeted antioxidant capacity enables sustained protection against LPO, thereby preserving mitochondrial bioenergetics and muscle integrity [674, 675, 676].

XJB‐5‐131, a mitochondria‐targeted antioxidant, has demonstrated broad therapeutic potential across various disease models. In HD transgenic mouse models, XJB‐5‐131 effectively restores mitochondrial function, improves locomotor performance, and enhances neuronal survival, thereby delaying weight loss and motor decline [677]. Mechanistically, XJB‐5‐131 acts as a mild uncoupler of oxidative phosphorylation within a 0.2–10 µM concentration range, reducing mtROS production without substantially compromising ATP synthesis [678]. Its nitroxide radical moiety enables it to scavenge electrons escaping from the ETC, suppressing O_2_•− and H_2_O_2_ generation in a manner analogous to SOD, while preserving normal electron flow through the ETC and maintaining complex V activity [678]. Moreover, XJB‐5‐131 specifically protects mtDNA from oxidative damage, preserving ETC integrity and preventing cellular decline associated with mitochondrial dysfunction, particularly in muscle tissue [676].

## 6 Clinical Applications of Antioxidant Therapies

Contemporary therapeutic regimens targeting OS pathophysiology, encompassing pharmacological modulators of ROS signaling and mitochondrial redox homeostasis, have been increasingly integrated into clinical management algorithms across diverse disease spectra (Table 1).

**TABLE 1:** Oxidative stress‐related therapeutic strategies and applications.

Disease category	Drug/compound	Mechanism	Related diseases/applications	References
Neurodegenerative diseases	Coenzyme Q10	Improves inflammatory markers, reduces oxidative damage to neurons	Multiple sclerosis	[677, 678]
	N‐acetylcysteine	Promotes glutathione synthesis, enhances endogenous antioxidant defenses, alleviates motor symptoms	Parkinson's disease	[679]
	Deferoxamine	Chelates iron, improves iron overload and oxidative stress	Acute ischemic stroke	[680]
	Berberine	Antioxidant and anti‐inflammatory actions (reduces TNF‐α, IL‐1β, IL‐6; increases IL‐10 and TGF‐β), enhances antioxidant enzyme activity (SOD, CAT, GPX)	Parkinson's disease, neurodegenerative diseases	[681, 682]
	AGEs/RAGE inhibitors	Inhibit AGEs‐RAGE interactions, block NF‐κB pathway, reduce ROS and inflammatory factors	Alzheimer's disease	[683]
Cardiovascular diseases	N‐acetylcysteine	Regenerates intracellular antioxidants (glutathione precursor), reduces oxidative stress	Coronary artery disease, myocardial infarction	[684, 685]
	Quercetin, resveratrol	Antioxidant and anti‐inflammatory effects, restores endothelial function	Atherosclerosis	[686, 687]
	MitoTEMPO	Targets mitochondria, mimics SOD activity, reduces ROS generation	Heart failure, cardiac hypertrophy	[688]
	SS‐31	Reduces myocardial infarct size in ischemia–reperfusion injury, improves mitochondrial function	Myocardial ischemia, heart failure	[689, 690, 691, 692]
Cancer	Coenzyme Q10	Enhances antioxidant capacity, reduces oxidative stress and inflammation	Liver cancer (postoperative adjuvant therapy)	[693]
	Quercetin, resveratrol	Antioxidant activity, adjuvant in antitumor therapy	Various cancers	[694]
	Doxorubicin	Induces apoptosis in tumor cells through ROS accumulation	Metastatic breast cancer	[695]
Metabolic diseases	N‐acetylcysteine	Promotes wound healing in diabetes	Diabetic complications	[646]
	Resveratrol	Modulates LKB1–AMPK pathway to inhibit Nox2/p67, improves endothelial function	Type 2 diabetes	[647, 648]
	Biguanides (e.g., metformin)	Inhibits mitochondrial fission, reduces ROS through Nur77/NR4A1 receptor‐mediated antioxidant effects	Diabetes, atherosclerosis	[649, 650, 651, 652]
	GLP‐1 receptor agonists	Inhibits ROS, exerts antiapoptotic effects, improves endothelial function	Diabetes, cardiovascular diseases	[653, 654, 655, 656]
	SGLT2 inhibitors (e.g., dapagliflozin)	Blocks endothelial glucose transport, activates AMPK pathway to reduce oxidative stress	Diabetes, cardiovascular complications	[657, 658]
Emerging strategies	Mitochondrial adaptive regulation	Modulates mitochondrial function to cope with nutrient stress	Metabolic disease vascular lesions	[659]
	Nrf2 pathway activation	Promotes transcription of antioxidant genes (e.g., SOD, CAT)	Metabolic diseases	[660]

### 6.1 Advances in Clinical Treatment of Neurodegenerative Disease

Antioxidants, such as CoQ10, have demonstrated potential in improving neuronal survival and function. Clinical studies suggest that CoQ10 can enhance inflammatory markers in multiple sclerosis, aiding in the reduction of oxidative damage to neurons [679, 680]. NAC, one of the most commonly utilized antioxidants, has shown protective effects in clinical trials involving PD patients. NAC boosts endogenous antioxidant defenses by promoting GSH synthesis, thereby alleviating motor symptoms and enhancing patients’ quality of life [681]. Additionally, the use of iron chelators, such as DFO, has progressed in central nervous system disorders. Research indicates that DFO can improve the prognosis of individuals with acute ischemic stroke by ameliorating iron overload and OS [682].

Recently, berberine (BBR), a natural compound, has revealed multifaceted potential in treating neurodegenerative diseases. BBR not only exhibits antioxidant properties but also modulates inflammatory responses. Studies indicate that BBR can lower proinflammatory cytokines (e.g., TNF‐α, IL‐1β, IL‐6) while enhancing the expression of anti‐inflammatory cytokines (e.g., IL‐10 and TGF‐β) [683]. Furthermore, BBR promotes neuroprotection by increasing the activity of antioxidant enzymes (such as SOD, CAT, and GPX) and reducing LPO and DNA damage [683]. In treating PD, clinical investigations have shown that administering 0.5 g/day of BBR significantly augments serum DA patients with hyperlipidemia, indicating its potential benefits in PD treatment [684].

The roles of AGEs and their receptor RAGE in neurodegenerative diseases have also attracted attention. The accumulation of AGEs is closely related to the pathological processes underlying AD, including protein cross‐linking, OS, and neuronal death. Research demonstrates that the interaction between AGEs and RAGE triggers the activation of the NF‐κB signaling pathway, leading to the release of proinflammatory cytokines and ROS production, which exacerbates neurodegenerative damage [685]. Therapeutic strategies targeting AGEs and RAGE are under development, such as inhibiting RAGE signaling or reducing AGE accumulation to mitigate OS and inflammatory responses. These strategies possess potential therapeutic value for AD and other neurodegenerative disorders.

### 6.2 Advances in Clinical Treatment of Cardiovascular Disease

OS plays a significant role in the development of cardiovascular diseases, particularly in conditions such as AS and heart failure. Several clinical treatment strategies have been developed to target this mechanism, aiming to reduce the incidence of cardiovascular events. NAC is recognized as an effective antioxidant that alleviates OS by regenerating intracellular antioxidants, particularly by acting as a precursor for GSH. Early clinical trials suggest that NAC not only significantly improves clinical symptoms in patients with coronary heart disease but also, when combined with low‐dose nitroglycerin, effectively reduces the area of myocardial infarction and enhances myocardial salvage, thereby demonstrating its potential value in the treatment of heart disease [686, 687].

Plant‐derived polyphenolic compounds, such as quercetin and polyphenols found in red wine, have also been shown to confer significant cardiovascular protective effects. Quercetin has been effective in reducing inflammatory mediators and restoring endothelial function, and its antioxidant and anti‐inflammatory properties make it a key candidate for treating cardiovascular diseases [688]. In addition, polyphenols present in olive oil have been demonstrated to significantly enhance endothelial function and improve protective effects against AS [689].

With the growing emphasis on the benefits of traditional antioxidants, research on mitochondrial‐targeted antioxidants has garnered interest. Compounds such as MitoTEMPO, MitoQ, and SS‐31 are designed to specifically target mitochondria, reducing ROS generation and thereby mitigating cardiac injury. MitoTEMPO, which mimics the action of SOD, has exhibited strong cardioprotective effects in animal models, especially in studies of heart failure and catecholamine‐induced cardiac arrest, effectively decreasing ROS levels in both the mitochondrial and cytoplasmic compartments of failing cardiomyocytes [690]. Moreover, chronic administration of MitoTEMPO may prevent the onset of heart failure; notably, administering it after the onset of myocardial hypertrophy also confers protective effects [690]. SS‐31 has demonstrated significant efficacy in reducing myocardial injury within studies of IRI. In models of cardiac IRI, the administration of SS‐31 at the onset of ischemia or even prior to reperfusion in mouse, rat, guinea pig, and rabbit models significantly decreases infarct size. Additionally, in the context of heart failure, SS‐31 plays a crucial role by attenuating cardiac hypertrophy in transverse aortic constriction mice, reducing fibrosis, improving cardiac function, and significantly diminishing the severity of mitochondrial ultra‐structural and proteomic alterations [694].

### 6.3 Advances in Clinical Treatment of Cancer

OS demonstrates dual oncogenic properties in cancer treatment—facilitating tumor proliferation while inducing apoptotic pathways. This paradoxical nature has spurred development of ROS‐scavenging strategies. A single‐blind randomized controlled trial in hepatocellular carcinoma patients revealed that 12‐week postoperative CoQ10 supplementation significantly enhanced antioxidant capacity while reducing OS and inflammation, indicating its potential clinical utility in cancer management [695].

Natural compounds including quercetin and resveratrol exhibit potent antioxidant activities, demonstrating adjuvant therapeutic potential in oncology [696]. The clinical application of pro‐oxidant DOX exemplifies redox imbalance‐mediated apoptosis induction through ROS accumulation. Phase III trials demonstrate favorable activity and tolerability of pegylated liposomal DOX in metastatic breast cancer [697].

### 6.4 Advances in Clinical Treatment of Metabolic Disease

Antioxidants like NAC adjunctively improve diabetic wound healing in rodent models [698]. Resveratrol, a natural polyphenol, modulates antioxidant pathways through ROS/RNS scavenging and redox homeostasis regulation. In diabetic murine models, it activates LKB1–AMPK signaling to suppress NOX2/p67 expression, ameliorating endothelial dysfunction [699]. Clinical studies confirm capacity of resveratrol to enhance glycemic control in type 2 diabetes mellitus patients via insulin resistance reduction and chronic inflammation mitigation [700].

Modern antidiabetic agents exhibit pleiotropic effects beyond glucose regulation, including OS modulation for vascular protection. Biguanides attenuate atherogenesis through mitochondrial fission inhibition and ROS reduction [701, 702], with antioxidant mechanisms partially mediated via Nur77/NR4A1 nuclear receptor interactions [703]. Clinical trials validate their efficacy in diabetes prevention among high‐risk populations [704]. Glucagon‐like peptide‐1 (GLP‐1) receptor agonists such as exenatide and liraglutide improve endothelial function through ROS suppression and antiapoptotic effects, demonstrating cardiovascular benefits [705, 706, 707] alongside proven weight reduction and glycemic control [708]. Sodium‐glucose cotransporter 2 (SGLT2) inhibitors like dapagliflozin mitigate OS via endothelial glucose transport blockade and AMPK activation [709, 710].

Emerging therapeutic strategies are expanding beyond conventional antioxidants to explore novel targets, including the modulation of mitochondrial adaptation in VSMCs under nutrient stress and the activation of the Nrf2 pathway to enhance antioxidant gene transcription. These innovative approaches show great promise in addressing the pathophysiology of metabolic diseases [711, 712].

In response to the previously presented information, we have developed a comprehensive table that illustrates the advancements in clinical treatments targeting OS across various human diseases. This table consolidates relevant pharmacological interventions, highlighting their mechanisms of action and associated therapeutic applications.

The management of OS has emerged as an essential strategy in treating neurodegenerative disorders, cardiovascular diseases, cancers, and metabolic diseases. Notably, compounds such as NAC and CoQ10 have demonstrated significant efficacy in enhancing antioxidant defenses and mitigating oxidative damage, thereby improving clinical outcomes in conditions like PD, coronary artery disease, and liver cancer. Furthermore, innovative approaches, including the modulation of mitochondrial function and activation of the Nrf2 signaling pathway, represent promising avenues for addressing OS‐related pathologies.

Based on a comprehensive review of the existing literature, we have systematically summarized and tabulated OS‐related therapeutic strategies and their clinical applications.

## 7 Conclusion and Prospects

### 7.1 Conclusion

OS is a fundamental biological phenomenon characterized by an imbalance between ROS production and antioxidant defenses, contributing to cellular damage and the pathogenesis of numerous diseases. While ROS play essential roles in physiological processes such as immune regulation and cell signaling, excessive accumulation leads to oxidative damage to lipids, proteins, and DNA, driving the progression of neurodegenerative, cardiovascular, oncological, hepatic, and renal diseases. The intricate crosstalk among ROS, antioxidant systems, and cellular signaling pathways highlights the complexity of OS and its widespread impact on human health.

Recent research has expanded our understanding of OS‐related disease mechanisms and identified promising therapeutic targets, including antioxidants, ROS scavengers, and inhibitors of oxidative signaling pathways. Clinical and preclinical studies suggest that targeting OS through pharmacological and molecular interventions can mitigate disease progression and improve patient outcomes. However, despite these advancements, there remain significant gaps in our knowledge regarding the systemic effects of OS across different organ systems, as well as the long‐term efficacy and safety of antioxidant‐based therapies.

To address these challenges, future research should prioritize the development of precision medicine strategies that integrate OS‐targeting therapies with personalized treatment approaches. Investigations into novel antioxidant compounds, mitochondrial‐targeted therapies, and genetic interventions hold great potential for advancing disease management. Furthermore, interdisciplinary collaboration between molecular biologists, clinicians, and bioinformaticians is essential to unravel the complex regulatory networks underlying OS and translate these insights into effective therapeutic applications. By bridging these gaps, a more comprehensive and integrative approach to OS‐related diseases can be developed, ultimately improving clinical outcomes and quality of life for patients worldwide.

### 7.2 Future Perspectives

Given the widespread role of OS in human diseases, therapeutic strategies targeting oxidative damage have gained increasing attention. Antioxidants, including CoQ10, NAC, and mitochondrial‐targeted agents such as MitoQ and SS‐31, have shown promise in mitigating OS‐related damage across multiple organ systems. Additionally, emerging evidence suggests that plant‐derived polyphenols, AGE–RAGE inhibitors, and iron chelators hold therapeutic potential in neurodegenerative and cardiovascular diseases. Despite these advances, translating these findings into clinical applications remains challenging due to variability in patient response, potential side effects, and the complexity of redox signaling in disease pathogenesis.

A crucial future direction is the refinement of OS‐targeting therapies through precision medicine approaches. Advances in genomics, metabolomics, and bioinformatics offer new opportunities to tailor OS‐related interventions based on an individual's genetic background, disease stage, and metabolic profile. The integration of systems biology approaches may also provide deeper insights into the interactions between OS and other cellular processes, such as inflammation, autophagy, and apoptosis, thereby enabling the development of multitargeted therapeutic strategies.

Furthermore, while antioxidants are widely used to counteract OS, their potential toxicity and long‐term effects require careful evaluation. High‐dose antioxidant supplementation has been associated with paradoxical pro‐oxidant effects and disruptions in cellular homeostasis. Therefore, future studies should focus on optimizing antioxidant dosages, identifying patient subgroups that would benefit most from antioxidant therapy, and investigating the impact of prolonged antioxidant use on organ function and systemic health. Developing novel redox modulators that fine‐tune, rather than completely inhibit, OS pathways may offer a more effective and safer therapeutic approach.

#### 7.2.1 Toxicity of Antioxidants to Organ Systems

In recent decades, antioxidants have gained recognition for their protective roles against OS caused by ROS, providing defense against a variety of diseases. However, recent studies have highlighted the complexities associated with the use of antioxidants, especially when administered at high doses or inappropriately, as they may result in potential toxic effects across various organ systems in the body.

The toxicity of antioxidants is notably dose dependent. For example, investigations using the antioxidant AO2246 in zebrafish larvae have demonstrated that its toxic effects become more pronounced with increasing doses [[Link], [Link], [Link]]. Moreover, excessive use of antioxidants can lead to “antioxidant stress,” disrupting the physiological balance between peroxides and antioxidants [714]. Such an imbalance may result in the conversion of vitamin C into a pro‐oxidant under certain conditions, initiating harmful lipid oxidation. Additionally, superoxide anions and other free radicals can exacerbate toxicity by interfering with iron metabolism, thereby intensifying oxidative damage to cells [714].

Maintaining a dynamic balance between ROS and antioxidants is essential for overall health. Moderate levels of ROS play a role in regulating physiological functions and promoting disease prevention. Nevertheless, current research primarily focuses on the short‐term effects of antioxidants and their anticipated therapeutic benefits in disease management. Therefore, a systematic assessment of the risks associated with long‐term usage and potential cumulative toxicity is necessary.

#### 7.2.2 Application of OS in Precision Medicine

Recent progress in precision medicine has leveraged OS‐related molecular pathways to develop innovative and highly targeted therapeutic strategies across multiple disease contexts.

In oncology, predictive gene signatures derived from OS‐associated mitochondrial gene sets and oxidative metabolism‐related genes have enabled patient stratification into molecular subtypes with distinct sensitivities to chemotherapeutic and targeted agents [715, 716]. The integration of these signatures with immune checkpoint profiling uncovers OS as a modulator of tumor immune microenvironments, informing combinational approaches that synergize oxidative metabolism modulation with immunotherapy [715, 716].

Mechanistic innovations in nanomedicine exemplify the translational potential of OS‐targeted therapies. The development of DNA nanostructure platforms, particularly tetrahedral framework nucleic acids functionalized with aptamers targeting overexpressed receptors (e.g., CD44 on injured renal tubular epithelial cells), enables highly specific cellular targeting and intracellular delivery of antioxidant agents such as baicalein. This approach overcomes limitations imposed by conventional antioxidants’ systemic distribution and rapid clearance by enhancing stability, renal accumulation, and cellular internalization [717]. Functionally, these nanocarriers attenuate mtROS, restore membrane potential homeostasis, and inhibit apoptosis by modulating redox‐sensitive signaling cascades including NF‐κB and NLRP3 inflammasome pathways. The resultant multimodal effects—combining antioxidation, anti‐inflammatory action, and preservation of cellular integrity—demonstrate superior therapeutic outcomes in AKI models when compared with untargeted treatments [717].

In addition, the correlation between OS gene signatures and immune cell infiltration profiles across cancer types provides avenues for integrating oxidative metabolism modulators with immune checkpoint inhibitors [718]. This combinatorial precision strategy aims to reverse immunosuppressive microenvironments fostered by OS‐induced metabolic reprogramming, thus enhancing antitumor immunity in patient subsets defined by OS‐related molecular phenotypes [718].

Collectively, these advances highlight the evolving landscape of OS‐centered precision therapeutics, which synergize molecular profiling, targeted delivery technologies, and mechanistic understanding of redox biology.

#### 7.2.3 The Integration of Multiomics and Systems Biology Approaches

The integration of multiomics and systems biology approaches has also greatly advanced our understanding of OS in complex diseases and biological processes. By combining genomic, epigenomic, transcriptomic, proteomic, and metabolomic data, these integrative methods enable the identification of key OS‐related genes, pathways, and molecular mechanisms across diverse conditions such as gastroesophageal reflux disease, atrial fibrillation, pancreatic cancer, parasitic infections, and MDD [719, 720, 721]. The use of Mendelian randomization, machine learning, and immune cell profiling further strengthens causal inference and elucidates the interplay between OS, immune responses, metabolic dysregulation, and cellular damage [722]. This holistic strategy uncovers novel biomarkers and potential therapeutic targets by revealing how OS contributes to disease pathogenesis through complex gene‐environment and molecular network interactions. Despite challenges such as tissue specificity, data coverage limitations, and the need for functional validation, multiomics integration represents a powerful framework for deciphering OS‐related biology and informing precision medicine approaches.

## Author Contributions

S.L. and J.L. contributed equally to this work and share first authorship. S.L. and J.L. conceptualized and designed the study. Y.W. and F.D. conducted the literature review and contributed to manuscript drafting. J.L. and Z.D. critically revised the manuscript for intellectual content. S.L. and J.L. wrote the initial draft, while F.D. and Z.D. refined the manuscript and provided additional insights. Z.D. supervised the study and approved the final version for submission. All authors have read and approved the final manuscript.

## Ethics Statement

The authors have nothing to report.

## Conflicts of Interest

The authors declare no conflicts of interest.

## Data Availability

No new data were generated or analyzed in this study. Data sharing is not applicable to this article.
